# Non‐Fermi‐Liquid Behavior of Superconducting SnH_4_

**DOI:** 10.1002/advs.202303622

**Published:** 2023-08-25

**Authors:** Ivan A. Troyan, Dmitrii V. Semenok, Anna G. Ivanova, Andrey V. Sadakov, Di Zhou, Alexander G. Kvashnin, Ivan A. Kruglov, Oleg A. Sobolevskiy, Marianna V. Lyubutina, Dmitry S. Perekalin, Toni Helm, Stanley W. Tozer, Maxim Bykov, Alexander F. Goncharov, Vladimir M. Pudalov, Igor S. Lyubutin

**Affiliations:** ^1^ Shubnikov Institute of Crystallography Federal Scientific Research Center Crystallography and Photonics Russian Academy of Sciences 59 Leninsky Prospekt Moscow 119333 Russia; ^2^ Center for High Pressure Science and Technology Advanced Research (HPSTAR) Beijing 100193 China; ^3^ V. L. Ginzburg Center for High‐Temperature Superconductivity and Quantum Materials P. N. Lebedev Physical Institute Russian Academy of Sciences Moscow 119991 Russia; ^4^ Skolkovo Institute of Science and Technology Bolshoy Boulevard, 30/1 Moscow 121205 Russia; ^5^ Center for Fundamental and Applied Research Dukhov Research Institute of Automatics (VNIIA) st. Sushchevskaya, 22 Moscow 127055 Russia; ^6^ Laboratory of Computational Materials Discovery Moscow Institute of Physics and Technology 9 Institutsky Lane Dolgoprudny 141700 Russia; ^7^ A.N. Nesmeyanov Institute of Organoelement Compounds Russian Academy of Sciences 28 Vavilova str. Moscow 119334 Russia; ^8^ Hochfeld‐Magnetlabor Dresden (HLD‐EMFL) and Würzburg‐Dresden Cluster of Excellence Helmholtz‐Zentrum Dresden‐Rossendorf (HZDR) 01328 Dresden Germany; ^9^ National High Magnetic Field Laboratory Florida State University Tallahassee Florida 32310 USA; ^10^ Institute of Inorganic Chemistry University of Cologne 50939 Cologne Germany; ^11^ Earth and Planets Laboratory Carnegie Institution for Science 5241 Broad Branch Road NW Washington DC 20015 USA; ^12^ HSE Tikhonov Moscow Institute of Electronics and Mathematics National Research University Higher School of Economics 20 Myasnitskaya ulitsa Moscow 101000 Russia

**Keywords:** high pressure, non‐Fermi‐liquid, superconductivity, tin hydrides

## Abstract

The chemical interaction of Sn with H_2_ by X‐ray diffraction methods at pressures of 180–210 GPa is studied. A previously unknown tetrahydride SnH_4_ with a cubic structure (*fcc*) exhibiting superconducting properties below *T*_C_ = 72 K is obtained; the formation of a high molecular *C*2/*m*‐SnH_14_ superhydride and several lower hydrides, *fcc* SnH_2_, and *C*2‐Sn_12_H_18_, is also detected. The temperature dependence of critical current density *J*_C_(T) in SnH_4_ yields the superconducting gap 2Δ(0) = 21.6 meV at 180 GPa. SnH_4_ has unusual behavior in strong magnetic fields: *B,T*‐linear dependences of magnetoresistance and the upper critical magnetic field *B*_C2_(T) ∝ (*T*_C_ – *T*). The latter contradicts the Wertheimer–Helfand–Hohenberg model developed for conventional superconductors. Along with this, the temperature dependence of electrical resistance of *fcc* SnH_4_ in non‐superconducting state exhibits a deviation from what is expected for phonon‐mediated scattering described by the Bloch‐Grüneisen model and is beyond the framework of the Fermi liquid theory. Such anomalies occur for many superhydrides, making them much closer to cuprates than previously believed.

## Graphical abstract

Chemical reaction of Sn with H_2_ at 180 GPa leads to a novel tetrahydride SnH_4_, which has the superconducting gap 2Δ(0) = 23 meV and the critical temperature *T*_C_ of 72 K. SnH_4_ demonstrates unusual behavior including *B,T*‐linear dependences of magnetoresistance and the critical magnetic field *B*_C2_(T) ∝ (*T*_C_ – *T*), making it much closer to unconventional superconductors than previously believed.

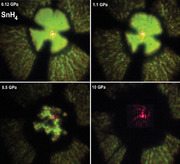

## 1 Introduction

The study of high‐temperature superconductivity is one of the most important problems in condensed matter physics. Compressed polyhydrides are promising high‐temperature superconductors, which can be obtained at pressures of 1–2 Mbar. Since 2015, many remarkable hydride superconductors have been experimentally discovered. These are H_3_S (with *T*_C_ = 200 K),^[^ ^1^ ^]^ LaH_10_ (*T*_C_ = 250 K),^[^ ^2^, ^3^ ^]^ ThH_9_ (*T*_C_ = 146 K), ThH_10_ (*T*_C_ = 161 K),^[^ ^4^ ^]^ YH_6_ (*T*_C_ = 226 K),^[^ ^5^, ^6^ ^]^ and YH_9_ (*T*_C_ = 243 K),^[^ ^6^ ^]^ CeH_9_ and CeH_10_ (*T*_C_ = 110–120 K),^[^ ^7^ ^]^ CaH_6_ (*T*_C_ = 215 K).^[^ ^8^, ^9^ ^]^ Most of the superconducting polyhydrides contain elements of groups IIIA, IVB with d^0^‐d(f)^1^ valent electrons,^[^ ^10^ ^]^ whereas polyhydrides of *p*‐elements, and in particular Sn, have not been studied enough.

In the last 10 years, the chemistry of polyhydrides of group IV elements (C, Si, Ge, Sn, Pb) has been intensively studied by means of the density functional theory (DFT). The experimental synthesis of such polyhydrides is carried out at high‐pressure in diamond anvil cells (DACs). C, Si, and Ge hydrides are mainly low‐symmetry molecular covalent compounds with moderate superconducting properties^[^ ^11^, ^12^, ^13^ ^]^ The higher molecular silicon polyhydride SiH_4_(H_2_)_2_ was observed in experiments of Strobel et al. and Wang et al.^[^ ^14^, ^15^ ^]^ Resistive transitions with *T*_C_ up to 79 K for silicon polyhydrides were obtained under high pressure by the group of M. Eremets et al.^[^ ^16^ ^]^ Theoretical calculations for Ge hydrides at 200–300 GPa, predicted stable low symmetry phases of GeH_3_ and Ge_3_H_11_, and various polymorphic modifications of the well‐known GeH_4_.^[^ ^13^, ^17^ ^]^ A new molecular compound, possibly GeH_4_(H_2_)_2_, was obtained experimentally at 7.5 GPa.^[^ ^18^ ^]^ According to theoretical calculations, metallic *P*2_1_ */c*‐GeH_4_(H_2_)_2_ is a superconductor with *T*_C_ of 76–90 K at 250 GPa.^[^ ^19^ ^]^

The increase in metallicity of Sn to Pb results in the appearance of thermodynamically stable polyhydrides in the high‐pressure phase diagram. For instance, theory predicts the formation of *C*2/*m*‐SnH_12_, *C*2/*m*‐SnH_14_,^[^ ^20^ ^]^ and various PbH_4_, PbH_6_, and PbH_8_^[^ ^21^, ^22^, ^23^ ^]^ lead polyhydrides under high pressure. However, attempts to synthesize Pb polyhydrides have been unsuccessful to date.^[^ ^24^ ^]^ The recent observation by Hong et al^[^ ^25^ ^]^ of a sharp drop in electrical resistance in unidentified Sn polyhydride at 71 K motivated us to investigate in detail the structure and superconducting properties of Sn polyhydrides under high pressure.

Three series of XRD experiments were carried out at different synchrotron facilities such as European Radiation Synchrotron Facility (ESRF) in 2017, Positron‐Electron Tandem Ring Accelerator (PETRA) in 2020 and Advanced Photon Source (APS) in 2022. At pressures around 160–210 GPa, we observed X‐ray diffraction patterns of the studied samples, which are attributed to different Sn hydrides formed. Most X‐ray diffraction (XRD) patterns have a characteristic set of cubic (*Fm* $\bar{3}$ *m*) reflections. In the experiment performed at APS, a single‐crystal XRD at a pressure of ≈190 GPa was carried out. An analysis of the diffraction pattern confirmed the cubic structure (*Fm* $\bar{3}$ *m*) of the Sn sublattice in SnH_4_. As we have found, tin tetrahydride is a non‐Fermi liquid metal, which demonstrates anomalous behavior of electrical resistance, *T*‐linear upper critical magnetic field, and H‐linear magnetoresistance in a wide range of temperatures and magnetic fields.

## 2 Results

### 2.1 High‐Pressure Synthesis of SnH4 with a Cubic (fcc) Structure

In this work, tin polyhydrides were synthesized and studied by X‐ray diffraction. The previously unknown tetrahydride SnH_4_ with a cubic structure (*fcc*) was synthesized at high pressures in diamond anvil cells in two different ways. In the first approach, we used the reaction of solutions of SnCl_4_ and LiAlH_4_. Gaseous stannane SnH_4_ was emanated, then condensed upon cooling with liquid nitrogen.^[^ ^26^ ^]^ Using a cryogenic filling at a pressure of ≈2.0 bar, liquefied SnH_4_ was supplied through a thin capillary into the working volume of the DAC, and then compressed to high pressure at room temperature (Figure S7, Supporting Information). Monitoring the successful loading of gaseous SnH_4_ and its compression provided additional confirmation of the hydrogen content in the resulting high‐pressure phase *fcc* SnH_4_. In the second approach, SnH_4_ was synthesized during the interaction of pre‐compressed piece of metallic Sn and hydrogen produced by laser heating of NH_3_BH_3_ under high pressure. A table summarizing all the performed experiments can be found in the Tables S1 and S5 (Supporting Information).

**Figure 1:**
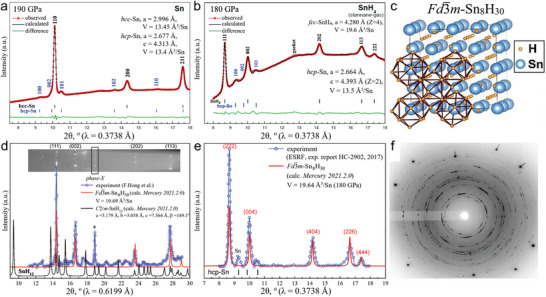
X‐ray diffraction patterns and the Le Bail refinements of the unit cell parameters of a) Sn at 190 GPa in DAC S1, and b) SnH_4_ and *hcp* Sn obtained after compression of gaseous stannane to 180 GPa in DAC S2 (ESRF‐2017). c) The crystal structure of theoretical model, *Fd* $\bar{3}$ *m*‐Sn_8_H_30_, which best fits the experimental pattern. d) XRD pattern of Sn hydride obtained by Hong et al.^[^ ^25^ ^]^ (blue curve, 206 GPa) and its fit by *Fd* $\bar{3}$ *m*‐Sn_8_H_30_ model (red curve). Asterisk indicate uninterpreted reflection (phase X). Obviously, the diffraction pattern does not correspond to the *C*2/*m*‐SnH_12_ structure (black curve) proposed by Hong et al. e) Fitting of the experimental XRD pattern after background subtraction (ESRF‐2017) using theoretical model *Fd* $\bar{3}$ *m*‐Sn_8_H_30_ (red curve) and *hcp* Sn phase (black dashes). It gives much better result than *C*2/*m*‐SnH_12_. f) Typical X‐ray diffraction pattern of *fcc* SnH_4_ at 170 GPa (PETRA‐2020, DAC D2).

Let us first consider the results of the initial experiment we performed at the ID27 beamline of ESRF in 2017 (**Figure** **1**). Our preliminary study of the compression of pure Sn (Figure 1a, DAC S1) showed that at 190 GPa it exists in two modifications: hexagonal (*hcp*) and cubic (*bcc*), the latter one is dominant. In fact, the phase transition *bcc* → *hcp* starts already at 160 GPa.^[^ ^27^ ^]^ However, the enthalpies of formation of these modifications differ very little (according to DFT calculations it is Δ*H* = 9.5 meV per atom), which explains the very low rate of this transformation. As follows from the XRD pattern in Figure 1a, at 190 GPa, the Sn unit cell volume is V(*Im* $\bar{3}$ *m*‐Sn) = 26.9 Å^3^, which agrees with results of a previous publication.^[^ ^28^ ^]^ Interesting, but the unit cell volumes of *hcp* Sn and *bcc* Sn are very similar.

During the experiment with stannane, frozen SnH_4_ was placed in DAC S2 and compressed to 180 GPa. It was found that with an increase in pressure, rapid metallization of SnH_4_ occurs at ≈10 GPa (Figure S9, Supporting Information), accompanied by the disappearance of the Raman signal. At ≈160 GPa, a new cubic modification of SnH_4_ is formed. Figure 1b shows that the Bragg peaks of the experimental XRD patterns of SnH_4_ at pressures 160–180 GPa are best indexed by a face‐centered cubic lattice (*fcc)* with unit cell volume 19.6 Å^3^/Sn at 180 GPa. The obtained XRD patterns also contain an impurity (broadened peaks at 2θ = 9.5° and 10.2°) which can be attributed to *hcp* Sn formed during SnH_4_ dissociation when loaded and compressed in the DAC S2. Stannane is thermodynamically unstable and under ambient conditions it gradually decomposes to Sn and H_2_.

Compressed Sn hydrides were also experimentally studied by Hong et al.^[^ ^25^ ^]^ The authors obtained a similar X‐ray diffraction pattern (Figure 1d, the only XRD pattern in Ref. [25]), indexed as *C*2/*m*‐SnH_12_ (*a* = 5.179 Å, *b* = 3.038 Å, *c* = 7.364 Å, *β* = 149.11^o^). This structure was previously predicted by DFT methods.^[^ ^20^ ^]^ It can be seen that the experimental XRD clearly does not match the theoretically predicted XRD of SnH_12_ (black curve in Figure 1d). At the same time, the experimental XRD pattern (blue curve in Figure 1d) fits well with the theoretical model of *fcc* SnH_4_, namely *Fd* $\bar{3}$ *m*‐Sn_8_H_30_ (= SnH_3.75_, red curve in Figure 1d), which is discussed in details in the Supporting Information (“Crystal structure search” section). The unit cell volume of the Sn_8_H_30_ is *V* = 19.64 Å^3^/Sn (or 628.68 Å^3^ for Z = 32) at 180 GPa. Using the data obtained in the ESRF‐2017 experiment, we refined unit cell parameters of *Fd* $\bar{3}$ *m*‐Sn_8_H_30_, which were found to be in a good agreement with the results of DFT calculations (Tables S5 and S8, Supporting Information).

### 2.2 Synthesis of Molecular SnH14

The next experiments were performed at the P02.2 PETRA III beamline in 2020. As has already been shown, stannane compression leads to the formation of mixtures of *hcp* Sn and cubic SnH_4_. This indicates an insufficient amount of hydrogen for the complete conversion of Sn to polyhydrides. Therefore, to obtain tin hydrides with a higher hydrogen content (similar to SrH_22_^33^ and BaH_12_^34^), we decided to use ammonia borane NH_3_BH_3_ (AB) as a source of H_2_ in DACs D2 and M2.

**Figure 2:**
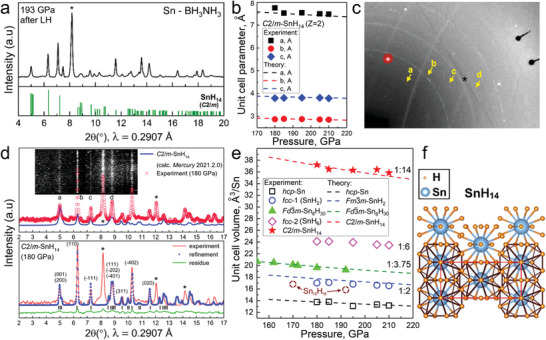
X‐ray diffraction patterns and the Le Bail refinements of the unit cell parameters of Sn hydrides in DACs D2 and M2. a) XRD pattern of SnH_x_ in NH_3_BH_3_ media after the laser heating at 193 GPa. Predicted XRD of *C*2/*m*‐SnH_14_ is shown on the bottom panel. b) Experimental and theoretical dependences of the unit cell parameters on the pressure for *C*2/*m*‐SnH_14_. с) Typical diffraction pattern at 180 GPa (PETRA‐2020, λ = 0.2904 Å). Asterisks denote uninterpreted peaks, “a–d” denote main reflections from SnH_14_. d) Comparison of experimental and calculated XRD reflection intensities for SnH_14_ (“a‐d”)_._ Inset: diffraction image (“cake”). Bottom panel: the Le Bail refinement of *C*2/*m*‐SnH_14_. Unidentified reflections are marked by asterisks. The experimental data, fitted line, and residues are shown in red, blue, and green, respectively. e) Pressure–unit cell parameters diagram for all tin hydrides synthesized in our experiments: stars, rhombuses, triangles, circles, and squares show the experimental data, lines depict the theoretical calculations. f) Crystal structure of SnH_14_ polyhydride.

The use of AB has yielded interesting results. We found that in addition to SnH_4_, several new compounds are formed, whose XRD patterns mostly correspond to the higher molecular polyhydride *C*2/*m*‐SnH_14_ predicted in 2016^[^ ^29^ ^]^ (**Figure** **2**). The refinements of the unit cell parameters of synthesized SnH_14_ (Figure 2a,b,d) are in a good agreement with the theoretical DFT calculations (Table S9, Supporting Information).

This polyhydride has an orthorhombic *Immm‐*Sn sublattice and a hydrogen sublattice, consisting of H_2_ (*d*_HH_ = 0.833 Å) and H_3_ (*d*_HH_ = 0.92 Å) fragments. According to molecular dynamics modeling, *C*2/*m*‐SnH_14_ is dynamically stable in the anharmonic approximation (Figure S13, Supporting Information). This is unusual for molecular polyhydrides, but SnH_14_ exhibits the properties of a typical metal with a high density of electronic states at the Fermi level (DOS). The DOS projection on hydrogen atoms, *N*_F_(H) = 0.32 states per eV per f.u., is larger than that on Sn atoms (*N*_F_(Sn) = 0.23 states per eV per f.u.). The hydrogen sublattice exhibits anisotropic properties, and is in a solid (not glassy, ​​like in *P*1‐SrH_22_^[^ ^30^ ^]^) state. Due to the presence of large amount of molecular hydrogen in the structure of SnH_14_, its Debye temperature is rather high (*θ*_D_ ≈1500 K, *ω*_log_ up to 1250 K). Also, the superconducting properties are well pronounced, the electron‐phonon coupling (EPC) coefficient is λ = 1.25, and the critical temperature is *T*_C_ = 107–133 K at 200 GPa (µ* = 0.15–0.1) in the harmonic approximation (Figures S12 and S13, Supporting Information). This significantly distinguishes molecular tin polyhydrides from similar compounds of barium (BaH_12_, which is a low‐*T*_C_ superconductor, *T*_C_ ≈20 K) and strontium (SrH_22_, which is a semiconductor).

In our last experiment performed in 2022 at APS, Sn, and AB were again used. The studies were carried out in DAC S3 with a wide opening angle (≈70°) and diamond anvils of the Böhler‐Almax type to obtain a single‐crystal XRD pattern (Figure S2, Supporting Information), where one phase was indexed by the *fcc* SnH_4_ (*R*‐factor is 4.75 %). A co‐product of the synthesis is a lower hydride, whose X‐ray diffraction pattern can be indexed by *C*2‐Sn_12_H_18_ (= Sn_2_H_3_, *R*_1_ = 5.83%, Figure S4, Supporting Information) with an experimental unit cell volume of 15.85 Å^3^/Sn at 192 GPa. This phase was found during the USPEX crystal structure search. Obtained Sn_12_H_18_ consists of [SnH_4_] structural blocks with *d*_Sn‐H_ = 1.87–1.97 Å (at 200 GPa) and multiple Sn─H─Sn hydrogen bonds.

Summarizing the structural studies of tin hydrides at high pressures, we can conclude that the Sn─H system is very rich in various compounds. Structural interpretation of most of these compounds is difficult because of the low enthalpy of formation of Sn hydrides. This does not allow reliable use of computational methods to predict thermodynamically stable phases. Obviously, the main product of the reaction of tin with hydrogen is *fcc* SnH_4_ (*V* = 19.6 Å^3^/Sn at 180 GPa). Among the higher polyhydrides *C*2/*m*‐SnH_14_ (*V* = 35.8 Å^3^/Sn at 193 GPa) and, possibly, *fcc* SnH_6±x_ (*V* = 24.1 Å^3^/Sn at 180 GPa, *x* = 0.5) were found. Among the lower hydrides *fcc* SnH_2+x_ (*V* = 17.0 Å^3^/Sn at 185 GPa, *x* 0.5) and *C*2‐Sn_12_H_18_ (*V* = 15.85 Å^3^/Sn at 192 GPa) were synthesized.

### 2.3 Transport Properties of fcc SnH_4_

**Figure 3:**
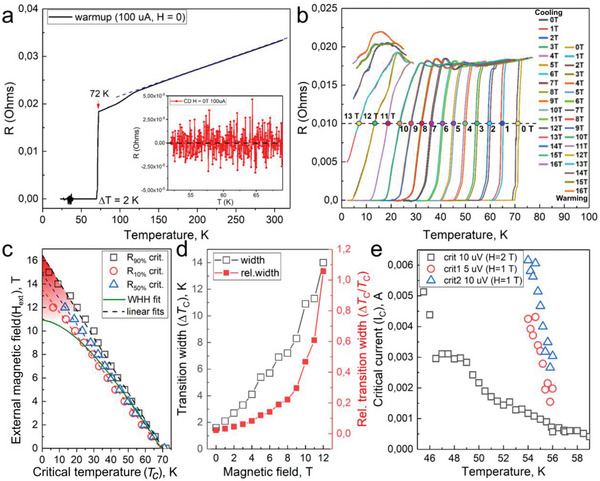
Electric transport properties of *fcc* SnH_4_ at 180 GPa studied in magnetic fields. a) Temperature dependence of the electrical resistance of the sample. Fully linear *R(T)* is observed from 300 to 120 K. At ≈30 K, the electrode system becomes unstable. Inset: residual resistance at *T* < *T*_C_. b) Displacement of the superconducting transition in external magnetic fields up to 16 Tin warming and cooling cycles. c) Experimental temperature dependence of *T_C_* (*H*_ext_). Using linear interpolation by different criteria (90, 50, and 10% of the normal state resistance) the values of upper critical magnetic field (*B*_C2_) were obtained. WHH fit is shown with a green curve. The area of deviation from this model is marked in red (see also Figure S24b, Supporting Information). d) Relative and absolute broadening of superconducting transitions in external magnetic fields. e) Temperature dependence of the critical current of the Sn hydride sample in external magnetic fields of 1 and 2 T.

Knowing the crystal structure of new hydride compounds, it is very important to experimentally study their physical properties. Therefore, we studied the transport properties of the obtained *fcc* SnH_4_ using a four‐electrode Van der Pauw circuit sputtered on diamond anvils. At 180 GPa, a sharp drop in the sample electrical resistance (by a factor of 10^3^) at *T*_C_ = 72 K with the width of Δ*T*_C_ = 2 K was revealed (**Figure** **3**a). The sample can therefore be characterized as homogeneous, which is also confirmed by the single crystal XRD data. It has been established that in external magnetic fields up to 16 T the value of the critical temperature *T*_C_ decreases almost linearly, which is typical for superconductors (Figure 3b,c). In the external magnetic fields (*H_ext_*) we observed a significant broadening of superconducting transitions in the range of 2 – 10 K (Figure 3d), which correlates with data of Ref. [25]. According to previous results,^[^ ^31^, ^32^, ^33^ ^]^ the superconducting transitions of some polyhydrides practically do not broaden in relatively weak magnetic fields (*µ*_*0*_ *H_ext_* < 0.5*B*_C2_(0)). Here, due to a much lower critical field *B*_C__2_(0) for SnH_4_ we were able to trace the *B*_C2_(T) dependence over almost the entire temperature range. As Figure 3b,d show, SnH_4_ manifests pronounced broadening of the superconducting transition in external fields.

All of the samples we examined in electrode DACs were superconducting at pressures above 170 GPa. Several SC transitions corresponding to different tin hydrides were observed in some DACs (see Figures S17 and S18, Supporting Information, *T*_C_’s ≈ 25 and 39 K). The highest *T*_C_ observed in the Sn‐H system in our experiments is 72–74 K at 180–190 GPa. When the pressure drops below 170 GPa, a sharp decrease in *T*_C_ to 20–25 K is found. The superconducting transition temperature in SnH_4_ increases with increasing pressure: *T*_C_ (180 GPa) = 72 K, *T*_C_ (190 GPa) = 74 K, *T*_C_ (≈200 GPa) = 75 K (warming cycle^[^ ^25^ ^]^). This corresponds to a positive slope d*T*_C_/dP = +0.1 – 0.2 K GPa^−1^, which is expectedly associated with acoustic phonons hardening (Section S7, Supporting Information).

Linear extrapolation of *B*_C2_(*T*) to *T*_C_ = 0 K allows the upper critical magnetic field of SnH_4_ to be estimated as *B*_C2_(0) = 14–16 T. This is a very low value compared to other hydride superconductors. For example, YH_6_^5^ has the *B*_C2_(0) value of at least 110–160 T, and for LaH_10_^2^ it is at least 140–160 T. One possible reason of that is the relatively low density of electronic states (Figure S11, Supporting Information), in particular, the low contribution of the hydrogen sublattice to the total density of electronic states of SnH_4_, which is critical for superconductivity in polyhydrides. Analogies can be found in studies of the cerium CeH_9‐10_^[^ ^7^ ^]^ and thorium ThH_9‐10_,^[^ ^4^ ^]^ superhydrides which despite higher *T*_C_ also exhibit relatively low *B*_C2_(0) ≈27–45 T.

From measurements of the temperature dependence of resistance, we focus first on a pronounced feature (kink) at ≈120 K (Figure 3a; Figures S17b and S18b, Supporting Information) which we observed in several experiments. Hong et al.^[^ ^25^ ^]^ also saw a similar kink in the *R(T)* dependence for SnH_4_. Such anomalous behavior of *R(T)* was previously revealed in many hydrides, for instance, in BaH_12_,^[^ ^34^ ^]^ H_3_S^[^ ^1^ ^]^ and LaH_10_,^[^ ^2^, ^35^ ^]^ and in cuprates.^[^ ^36^ ^]^ Additional examples are given in Figures S20 and S21 (Supporting Information). Second, electrical resistance of SnH_4_ is completely linearly dependent on temperature (*β* = *R_0_^−1^dR/dT* = 1.77 × 10^−3^ K^−1^) in the range from 120 to 320 K (see Figure 3a). Because of these two anomalies, the resistance of SnH_4_ in the normal (non‐superconductive) state can hardly be fitted with the Bloch‐Grüneisen model^[^ ^37^ ^]^ of phonon‐mediated scattering. Indeed, an attempt to use the Bloch‐Grüneisen fit^[^ ^37^ ^]^ leads to a unrealistically low Debye temperature (*θ*_D_) of ≈100 K, which is not typical for polyhydrides under high pressure. A power‐law interpolation *R(T)* = *R_0_* +*A*×*T^n^* (Figure S22, Supporting Information) commonly used to analyze the Fermi‐liquid behavior, also indicates significant deviations in the SnH_4_ properties from the behavior of ordinary metals.

The linear dependence of the upper critical field on temperature requires more detailed discussion. For superconductors described by the Bardeen‐Cooper‐Schrieffer (BCS) theory,^[^ ^38^ ^]^ the generally accepted model for the *B*_C2_ *(T)* dependence is the Werthamer–Helfand–Hohenberg (WHH) model, which predicts flattening of the *B*_C2_ *(T)* dependence at low temperatures.^[^ ^39^ ^]^ However, for many compressed polyhydrides (e.g., YH_4_, LaH_x_ at low pressure^[^ ^40^ ^]^) the *B*_C2_ *(T)* function is almost linear down to temperatures of 1–2 K. Such behavior sometimes may be explained by the presence of two superconducting gaps^[^ ^41^, ^42^, ^43^, ^44^ ^]^ (Figure S19, Supporting Information). Indeed, for a number of polyhydrides (*Fm* $\bar{3}$ *m*‐LaH_10_, *Fm* $\bar{3}$ *m*‐YH_10_, *P*6_3_ */mmc*‐YH_9_) the solution of the anisotropic Migdal‐Eliashberg equations indicates the presence of two superconducting gaps.^[^ ^45^, ^46^, ^47^ ^]^ However, this explanation is unsatisfactory, since two‐gaps superconductivity is not universal (e.g., *Im* $\bar{3}$ *m*‐CaH_6_^[^ ^48^ ^]^). Another possible explanation is related to the mesoscopic inhomogeneity of the sample, the presence of regions (“islands”) with different composition and hydrogen content and, consequently, with different *T*_C_ and *B*_C2_.^[^ ^32^, ^49^, ^50^ ^]^ As long as such “islands” of superconductivity are still bound via the Josephson effect, one can still detect the superconductivity of the sample. The proposed explanation agrees with the appearance of an ascending feature in *R(T)*, indicating a significant increase in resistance at low temperatures (Figure 3b), possibly, due to the complex and shunted trajectory of the electric current.

One of the distinguishing features of superconductors is the existence of a critical current density *J*_C_ *(T)*, when superconductivity gets destroyed and the electrical resistance of the material becomes nonzero. The low upper critical magnetic field of SnH_4_, as expected, leads to low values of the critical current of the sample: *I*_C_ = 6 mA at 55 K in a magnetic field of 1 T, that corresponds to *J*_C_(55 K, 1T) ≈ 200 A mm^−2^ for the sample with 1 µm thickness and 30 µm diameter (Figure 3e; Figure S17a, Supporting Information). However, if we consider not the maximum size of the sample, but the interelectrode distance, which for our sputtering mask is ≈7 µm, then the estimates of the critical density increase significantly (see below).

The critical current measurements can be used to estimate the superconducting gap in SnH_4_. Talantsev et al.,^[^ ^51^, ^52^ ^]^ proposed the following model for s‐wave superconductors

(1) $$J_{c}\;\left(T\right)=\frac{B_{c1}\left(T\right)}{\mu_{0}\lambda_{L}\left(T\right)}$$

where *J*_C_(*T*) is the critical current density at temperature *T* in the absence of the magnetic field, and *λ*_*L*_ *(T)* is the penetration depth, and *B*_C1_ *(T)* – is the lower critical magnetic field. Detailed equations for this model are provided in Equation S5, Supporting Information. Surprisingly, superconductivity in tin hydride is quite easily suppressed by electric current. Fit of experimental *J*_C_(*T*) data (Figure S17, Supporting Information) yields 2Δ(0) ≈ 21.6 meV and 2Δ(0)/*k*_B_ *T*_C_ = 3.6, in a reasonable agreement with the BCS value of 3.52. At the same time, according to the Talantsev‐Tallon model, the self‐field critical current density in SnH_4_ at 0 K can reach *J*_C_(0) = 8.8 kA mm^−2^. Thus, superconductivity in SnH_4_ probably has a conventional electron‐phonon mechanism.

**Figure 4:**
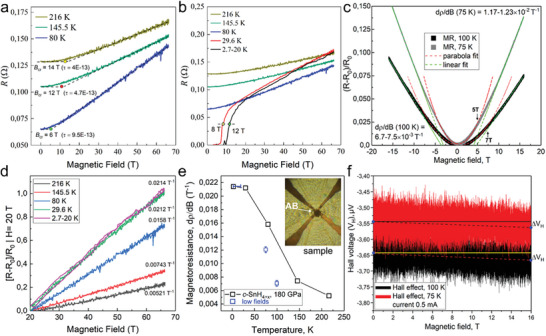
Magnetoresistance (MR) of the *fcc* SnH_4_ at 180 GPa was measured in pulse magnetic fields. a) Dependence of the electrical resistance on the applied magnetic field at temperatures *T* > *T*_C_. A pronounced transition from a quadratic to a linear *R(H_ext_)* dependence is observed. b) Dependence of the sample resistance on the applied magnetic field over the entire temperature range. Due to the heating by eddy currents during the pulse, the sample temperature of 2.7 K cannot be considered reliably fixed. c) MR in low magnetic fields up to 16 T at temperatures 75 and 100 K. d) Linear part of the relative MR calculated for all temperature points starting from a field *B_ext_* of 20 T. e) Dependence of linear MR on temperature. f) Attempt to measure the Hall effect in Sn hydrides at temperatures 75 and 100 K.

The results of studying the magnetoresistance (MR) of SnH_4_ at 180 GPa in pulsed magnetic fields up to 65 T revealed a curious result. The dependence of the electrical resistance *δR = (R‐R_0_*) ∝ *µ* ^2^ *B* ^2^ on magnetic field *B_ext_* (where *µ* is the mobility of carriers)^[^ ^53^ ^]^ is quadratic only in relatively weak magnetic fields. Then, rather quickly (when *B_ext_* > *B*_crit_) this dependence becomes linear *δR* ∝ (*B* – *B*_0_), see **Figure** **4**a,c. Taken separately, each of the two effects – linear *δR* *(T)* and linear *δR* *(B)* dependences may find analogies in other fields of physics. For example, linear *δR* *(T)* occurs in strongly interacting 2D systems of electrons^[^ ^54^ ^]^ where it originates from interaction assisted impurity scattering. Linear *R* *(B)* is known for polycrystalline samples (Kapitza linear magnetoresistance) and originates from scattering by the grain boundaries in quantizing magnetic fields,^[^ ^55^, ^56^, ^57^ ^]^ or is found in materials with Dirac cone spectrum. Mesoscopic disorder with several metal phases can also lead to such effect.^[^ ^58^ ^]^ However, the combination of the two effects in one and the same material leaves the most probable only one analogy – with high‐*T*_C_ cuprates which exhibit non‐Fermi‐liquid behavior in the normal state.^[^ ^59^, ^60^ ^]^ These so called strange metals are known to be materials between insulators and metals.^[^ ^61^ ^]^ Following the interpretation widely used for cuprate superconductors, we can say that the combination of the anomalous *T* and *B*‐linear behavior of electrical resistance and magnetoresistance, as well as the upper critical field of *fcc* SnH_4_ allows to characterize the tin tetrahydride in the normal state as a non‐Fermi‐liquid strange metal.

In general, the results of experiments in pulse magnetic fields agree with measurements in steady fields below 16 T: the MR of SnH_4_ only in the initial part (up to 5–7 T) is a quadratic function of the field. With a further increase in the field, MR becomes completely *B*‐linear. In addition, measurements in weak magnetic fields (0‐16 T, at 75–100 K, Figure 4c,f) allowed us only to estimate the Hall coefficient *R*_H_ ≈5 × 10^−12^ m^3^ C^−1^. The detected Hall voltage was unusually small ΔV_H_ ≈1–2×10^−8^ V at a current of 0.5 mA (Figure 4d). It is impossible to give a more accurate estimate, since we do not know the exact thickness (t) of the sample (t ≈1–2 µm). Nevertheless, this value of *R*_H_ is rather small which allows us to consider the concentration of charge carriers in SnH_4_ under pressure to be rather high 10^29^–10^30^ m^−3^ (as *R*_H_ = 1/n_e_e, the sign of the carrier charge is unknown). Alternatively, the low Hall coefficient may be due to high hole mobility, which can make a significant contribution to the conductivity and reduce the Hall coefficient. For instance, rough estimates of the mobility of electrons and holes in Sn_8_H_30_ at 180 GPa show that the mobility of holes in tin tetrahydride can be tens of times higher than the electron mobility (Table S14, Supporting Information).

Given the almost identical value of the Fermi velocity V_F_ = 2.5 × 10^5^ m s^−1^ in various hydride superconductors,^[^ ^62^ ^]^ we can estimate the mean free path of an electron in SnH_4_ in terms of the Drude theory:^[^ ^63^, ^64^ ^]^

(2) $$l_{e}=V_{F}\;\tau ≅\frac{m_{e}R_{H}}{\mathrm{eRt}}$$

where *R* is the electrical resistance of the sample. It can be seen that in the SnH_4_ the mean free path of electrons *l_e_* ≅10^−15^ *V_F_* =  3.5 Å, which is comparable to the size of unit cell. Of course, this is a very small value corresponded to extremely “dirty” and disordered samples. In a previous study of (La, Nd)H_10_^[^ ^32^ ^]^ we found that the electron mean free path is also very short (≈1.3 nm) and compatible with the unit cell parameter. This may lead to quantum effects of weak localization^[^ ^65^ ^]^ even in 3D samples of SnH_4_, since the corresponding correction (δσ) to the electrical conductivity (σ) may be significant (Equation 3).

(3) $$\frac{\delta \sigma }{\sigma }=-\frac{1}{k_{F}^{2}l_{e}}\left(\frac{1}{l_{e}}-\frac{1}{L_{\varphi }}\right)≅-1.$$

Weak localization effects are well known for semiconductors and graphene,^[^ ^66^ ^]^ in particular, they are responsible for the appearance of negative magnetoresistance in thin films of Si, Ge, and Te.^[^ ^67^ ^]^ A similar phenomenon was recently observed for (La,Nd)H_10_, which exhibits a negative magnetoresistance in the range of 200–250 K.^[^ ^32^ ^]^ The weak localization can also lead to an unusual *R* *(T)* dependence and sign reversal of *dR*/*dT*,^[^ ^68^ ^]^ as it was observed for lanthanum‐cerium (La,Ce)H_9_ and sulfur H_3_S hydrides (Figures S21c–22c, Supporting Information).

## 3 Discussion

Let us first discuss selection of the best theoretical structural model for *fcc* SnH_4_ in terms of the experimental superconducting properties. The two predicted structures, namely *R* $\bar{3}$ *m‐*Sn_12_H_45_ and *Fd* $\bar{3}$ *m*‐Sn_8_H_30_, are dynamically stable, well describe the experimental powder XRD patterns, and lie near the convex hull of Sn‐H system (Figure S1, Supporting Information). They both have electronic band structures (Figure S11, Supporting Information) typical of metals. The low density of electronic states at the Fermi level (≈0.5 states per eV per Sn) also has a relatively weak contribution from the hydrogen atoms (half the contribution of Sn), which is usually attributed to low critical temperature of superconductivity.^[^ ^69^, ^70^ ^]^ The calculated critical temperature for *Fd* $\bar{3}$ *m* modification (*T*_C_ = 73–91 K, Figure S25, Supporting Information) is close to the experimental one (72–74 K) observed in *fcc* SnH_4_ at 180 GPa. The results of single‐crystal XRD, which do not reveal any deviations from the ideal *fcc* structure, as well as high critical temperature of superconductivity, indicate that only *Fd* $\bar{3}$ *m*‐Sn_8_H_30_ theoretical model agrees well with the experimental data for *fcc* SnH_4_ at 180 GPa.

It is now believed^[^ ^1^ ^]^ that hydrides belong to the conventional BCS superconductors and their behavior in the normal state can be described in terms of the Fermi liquid model, widely used for metals. However, gradually accumulating experimental data cast doubt on this point of view. First of all, it is necessary to note the change in the sign of the temperature coefficient of electrical resistance (*dR/dT*) from positive to negative along with decreasing pressure in DACs. This was observed in superconducting H_3_S,^[^ ^1^ ^]^ PH_x_^[^ ^71^ ^]^ and lanthanum‐cerium polyhydrides^[^ ^72^ ^]^ (Figure S20, Supporting Information). The decrease in electrical resistance with increasing temperature is unusual for metals, but is a distinctive feature of insulators. Moreover, a similar behavior was recently observed in a cuprate superconductor Bi‐2212 (Bi_2_Sr_2_CaCu_2_O_8+_), studied at high pressures.^[^ ^73^ ^]^ This experiment demonstrated that pressure (*P*) may play the same role as the degree of doping (x),^[^ ^74^, ^75^ ^]^ separating the superconducting phase from the pseudogap phase with an insulating‐like negative *dR/dT*.

As many cuprates have a dome‐shaped dependence of the critical *T*_C_(x) on the degree of doping (x), so do most superhydrides have a similar *T*_C_(*P*) dependence with a sharp decrease in *T*_C_ with pressure decreasing.^[^ ^1^, ^2^, ^6^, ^7^ ^]^ Such decompression is usually accompanied by a sharp increase in the EPC parameter λ to the maximum within the Migdal‐Eliashberg theory^[^ ^76^, ^77^ ^]^ values of 3 – 3.7^[^ ^78^ ^]^ followed by decomposition of a compound. There is such a strong electron‐phonon interaction that even a small shift of atoms causes a large change in the electronic structure, turning the metal into an insulator with local separation of charges (as in ferroelectrics) and formation of polarons.^[^ ^79^ ^]^ What can happen to the atomic sublattice of hydrogen in superhydrides under these conditions? It would be logical to assume that hydrogen atoms will form more stable H_2_ molecules, and the compound will begin to exhibit the properties of a semiconductor, which is typical for molecular hydrides. That is exactly the boundary between the superconducting and insulating phases in polyhydrides.

The behavioral peculiarities of the electrical resistance of superhydrides have not received much attention since discovery of H_3_S, but many of them (e.g., Figures S20–S21, Supporting Information) show *R(T)* typical of strange metals in one pressure range, and the usual behavior of a Fermi liquid in another one. Amazingly, but the non‐superconducting state of polyhydrides may turn out to be much closer to the properties of cuprate superconductors than previously thought, as Talantsev et al.^[^ ^80^, ^81^, ^82^, ^83^ ^]^ predicted based on an analysis of the *T*_С_/*T*_F_ ratio (*T*_F_ is the Fermi temperature).

## 4 Conclusions

We have synthesized a series of novel tin hydrides at a pressure of 180–210 GPa: *C*2/*m*‐SnH_14_, *fcc* SnH_4_, *fcc* SnH_6+x_, *fcc* SnH_2_, and *C*2‐Sn_12_H_18_. The main product of the reaction of Sn with hydrogen at these pressures is the cubic (*fcc*) superconducting tetrahydride SnH_4_, with the composition being confirmed by XRD analysis performed after compression of cryogenically loaded stannane. The critical superconducting temperature of this compound is *T*_C_ = 72 K at 180 GPa, the upper critical field is *B*_C2_(0) = 14–16 T, the self‐field critical current density is *J*_C_(0) ≈8.8 kA mm^−2^, and the superconducting gap 2Δ(0) is 21.6 meV. SnH_4_ contains covalent Sn‐H bonds, has soft modes in phonon spectra and the large electron‐phonon coupling parameter λ ≈2.5 due to important contribution of anharmonicity to the crystal lattice dynamics.

Another discovered polyhydride *C*2/*m*‐SnH_14_ with ultra‐high hydrogen content, belongs to the same family of compounds as BaH_12_,^[^ ^34^ ^]^ LaH_11‐12_,^[^ ^84^ ^]^ SrH_22_,^[^ ^30^ ^]^ and confirms the existence and prevalence of such a type of high molecular polyhydrides. Unlike other members of this group, SnH_14_ is expected to be a high‐temperature superconductor with *T*_C_ = 93–112 K.

Tin tetrahydride (SnH_4_) exhibits a reproducible anomalous dependence of electrical resistance on temperature, *T*‐linear dependence of the upper critical field, and a *B*‐linear dependence of magnetoresistance over a wide range of magnetic fields and temperatures. Such a non‐Fermi‐liquid behavior is reminiscent to the unconventional non‐superconducting state of cuprate high‐temperature superconductors.

## Conflict of Interest

The authors declare no conflict of interest.

## Author Contributions

I.A.T., D.V.S., and A.G.I. contributed equally to this work. I.A.T., D.V.S., D.Z., T.H., and S.W.T. performed the experiments. I.A.K. performed the T‐USPEX and anharmonic phonon density of states calculations. A.G.K., D.V.S., and D.Z. prepared the theoretical analysis and calculated the equation of states, electron and phonon band structures, and superconducting properties of tin hydrides. D.V.S. and D.Z. analyzed and interpreted the experimental results and wrote the manuscript. I.A.T., A.V.S., and O.A.S. made the electric transport measurements in low magnetic fields. M.V.L. analyzed Raman experiments and edited the manuscript. D.S.P. built a system for the synthesis and cryogenic loading of stannane. T.H. and S.W.T. carried out the measurements in pulsed magnetic fields at HZDR HLD. I.S.L. and V.M.P. directed the research, analyzed the results and edited the manuscript. All the authors provided critical feedback and helped shape the research.

## Supporting information

Supporting Information

## Data Availability

The data that support the findings of this study are available in the supplementary material of this article.
