# Supplementary material for: Non‐Fermi‐Liquid Behavior of Superconducting SnH4
Source: Adv Sci (Weinh). 2023 Aug 25;10(30):2303622. doi: 10.1002/advs.202303622 (PMC10602579; doi:10.1002/advs.202303622)
Supplement: Supplementary file 1 — Supporting Information [file ADVS-10-2303622-s001.pdf]

ADVANCED SCIENCE
Open Access

Supporting Information

for *Adv. Sci.*, DOI 10.1002/advs.202303622

Non-Fermi-Liquid Behavior of Superconducting SnH_4

Ivan A. Troyan, Dmitrii V. Semenok, Anna G. Ivanova, Andrey V. Sadakov, Di Zhou*, Alexander G. Kvashnin, Ivan A. Kruglov, Oleg A. Sobolevskiy, Marianna V. Lyubutina, Dmitry S. Perekalin, Toni Helm, Stanley W. Tozer, Maxim Bykov, Alexander F. Goncharov, Vladimir M. Pudalov and Igor S. Lyubutin**

((Supporting Information can be included here using this template))

Supporting Information

Non-Fermi-Liquid Behavior of Superconducting SnH_4

*Ivan A. Troyan,[1] Dmitrii V. Semenok,[2,] * Anna G. Ivanova,[1] Andrey V. Sadakov,[3] Di Zhou,[2,] * Alexander G. Kvashnin,[4] Ivan A. Kruglov,[5,6] Oleg A. Sobolevskiy,[3] Marianna V. Lyubutina,[1] Dmitry S. Perekalin,[7] Toni Helm,[8] Stanley W. Tozer,[9] Maxim Bykov,[10] Alexander F. Goncharov,[11] Vladimir M. Pudalov,[3,12] and Igor S. Lyubutin[1,] **

Contents

1. Methods 2
2. Crystal structure search 6
3. X-ray diffraction and unit cell parameters of tin hydrides 8
4. Raman spectra 25
5. Electron and phonon band structures of tin hydrides 27
6. Transport and superconducting properties 29
7. Eliashberg functions 36
8. Elastic properties 39
References 40

1. Methods

1.1. Theory

The computational predictions of thermodynamic stability of the Sn–H phases at high pressures of 180 and 200 GPa were carried out using the variable-composition evolutionary algorithm USPEX [1-3]. The first generation, consisting of 80 structures, was produced using the random symmetric [3] and random topology [4] generators, whereas all subsequent generations contained 20% of random structures and 80% of structures created using heredity, softmutation, and transmutation operators.

The evolutionary searches were combined with structure relaxations using the density functional theory (DFT) [5,6] within the Perdew–Burke–Ernzerhof (PBE) generalized gradient approximation (GGA) functional [7] and the projector augmented wave method [8,9] as implemented in the VASP code [10-12]. The kinetic energy cutoff for plane waves was 600 eV. The Brillouin zone was sampled using *Γ*-centered *k*-points meshes with a resolution of $2\pi \times 0.05$ Å^{-1}. The same parameters were used to calculate the equations of state of the discovered phases. We also calculated the phonon densities of states of the studied materials using the finite displacements method (VASP and PHONOPY [13,14]). This methodology is similar to that used in our previous works [15,16].

The calculations of the critical temperature of superconductivity T_C were carried out using Quantum ESPRESSO (QE) package [17,18]. We used the tetrahedron method with an offset q-point grid in order to avoid the singularity at $q = \Gamma$. The phonon frequencies and electron–phonon coupling (EPC) coefficients were computed using the density functional perturbation theory [19], employing the plane-wave pseudopotential method and the PBE exchange–correlation functional. The critical temperature (T_C) was calculated using the Allen–Dynes formula [20].

The dynamic stability and phonon density of states of Sn_8H_{30}, $Sn_{12}H_{45}$ and SnH_{14} were studied using classical molecular dynamics and interatomic potentials (MTP) based on machine learning [21]. It was demonstrated that the MTP can be used to calculate the phonon properties of materials [22]. Moreover, within this approach we can explicitly take into account the anharmonicity of hydrogen vibrations.

To train the potential, we first simulated two models of cubic SnH_4, Sn_8H_{30} and $Sn_{12}H_{45}$, as well as SnH_{14} in quantum molecular dynamics in an NPT ensemble at a temperature of 2000 K, with a duration of 5 picoseconds using the VASP code [10-12]. We used the PAW PBE pseudopotentials for the H and Sn atoms, $2\pi \times 0.06$ Å^{-1} *k*-mesh with a cutoff energy of 400 eV. For training of the MTP, a set of structures of tin hydrides was chosen using active learning [23]. We checked the dynamic stability of Sn_8H_{30}, $Sn_{12}H_{45}$ and SnH_{14} using the obtained MTPs via several runs of molecular dynamics calculations at 300 K. First, the NPT dynamics simulations were performed in a supercell with about 1000 atoms for 40 picoseconds. During the last 20 picoseconds, the cell parameters were averaged. At the second step, the coordinates of the atoms were averaged within the NVT dynamics with a duration of 20 picoseconds and the final structure was symmetrized.

Then, the phonon density of states (DOS) was calculated within the MTP using the velocity autocorrelator (VACF) separately for each type of atoms [24]:

$$g(\vartheta) = 4\int_0^\infty \cos(2\pi\vartheta t)\frac{\langle\overline{\vartheta(0)\vartheta(t)}\rangle}{\langle\overline{\vartheta(0)}^2\rangle}dt, \quad \text{(S1)}$$

where ϑ is the frequency. The velocity autocorrelator was calculated using molecular dynamics, then the phonon DOS was obtained.

The critical temperature of superconductivity (T_C) was estimated using the Allen-Dynes formula

$$T_c = \omega_{log}\frac{f_1 f_2}{1.2}\exp\left(\frac{-1.04(1+\lambda)}{\lambda - \mu^* - 0.62\lambda\mu^*}\right), \quad \text{(S2)}$$

where product of Allen-Dynes coefficients f_1 and f_2 is

$$f_1 f_2 = \sqrt[3]{1 + \left(\frac{\lambda}{2.46(1+3.8\mu^*)}\right)^{\frac{3}{2}}} \cdot \left(1 - \frac{\lambda^2(1-\omega_2/\omega_{log})}{\lambda^2 + 3.312(1+6.3\mu^*)^2}\right). \quad \text{(S3)}$$

Polyhydrides at high pressure can be considered as binary alloys of metallic hydrogen and other elements, for example, metals. As demonstrated by J. Appel [25] for binary disordered alloys, the electron-phonon interaction coefficient in this case can be expressed as

$$\lambda = -\sum_{\alpha,\beta}\frac{N_\alpha(0)N_\beta(0)}{N(0)}\cdot\frac{\langle V\rangle_{\alpha\beta}}{p_\alpha p_\beta}, \quad \text{(S4)}$$

where $N_\alpha(0)$ and $N_\beta(0)$ are the partial contributions of metal (α) and hydrogen (β) atoms to the density of electronic states at the Fermi level (DOS). p_α and p_β are the probabilities of occupying certain positions by these atoms, and <V> are the averaged matrix elements of the electron-phonon interaction.

It should be taken into account that the strength of the electron-phonon interaction is the main driver of the critical temperature of superconductivity. Considering that $N_\alpha(0)$ = x $N(0)$, $N_\beta(0)$ = (1- x) $N(0)$, we can conclude that the highest λ achieved at x = 0.5 as maximum of x(1-x) function. This corresponds to results of many ab initio calculations: the contribution of hydrogen to the density of electronic states of a superhydride should be approximately 50% of the total density of states. If the contribution of hydrogen to $N(0)$ is small (x << 0.5), we come to superconductivity in metals, which even at high pressure does not exceed T_C ~ 30 K. If the contribution of hydrogen is large (x ~ 1) – this is the case of molecular polyhydrides (such as BaH_{12}, SrH_{22}), in which isolated hydrogen molecules weakly interact with each other.

There are several techniques to probe order parameter in superconductors. Some of them are based on tunnel effects – Josephson effect and Andreev reflection effects, while others are based on the investigating of the temperature dependence of the superfluid density (ρ_s). The latter proved to be a reliable tool to determine not only the quantity and values of energy gaps, but also the symmetry of order parameter – whether it has s-wave, d-wave or even more complex structure [26]. Temperature dependence ρ_s(T) is usually deduced by means of either measuring directly the London penetration depth λ(T), or measuring of lower critical field, or measuring self-field critical current density J_C(T). Given all the limits and difficulties of DAC measurements we were only able to measure the self-field critical current. According to Ref. [26,27] the temperature dependence of J_C(T) is related to the penetration depth λ(T) by formulas

$$J_c(T) = \frac{\hbar}{4e\mu_0\lambda^3(T)}(\ln(\kappa) + 0.5) \cdot \left(\frac{\lambda(T)}{a}\tanh\left(\frac{a}{\lambda(T)}\right) + \frac{\lambda(T)}{b}\tanh\left(\frac{b}{\lambda(T)}\right)\right),$$

$$\frac{\lambda(T)}{\lambda(0)} = \sqrt{1 - \frac{1}{2k_BT}\int_0^\infty cosh^{-2}\left(\frac{\sqrt{\varepsilon^2 + \Delta^2(T)}}{2k_BT}\right)}, \qquad \text{(S5)}$$

$$\Delta(T) = \Delta(0) \cdot \tanh\left(\frac{\pi k_BT}{\Delta(0)}\sqrt{\eta\left(\frac{\Delta C}{C}\right)\left(\frac{T_C}{T} - 1\right)}\right),$$

where $2a$ – is the width of sample, $2b$ – is the thickness of sample, μ_0 is the permeability of free space, e is the electron charge, $\kappa = \lambda/\xi$ is the Ginsburg-Landau parameter, $\Delta(T)$ – the superconducting gap, $\eta = 2/3$ for s-wave superconductivity, and $\Delta C/C$ – is the specific heat capacity jump at the superconducting transition. In these equations, parameters b, $\Delta(0)$, $\lambda(0)$ and $\Delta C/C$ are refined parameters. The best fit [28] is shown in Supporting Figure S17d.

1.2. Experiment

We used the diamond anvils with a 50 μm culet beveled to 300 μm at 8.5°, equipped with four ~200 nm thick Ta electrodes with ~80 nm gold plating that were sputtered onto the piston diamond. Rhenium or steel ring composite gaskets were used for electrical measurements, and a mixture of CaF_2/epoxy resin was used to insulate the gaskets from electrical leads. Sn pieces with a thickness of ~1–2 μm were sandwiched between the electrodes and ammonia borane NH_3BH_3 (AB) layer. Ammonia borane served as a pressure-transmitting medium and simultaneously was a source of hydrogen at the laser heating of the sample. The laser heating of the samples above 1500 K at pressures of 170–180 GPa was done by several 100 μs pulses and led to the formation of various Sn hydrides, whose structure was analyzed using the single-crystal (SCXRD [29-31]) and powder X-ray diffraction (XRD). It should be noted that DACs used for XRD have a wide opening angle (60-80°), while DACs prepared for the transport studies cannot be used for XRD due to presence of Ta/Au electrodes and small opening angle.

The XRD patterns of tin hydrides were studies at the ID27, P02 and HPCAT beamlines of ESRF (Grenoble, France), PETRA III (Hamburg, Germany) and APS (Argonne, USA) respectively, using monochromatic synchrotron radiation and an imaging plate detector at room temperature. The X-ray beam was focused in a less than 3 × 3 μm spot. The XRD data were analyzed and integrated using Dioptas software package (version 0.5) [32]. The full profile analysis of the diffraction patterns and the calculation of the unit cell parameters were performed using JANA2006 software [33] with the Le Bail method [34]. The pressure in the DACs was determined via the Raman signal from diamond at room temperature [35]. We should note that pressure in all DACs changes during cooling from 300 K to 4 K by about 5-10 GPa.

Single crystal XRD was performed at the beamline 13 IDD (GSECARS, Advanced Photon Source, Argonne, USA). For the single-crystal XRD measurements samples were rotated around a vertical ω-axis in a range ±32°. The diffraction images were collected with an angular step Δω = 0.5° and an exposure time of 5-10 s/frame. For analysis of the single-crystal diffraction data (indexing, data integration, frame scaling and absorption correction) we used

the CrysAlisPro software package, version 1.171.42.54a. To calibrate an instrumental model in the CrysAlisPro software, i.e., the sample-to-detector distance, detector's origin, offsets of goniometer angles, and rotation of both X-ray beam and the detector around the instrument axis, we used a single crystal of orthoenstatite (($Mg_{1.93}Fe_{0.06}$)($Si_{1.93}$, $Al_{0.06}$)O_6, *Pbca* space group, a = 8.8117(2), b = 5.18320(10), and c = 18.2391(3) Å).

Magnetoresistance measurements under high magnetic fields were carried out in a 24 mm bore 72 T resistive pulse magnet (rise time of 15 ms, full pulse time is 150 ms) at the Helmholtz-Zentrum Dresden-Rossendorf (HZDR). Non-magnetic materials were used to create the high-pressure diamond anvil cell for this experiment, in particular, we used the NiCrAl non-magnetic alloy, which has quite high resistivity. Strands of the Litz wire glued to the silver paint were moved closer together to minimize open loop pickup. The DAC was encased with strips of 125 µm × 1 cm wide Kapton tape. All twisted pairs were fixed using the GE7031 varnish (Lake Shore, 50:50 toluene: methanol thinner). A flow He cryostat (VTI) was used, which made it possible to better control the DACs temperature. Cernox thermometers (Lake Shore Cernox X95809) were attached to the DAC body for measurements of the temperature. There was no observable heating from the ramping of the magnetic fieldф at rates up to 100 T/sec at temperatures above 20 K. The relatively long pulse duration (150 ms) leads to an insignificant influence of eddy currents on the position and temperature of the sample, which turns out to be rather rigidly fixed in the cryostat, since the cryostat diameter differs from the DACs diameter only by 0.1-0.15 mm. Due to the fact that the SnH_4 sample is not in direct contact with the DACs body and the gasket, the temperature jump on the sample during the pulse due to induction heating is within 3-5 K.

A high-frequency (3.33 and 33.3 kHz) lock-in amplifier technique was employed to measure the sample resistance in a 72 T pulse magnet. The magnet can be used on very special occasions to 72 T, but is usually used to 65 T to extend its lifetime. For the measurements in high magnetic fields, we used a four-wire AC method with the excitation current of 0.5–1 mA [36]; the voltage drop across the sample was amplified by an instrumentation amplifier and detected by a lock-in amplifier. Comparing the up and down sweep resistance curves at various field sweep rates, no significant sample heating was observed during a ~150 ms long magnet pulse at temperatures above 35 K. In general, we used the same methodology as in the previous study of (La, Nd)H_{10} [37].

Table S1. List of high-pressure DACs used in the XRD experiments, samples loaded into them and products obtained. We used electrodeless DACs with a wide opening angle.

DAC	Experiment	Sample	Products
DAC S1	ESRF-2017	Sn	*hcp*-Sn, *bcc*-Sn
DAC S2	ESRF-2017	SnH_4, cryogenically loaded stannane	*fcc*-SnH_4, *hcp*-Sn
DAC D2	PETRA-2020	Sn/NH_3BH_3	*bcc*-Sn, *hcp*-Sn, *fcc*-SnH_4, *C*2/*m*-SnH_{14}
DAC M2	PETRA-2020	Sn/NH_3BH_3	*C*2/*m*-SnH_{14}, *fcc*-SnH_{6-8}, *fcc*-SnH_4, *fcc*-SnH_2
DAC S3	APS-2022	Sn/NH_3BH_3	*C*2-$Sn_{12}H_{18}$, *fcc*-SnH_4

2. Crystal structure search

It should be noted that the formation of tin polyhydrides at high pressures is a process with just a little thermodynamic benefit. This is evidenced by the values of the enthalpy of formation of Sn hydrides, which are ΔH ~ 10–60 meV/atom [38], whereas, those of other metal polyhydrides ΔH ($X + mH_2 \rightarrow XH_{2m}$) are of the order of hundreds meV/atom [38]. In addition, the low enthalpy of formation makes the chemical process more dependent on hyperfine effects such as the zero-point energy (ZPE), entropy factor, spin-orbit coupling (SOC), anharmonicity of the hydrogen sublattice, as well as the choice of pseudopotentials and exchange-correlation functional for DFT calculations. As a result, the real convex hull of the Sn-H system is difficult to predict from the first principles. This explains the failure of all previous theoretical studies of tin polyhydride formation at high pressure [38,39].

To verify that common DFT approaches are poorly suited to predict the existence of stable tin hydrides, we performed a search for the most appropriate crystal structure for SnH_4 composition using USPEX code [1-3,40] at 180 and 200 GPa (Supporting Figure S1). As a result, the *P*6/*mmm*, *P*6_3/*mmc* and *I4/mmm* phases of SnH_4 were identified as the most thermodynamically favorable. However, no cubic tin tetrahydrides were found. Moreover, none of the theoretically proposed Sn polyhydrides can explain the diffraction pattern of the material obtained by compressing SnH_4 and indexed as a cubic *fcc* lattice (experiment ESRF-2017). However, if we assume that the stoichiometric ratio Sn:H is not 1:4, but 1:3.75 (Th_4H_{15}-like structure), which may be the result of a decomposition reaction,

$$8\ SnH_4 \rightarrow Sn_8H_{30} + H_2, \qquad \text{(S6)}$$

then we can find a good approximation to the structural solution of the problem. The stoichiometry of Sn_4H_{15} allows for dynamically and thermodynamically stable cubic (*fcc*) metal lattices. The results of an evolutionary structural search among such compositions (up to supercells of $Sn_{32}H_{120}$) at 180 GPa are: $R\bar{3}m$-$Sn_{12}H_{45}$ (distorted $Fd\bar{3}m$) and $Fd\bar{3}m$-Sn_8H_{30} (Supporting Table S2, Figure 1c in the main text). Both structures are metals with unit cell volumes of about 19.7 Å^3/atom at 180 GPa. They are dynamically stable if one considers anharmonic effects. Both of these phases lie close to the convex hull of Sn-H system and exhibit *fcc*-like XRD patterns. Generally speaking, the most stable tin hydride at pressures of 170-210 GPa is *P*6_3/*mmc*-SnH_4 (with and without SOC, ZPE and entropy contribution, Supporting Tables S3, S4). But since such a compound was not observed among the experimental diffraction patterns, we excluded this phase from the consideration. Then, the most thermodynamically stable phase (with accounting ZPE) is $Fd\bar{3}m$-Sn_8H_{30} at 180 GPa, but $R\bar{3}m$-$Sn_{12}H_{45}$ at 200 GPa and 0 K. At 1000 K, the situation does not change: the cubic modification of $Fd\bar{3}m$-Sn_8H_{30} is still slightly above the convex hull at 200 GPa (Supporting Table S4). As shown below, the calculated parameters of the superconducting state of $Fd\bar{3}m$-Sn_8H_{30} are close to the experimental data.

Table S2. Calculated convex hull parameters of Sn-H system at 180 GPa and 0 K. E_{total} is the total DFT energy,
E_{form} is the energy of formation, X = H/(Sn+H).

Phase	Number of Sn atoms	Number of H atoms	X	E_{total}, eV	E_{form}, eV/atom
$P6_3/mmc$-Sn	2	0	0	30.982	0
$Fd\bar{3}m$- Sn_8H_{30}	8	30	0.789	116.208	-0.043
$R\bar{3}m$- $Sn_{12}H_{45}$	12	45	0.789	174.352	-0.042
$I\bar{4}m2$-SnH_8	2	16	0.889	27.088	-0.036
$C2/m$-SnH_{12}	2	24	0.923	25.686	-0.016
$C2/m$-SnH_{14}	2	28	0.933	25.219	-0.002
$C2/m$-H	0	24	1	-4.879	0

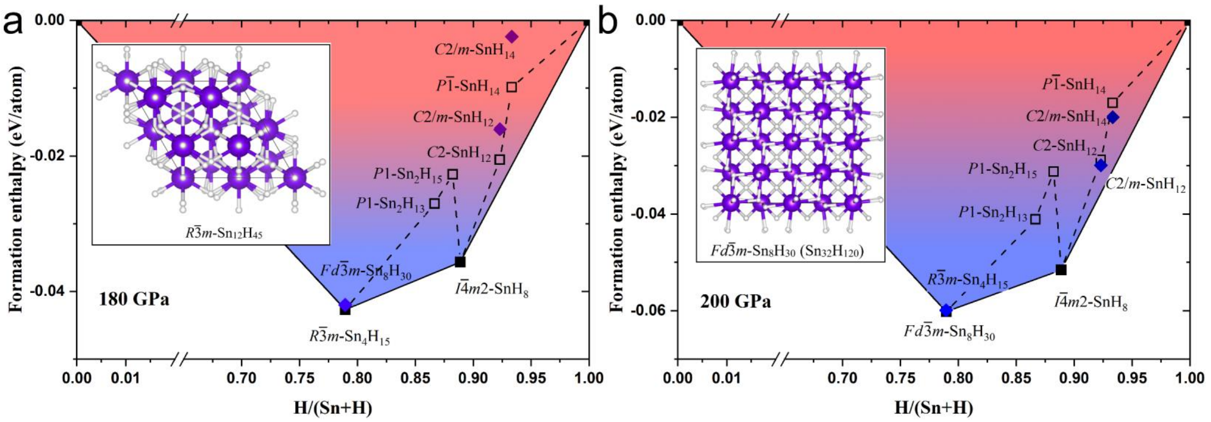

Figure S1. Convex hull of the Sn-H system calculated for pressures of 180 and 200 GPa and T = 0 K using the USPEX code without considering the ZPE. The most stable phase $P6_3/mmc$-SnH_4 is excluded. The insets show the structures of $R\bar{3}m$-$Sn_{12}H_{45}$ and $Fd\bar{3}m$-Sn_8H_{30} in the pressure range of 180–200 GPa. Consideration of ZPE and temperature effects has a little effect on the convex hull.

Table S3. Calculated convex hull parameters of Sn-H system at 200 GPa and 0 K. H_{form} is the DFT enthalpy of formation. ZPE is the zero-point energy in the harmonic approximation.

Phase	Sn atoms	H atoms	X	E(total), eV	E, eV/atom	E_{form}, eV/atom	ZPE, eV	E+ZPE, eV	H_{form}, eV/atom
$P6_3/mmc$-Sn	2	0	0	34.366	17.183	0	0.051	34.417	0
$Fd\bar{3}m$-Sn_8H_{30}	8	30	0.789	135.678	3.570	-0.060	8.556	144.234	-0.064
$R\bar{3}m$-$Sn_{12}H_{45}$	12	45	0.789	203.533	3.571	-0.060	12.332	215.865	-0.072
$P6_3/mmc$-SnH_4	2	8	0.800	33.450	3.345	-0.105	2.320	35.770	-0.104
$I\bar{4}m2$-SnH_8	2	16	0.889	33.705	1.872	-0.052	4.034	37.738	-0.082
$C2/m$-SnH_{12}	2	24	0.923	33.987	1.307	-0.030	6.750	40.738	-0.034
$C2/m$-SnH_{14}	2	28	0.933	34.321	1.144	-0.017	7.847	42.168	-0.021
$C2/m$-H	0	24	1	0.400	0.017	0	6.794	7.194	0

Table S4. Extended convex hull parameters of Sn-H system at 200 GPa and 300-1000 K. F_{vib} is the vibrational energy, G_{form} is the Gibbs free energy of formation.

Phase	F_{vib}, eV/atom			G_{form}, eV/atom		
Temperature	300 K	500 K	1000 K	300 K	500 K	1000 K
$P6_3/mmc$-Sn	-0.164	-0.379	-1.076	0	0	0
$Fd\bar{3}m$- Sn_8H_{30}	-0.485	-1.295	-4.849	-0.058	-0.049	-0.026
$R\bar{3}m$- $Sn_{12}H_{45}$	-0.712	-1.995	-7.648	-0.066	-0.059	-0.041
$P6_3/mmc$-SnH_4	-0.098	-0.284	-1.150	-0.097	-0.086	-0.059
$I\bar{4}m2$-SnH_8	-0.105	-0.342	-1.577	-0.077	-0.070	-0.051
$C2/m$-SnH_{12}	-0.134	-0.440	-2.118	-0.031	-0.025	-0.013
$C2/m$-SnH_{14}	-0.154	-0.512	-2.494	-0.020	-0.015	-0.007
$C2/m$-H	-0.038	-0.273	-1.577	0	0	0

Thus, in this section, we showed a vivid example of how far the results of theoretical DFT calculations can differ from the results of experiments at high pressure. Although we succeeded to find a satisfactory dynamically stable model (Sn_8H_{30}) to describe the properties of experimental cubic SnH_4, we have failed to solve the problem of thermodynamic stability of Sn polyhydrides at high pressure (Supporting Figure S1). Strictly speaking, at a given crystal structure $P6_3/mmc$-SnH_4, all experimentally found Sn hydrides appear to be displaced from the convex hull at 180-200 GPa and, in terms of the current level of theoretical structural search, can exist only as metastable phases. Obviously, there must be some unusual effect that stabilizes the cubic modification of SnH_4, but this problem is beyond the scope of this study.

3. X-ray diffraction and unit cell parameters of tin hydrides

Reaction products (APS-2022 experiment, Table S4) contained multiple good-quality single-crystalline domains of novel phases (Supporting Figure S2) and it was possible to find orientation matrices of several single crystals following well established procedures (see Ref. [41] for more details on the procedure).

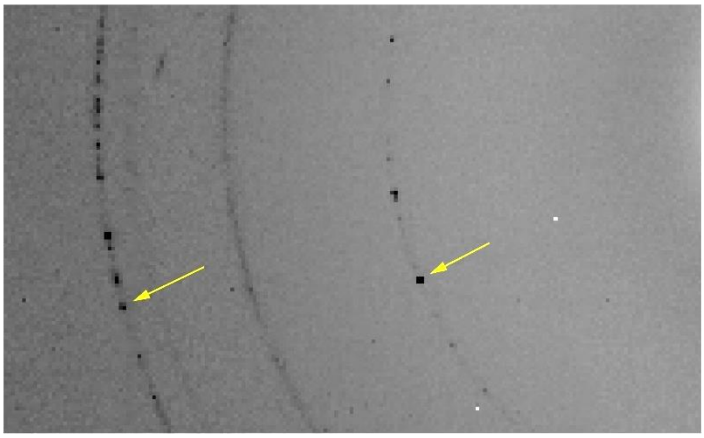

Figure S2. Example of the diffraction pattern showing reflections originating from small single crystals, APS-2022. These peaks are distinct and can be separated from the “bad powder” rings.

The dominant phase was initially indexed using a cubic *F*-centered lattice with *a* = 5.780(5) Å at 171 GPa. This lattice at the first glance does not correspond to any of the theoretically predicted Sn-H phases. Nevertheless, one should take into account that XRD gives

information on the positions of heavy atoms only due to the great difference in the atomic scattering factors of Sn and H. Therefore, it is not excluded that detection of weak superlattice reflections is beyond the current experimental capabilities. Unconstrained refinement of the unit cell parameters based on 42 single-crystal reflections revealed slight deviation of the cubic lattice parameters from each other: a = 5.780(5) Å, b = 5.796(10) Å, c = 5.775(9) Å, which might indicate that the lattice is indeed distorted.

A sequence of lattice transformations defined by the following matrices allows to convert the F-centered lattice to a monoclinic C-centered lattice and refine unit cell with a better final figure of merit:

$$cF \rightarrow mI \begin{pmatrix} 0.5 & 0 & 0.5 \\ -0.5 & 0 & 0.5 \\ 0 & -1 & 0 \end{pmatrix}; \quad mI \rightarrow mC \begin{pmatrix} -1 & 0 & -1 \\ 0 & 1 & 0 \\ 1 & 0 & 0 \end{pmatrix}; \quad cF \rightarrow mC \begin{pmatrix} -0.5 & 1 & -0.5 \\ -0.5 & 0 & 0.5 \\ 0.5 & 0 & 0.5 \end{pmatrix}$$

The C-centered lattice obtained by the transformation of F-centered cubic lattice agrees well with the predicted $C2$-$Sn_{12}H_{18}$ phase with a = 7.083(15) Å, b = 4.082 Å, c = 4.090 Å, β = 125.1°. Structure refinement in a cubic $Fm\bar{3}m$ symmetry (a = 5.780(5) Å) with Sn occupying Wyckoff sites 4b and 8c converges with an excellent agreement factor R_1 = 5.83%, confirming the pseudocubic arrangement of Sn atoms in the calculated $C2$-$Sn_{12}H_{18}$ structure (Table S4, Figure S3).

The second phase (*fcc* SnH_4) in the sample at 171 GPa was initially indexed with a cubic F-centered lattice with a = 4.244(3) Å. Crystal structure refinement suggests *fcc* arrangement of Sn atoms with a space group $Fm\bar{3}m$ and Sn occupying Wyckoff site 4a (R_1 = 4.75 %). This lattice parameter is exactly half of that of the predicted $Fd\bar{3}m$-Sn_8H_{30} phase. However, due to the low atomic scattering factor of hydrogen, the supercell could not be confirmed by single-crystal XRD.

Impurity reflections in the XRD patterns (Figure 2 in the main text) can be explained by orthorhombically distorted $Im\bar{3}m$ structure. However, as can be seen from the data of Supporting Tables S5, S7, S8, the resulting volume V ~ 23.5-24.1 Å^3 (Z = 2) is too small for *bcc*-Sn. Better agreement with the unit cell volume was obtained for $P6_3/mmc$-Sn structure (for example, a = 2.633 Å, c = 4.581 Å, V = 27.5 Å^3, Z = 2), but the refinement quality is lower. This indicates that the strong reflection at 2θ ≈ 8° (Figures 2a, d) may have a source other than residual tin.

In our experiment with DAC M2, in addition to SnH_{14}, several other tin hydrides were also detected. They can be indexed as two cubic phases: *fcc*-1 (Supporting Figure S5), and *fcc*-2 with a larger unit cell volume. The latter phase can also be indexed as pseudocubic $I4/mmm$ (Supporting Figure S6). For the *fcc*-1 phase, indexing gives the unit cell volume $V \approx$ 16.5-17.1 Å^3/Sn at 180 GPa, which is approximately 3 Å^3/Sn less than it is necessary for the SnH_4 composition. Such a value of the unit cell volume better matches the composition of SnH_2 where Sn has a formal valence of +2. For the second cubic phase *fcc*-2, refinement gives the unit cell volume V = 23.5-24.1 Å^3/Sn (Supporting Table S5), which corresponds to the composition SnH_{6+x} (x = 0 - 0.5, see Figure 2e), or the previously predicted stable tetragonal SnH_{8-x} [38].

Table S5. Summary table of the XRD experiments (without SnH_{14} and Sn). Unit cell parameters of $Fd\bar{3}m$-Sn_8H_{30} and $C2$-$Sn_{12}H_{18}$ compounds obtained in three experiments at different pressures are shown. Unit cell parameters of *fcc*-1 phase (SnH_2), *fcc*-2 (SnH_6 or SnH_{8-x}) and a gasket (e.g., *bcc*-Fe (?), one diffusive diffraction ring), obtained at PETRA-2020, are also shown. The experimental error in the pressure value is within 5 GPa.

**Phase**	**$Fd\bar{3}m$-Sn_8H_{30}**			**$C2$-$Sn_{12}H_{18}$ (β = 124.46°, fixed)**				***bcc*-Sn**	
**Experiment**	***a*, Å (Z = 32)**	***V*, Å^3 (Z = 32)**	***V*, Å^3/Sn**	***a*, Å**	***b*, Å**	***c*, Å**	***V*, Å^3/Sn**	***a*, Å**	***V*, Å^3 (Z = 2)**
ESRF-2017, 180 GPa<br>PETRA-2020, 170 GPa	8.567<br>8.619	628.7<br>640.5	19.64<br>20.0	-	-	-	-	2.996<br>3.012	26.9<br>27.32
APS-2022, 192-196 GPa	8.5075	615.7	19.24	7.007	4.095	4.020	15.85	-	-
APS-2022, 165-171 GPa	8.689	656.0	20.5	7.097	4.175	4.130	16.82	-	-
APS-2022, 150-155 GPa	8.719	662.8	21.2	7.087	4.205	4.100	16.85		-
**Phase**	***fcc*-1 SnH_2**			***fcc*-2, SnH_6 or pseudocubic *I4/mmm*-SnH_{8-x}**				***bcc* gasket (Fe ?)**	
PETRA-2020	***a*, Å (Z = 32)**	***V*, Å^3 (Z = 32)**	***V*, Å^3/Sn**	***a*, Å**	***c*, Å**	**V_{tet}, Å^3 (Z = 2)**	**V_{cub},Å^3/Sn**	***a*, Å**	***V*, Å^3**
185 GPa	8.159<br>8.165	543.13<br>544.33	16.97<br>17.01	3.241	4.591	48.22	24.11	2.365	13.23
180-185 GPa	8.180	547.34	17.10	3.229	4.614	48.11	24.11	2.365	13.23
190-196 GPa	8.130<br>8.140<br>8.135	537.37<br>539.35<br>538.33	16.79<br>16.85<br>16.82	3.229<br>3.233	4.577<br>4.582	47.74<br>47.90	23.95	2.360	13.14
201-208 GPa	8.1060	532.62	16.64	3.215	4.557	47.11	23.58	2.345<br>2.352	12.90<br>13.01
208-210 GPa	8.090	529.47	16.54	3.195	4.561	46.57	23.47	2.342	12.87

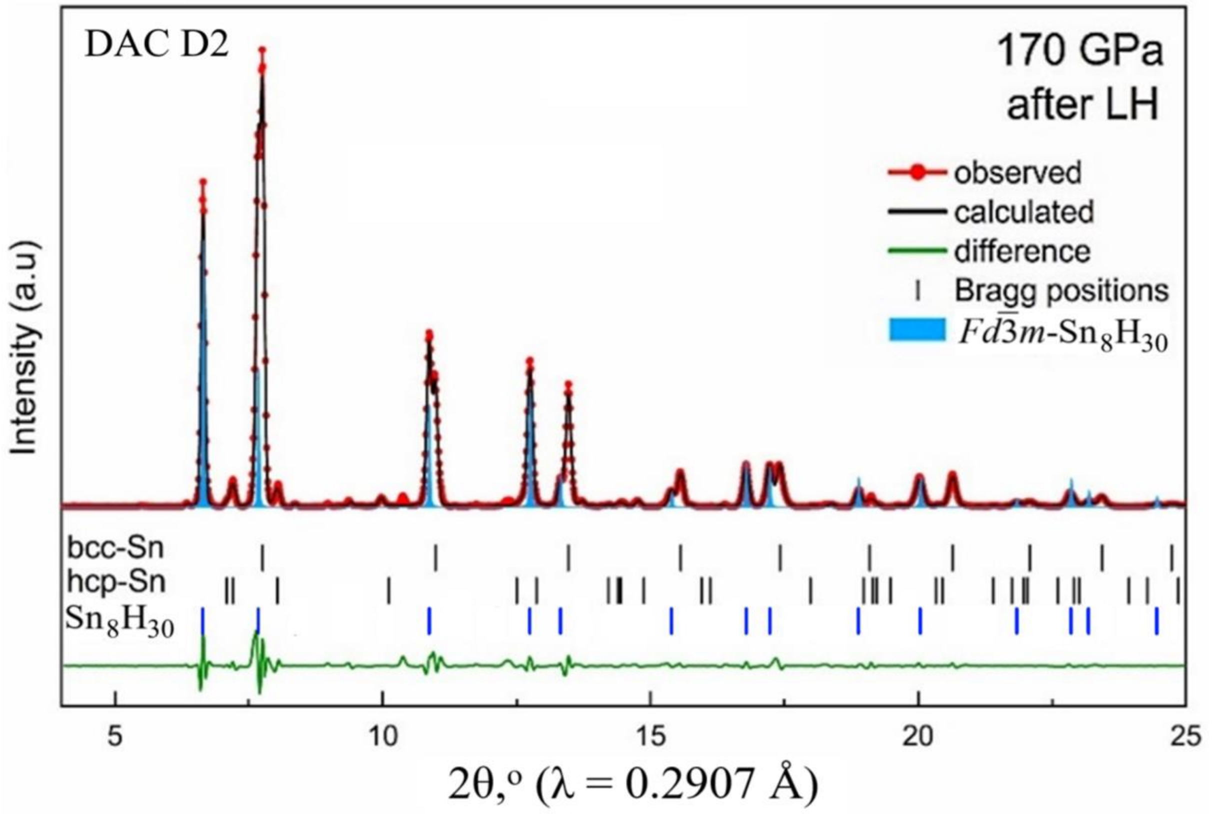

Figure S3. Experimental X-ray diffraction pattern of the results of laser heating of Sn/AB at a pressure of 170 GPa (DAC D2). XRD pattern was obtained at PETRA III in 2020. Cubic SnH_4 compound as the main product, along with unreacted Sn in hexagonal (*hcp*) and cubic (*bcc*) modifications are clearly seen. The Bragg peaks positions for the theoretical model, $Fd\bar{3}m$-Sn_8H_{30}, are also shown.

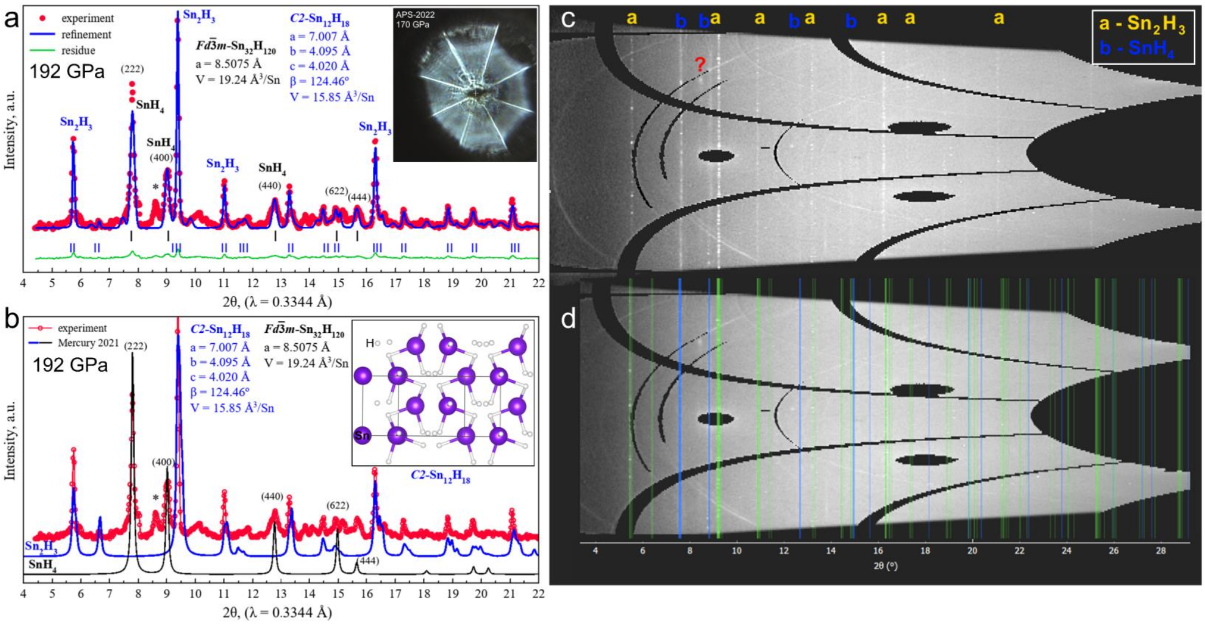

Figure S4. Main results of the XRD experiment performed at APS-2022. (a) The Le Bail refinement of the unit cell parameters of *C*2-$Sn_{12}H_{18}$ (= Sn_2H_3) and cubic SnH_4 (theoretical model is $Fd\bar{3}m$-$Sn_{32}H_{120}$ = Sn_8H_{30}) phases at a pressure of 192 GPa. Unidentified reflections are marked by asterisks. The experimental data, fitted line, and residues are shown in red, blue, and green, respectively. Inset: sample photograph at 171 GPa. (b) Comparison of experimental (red line) and calculated (blue and black lines) XRD patterns for $Sn_{12}H_{18}$ (= Sn_2H_3) and SnH_4 (theoretical model is $Fd\bar{3}m$-$Sn_{32}H_{120}$), respectively. Inset:

crystal structure of *C*2-$Sn_{12}H_{18}$. (c, d) Diffraction images ("cake") where one can see two groups of diffraction rings: relatively diffuse rings (b) corresponding to the cubic SnH_4, which can be obtained in pure form, and the second fraction, "dotted" rings (a) corresponding to $Sn_{12}H_{18}$ (= Sn_2H_3).

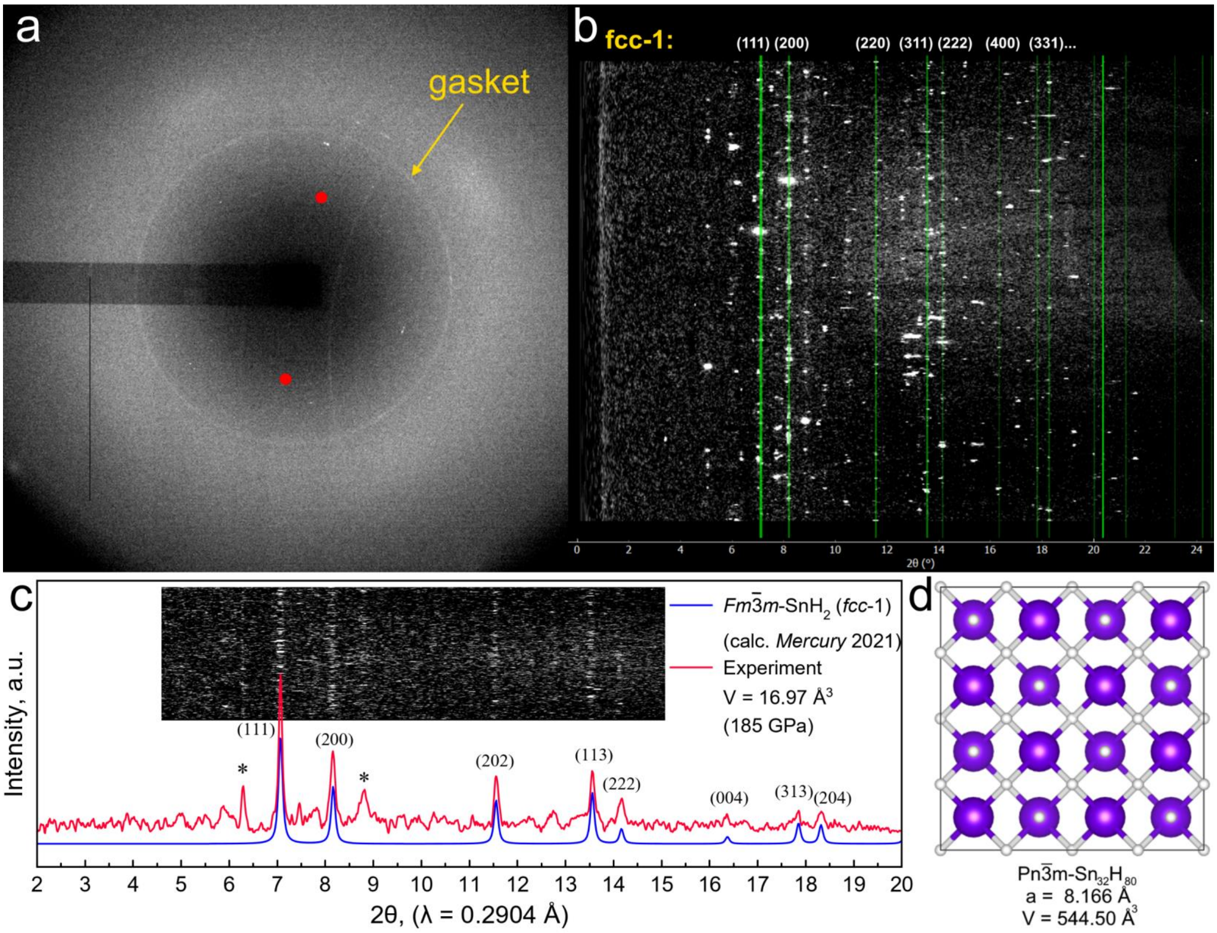

Figure S5. XRD patterns of hydrides synthesized in DAC M2 at 185 GPa. (a) A characteristic continuous diffraction ring from the gasket material. (b) A characteristic "dotted" pattern (coarse-crystalline) of tin hydrides. Green lines correspond to the *fcc*-1 phase (SnH_2). (c) X-ray diffraction pattern of the sample in DAC M2 at 185 GPa collected at a wavelength of λ = 0.2904 Å. Comparison of experimental and calculated XRD reflection intensities for the *fcc*-1 phase (refined using prototype $Fm\bar{3}m$-YH_2) is shown. Inset: diffraction image ("cake"). Unidentified reflections are marked by asterisks. (d) Crystal structure of one alternative candidate for the *fcc*-1 phase, $Sn_{32}H_{80}$ = $SnH_{2.5}$ which has a unit cell volume close to the experimental one.

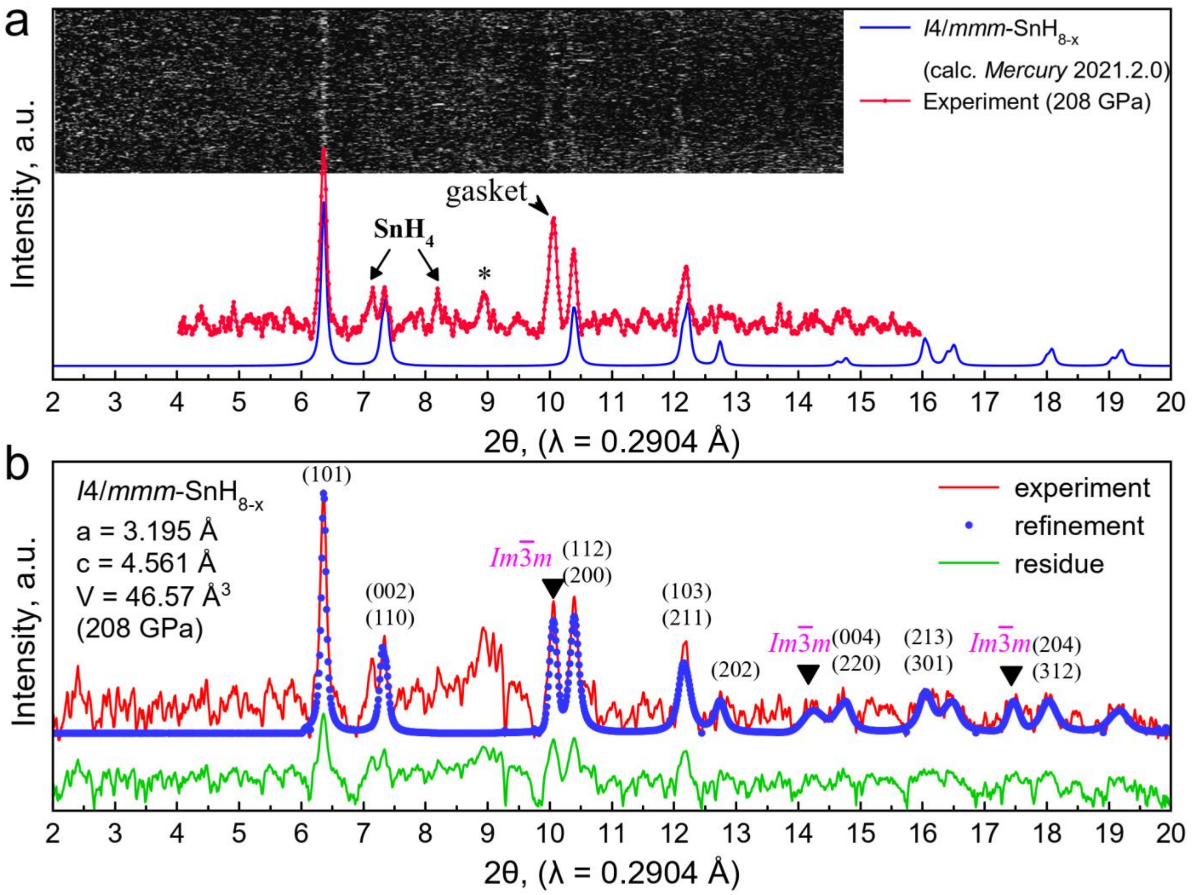

Figure S6. X-ray diffraction pattern of the edge of sample in DAC M2 at 208 GPa collected at a wavelength of λ = 0.2904 Å. (a) Comparison of experimental and calculated XRD intensities for the *fcc*-2 phase (refined as pseudocubic *I4/mmm*-SnH_{8-x}, Z = 2). Inset: the diffraction image ("cake"). (b) The Le Bail refinement of the unit cell parameters of the *I4/mmm*-SnH_{8-x} (*fcc*-2) phase. Unidentified reflections are marked by asterisks. The experimental data, fitted line, and residues are shown in red, blue, and green, respectively. "*Im*3̄*m*" denotes the unidentified reflection from the gasket or material of the DAC.

Table S6. Experimental and predicted structures (cif files) of various tin hydrides.

***fcc* SnH_4 at 171 GPa (experiment)**	***C*2/*m*-SnH_{14} at 180 GPa (calculations)**
data_cf_snh4<br>_audit_creation_date 2023-01-26<br>_audit_creation_method<br>;<br>Olex2 1.5<br>(compiled 2022.04.07 svn.rca3783a0 for OlexSys, GUI svn.r6498)<br>;<br>_shelx_SHELXL_version_number '2014/7'<br>_audit_contact_author_address ?<br>_audit_contact_author_email ?<br>_audit_contact_author_name ''<br>_audit_contact_author_phone ?<br>_publ_contact_author_id_orcid ?<br>_publ_section_references<br>;<br>Dolomanov, O.V., Bourhis, L.J., Gildea, R.J, Howard, J.A.K. & Puschmann, H.<br>(2009), J. Appl. Cryst. 42, 339-341.	data_SnH14_180GPa<br>_symmetry_cell_setting monoclinic<br>_symmetry_space_group_name_H-M 'C 2/m'<br>_symmetry_Int_Tables_number 12<br>_space_group_name_Hall '-C 2y'<br>loop_<br>_symmetry_equiv_pos_site_id<br>_symmetry_equiv_pos_as_xyz<br>1 x,y,z<br>2 -x,y,-z<br>3 -x,-y,-z<br>4 x,-y,z<br>5 1/2+x,1/2+y,z<br>6 1/2-x,1/2+y,-z<br>7 1/2-x,1/2-y,-z<br>8 1/2+x,1/2-y,z<br>_cell_length_a 7.756

```
Sheldrick, G.M. (2015). Acta Cryst. C71, 3-8.
;
_chemical_name_common             ?
_chemical_name_systematic         'tin hydride'
_chemical_formula_moiety          '0.021(Sn48)'
_chemical_formula_sum             'Sn'
_chemical_formula_weight          118.69
_chemical_melting_point           ?
loop_
  _atom_type_symbol
  _atom_type_description
  _atom_type_scat_dispersion_real
  _atom_type_scat_dispersion_imag
  _atom_type_scat_source
 'Sn' 'Sn' -0.6925 2.4050
 'International Tables Vol C Tables 4.2.6.8 and 6.1.1.4'

_shelx_space_group_comment
;
The symmetry employed for this shelxl refinement is uniquely
defined by the following loop, which should always be used
as a source of symmetry information in preference to the
above space-group names. They are only intended as
comments.
;
_space_group_crystal_system       'cubic'
_space_group_IT_number            225
_space_group_name_H-M_alt         'F m -3 m'
_space_group_name_Hall            '-F 4 2 3'
loop_
  _space_group_symop_operation_xyz
 'x, y, z'
 '-x, -y, z'
 '-x, y, -z'
 'x, -y, -z'
 'z, x, y'
 'z, -x, -y'
 '-z, -x, y'
 '-z, x, -y'
 'y, z, x'
 '-y, z, -x'
 'y, -z, -x'
 '-y, -z, x'
 'y, x, -z'
 '-y, -x, -z'
 'y, -x, z'
 '-y, x, z'
 'x, z, -y'
 '-x, z, y'
 '-x, -z, -y'
 'x, -z, y'
 'z, y, -x'
 'z, -y, x'
 '-z, y, x'
 '-z, -y, -x'
 'x, y+1/2, z+1/2'
 '-x, -y+1/2, z+1/2'
 '-x, y+1/2, -z+1/2'
 'x, -y+1/2, -z+1/2'
 'z, x+1/2, y+1/2'
 'z, -x+1/2, -y+1/2'
 '-z, -x+1/2, y+1/2'
 '-z, x+1/2, -y+1/2'
 'y, z+1/2, x+1/2'
 '-y, z+1/2, -x+1/2'
 'y, -z+1/2, -x+1/2'
```

```
_cell_length_b          2.871
_cell_length_c          3.797
_cell_angle_alpha       90
_cell_angle_beta        118.34
_cell_angle_gamma       90
_cell_volume            74.42
loop_
_atom_site_label
_atom_site_type_symbol
_atom_site_fract_x
_atom_site_fract_y
_atom_site_fract_z
_atom_site_U_iso_or_equiv
_atom_site_thermal_displace_type
Sn1 Sn 0 0.5 0 0.0000 Uiso
H1 H 0.26312 0.22116 0.40715 0.0000 Uiso
H3 H -0.18543 0 -0.01272 0.0000 Uiso
H4 H -0.06856 0 -0.3698 0.0000 Uiso
H6 H 0 0.5 0.5 0.0000 Uiso
H2 H 0.13137 0.5 -0.2995 0.0000 Uiso
H5 H -0.30277 0.5 -0.1822 0.0000 Uiso
H1 H -0.26312 0.22116 -0.40715 0.0000 Uiso
H3 H 0.18543 0 0.01272 0.0000 Uiso
H4 H 0.06856 0 0.3698 0.0000 Uiso
H2 H -0.13137 0.5 0.2995 0.0000 Uiso
H5 H 0.30277 0.5 0.1822 0.0000 Uiso
H1 H -0.26312 0.77884 -0.40715 0.0000 Uiso
H1 H 0.26312 0.77884 0.40715 0.0000 Uiso
Sn1 Sn 0 -0.5 0 0.0000 Uiso
Sn1 Sn 0 0.5 1 0.0000 Uiso
Sn1 Sn 0 1.5 0 0.0000 Uiso
H3 H -0.18543 1 -0.01272 0.0000 Uiso
H3 H 0.18543 1 0.01272 0.0000 Uiso
H4 H -0.06856 1 -0.3698 0.0000 Uiso
H4 H 0.06856 1 0.3698 0.0000 Uiso
H6 H 0 0.5 -0.5 0.0000 Uiso
H7 H 0 0 0.5 0.0000 Uiso

#END
```

Symmetrized Sn sublattice (*R*-3*m*) in *C*2-$Sn_{12}H_{18}$ at 200 GPa (calculations)

```
# CIF file
# This file was generated by FINDSYM
# Harold T. Stokes, Branton J. Campbell, Dorian M. Hatch
# Brigham Young University, Provo, Utah, USA

data_findsym-output

_symmetry_space_group_name_H-M 'R -3 2/m (hexagonal axes)'
_symmetry_Int_Tables_number 166

_cell_length_a      4.07646
_cell_length_b      4.07646
_cell_length_c      9.94541
```

```
'-y, -z+1/2, x+1/2'
'y, x+1/2, -z+1/2'
'-y, -x+1/2, -z+1/2'
'y, -x+1/2, z+1/2'
'-y, x+1/2, z+1/2'
'x, z+1/2, -y+1/2'
'-x, z+1/2, y+1/2'
'-x, -z+1/2, -y+1/2'
'x, -z+1/2, y+1/2'
'z, y+1/2, -x+1/2'
'z, -y+1/2, x+1/2'
'-z, y+1/2, x+1/2'
'-z, -y+1/2, -x+1/2'
'x+1/2, y, z+1/2'
'-x+1/2, -y, z+1/2'
'-x+1/2, y, -z+1/2'
'x+1/2, -y, -z+1/2'
'z+1/2, x, y+1/2'
'z+1/2, -x, -y+1/2'
'-z+1/2, -x, y+1/2'
'-z+1/2, x, -y+1/2'
'y+1/2, z, x+1/2'
'-y+1/2, z, -x+1/2'
'y+1/2, -z, -x+1/2'
'-y+1/2, -z, x+1/2'
'y+1/2, x, -z+1/2'
'-y+1/2, -x, -z+1/2'
'y+1/2, -x, z+1/2'
'-y+1/2, x, z+1/2'
'x+1/2, z, -y+1/2'
'-x+1/2, z, y+1/2'
'-x+1/2, -z, -y+1/2'
'x+1/2, -z, y+1/2'
'z+1/2, y, -x+1/2'
'z+1/2, -y, x+1/2'
'-z+1/2, y, x+1/2'
'-z+1/2, -y, -x+1/2'
'x+1/2, y+1/2, z'
'-x+1/2, -y+1/2, z'
'-x+1/2, y+1/2, -z'
'x+1/2, -y+1/2, -z'
'z+1/2, x+1/2, y'
'z+1/2, -x+1/2, -y'
'-z+1/2, -x+1/2, y'
'-z+1/2, x+1/2, -y'
'y+1/2, z+1/2, x'
'-y+1/2, z+1/2, -x'
'y+1/2, -z+1/2, -x'
'-y+1/2, -z+1/2, x'
'y+1/2, x+1/2, -z'
'-y+1/2, -x+1/2, -z'
'y+1/2, -x+1/2, z'
'-y+1/2, x+1/2, z'
'x+1/2, z+1/2, -y'
'-x+1/2, z+1/2, y'
'-x+1/2, -z+1/2, -y'
'x+1/2, -z+1/2, y'
'z+1/2, y+1/2, -x'
'z+1/2, -y+1/2, x'
'-z+1/2, y+1/2, x'
'-z+1/2, -y+1/2, -x'
'-x, -y, -z'
'x, y, -z'
'x, -y, z'
'-x, y, z'
'-z, -x, -y'
'-z, x, y'
```

```
_cell_angle_alpha   90.00000
_cell_angle_beta    90.00000
_cell_angle_gamma  120.00000
_cell_volume 143.1264

loop_
_space_group_symop_operation_xyz
x,y,z
-y,x-y,z
-x+y,-x,z
y,x,-z
-x,-x+y,-z
x-y,-y,-z
-x,-y,-z
y,-x+y,-z
x-y,x,-z
-y,-x,z
x,x-y,z
-x+y,y,z
x+1/3,y+2/3,z+2/3
-y+1/3,x-y+2/3,z+2/3
-x+y+1/3,-x+2/3,z+2/3
y+1/3,x+2/3,-z+2/3
-x+1/3,-x+y+2/3,-z+2/3
x-y+1/3,-y+2/3,-z+2/3
-x+1/3,-y+2/3,-z+2/3
y+1/3,-x+y+2/3,-z+2/3
x-y+1/3,x+2/3,-z+2/3
-y+1/3,-x+2/3,z+2/3
x+1/3,x-y+2/3,z+2/3
-x+y+1/3,y+2/3,z+2/3
x+2/3,y+1/3,z+1/3
-y+2/3,x-y+1/3,z+1/3
-x+y+2/3,-x+1/3,z+1/3
y+2/3,x+1/3,-z+1/3
-x+2/3,-x+y+1/3,-z+1/3
x-y+2/3,-y+1/3,-z+1/3
-x+2/3,-y+1/3,-z+1/3
y+2/3,-x+y+1/3,-z+1/3
x-y+2/3,x+1/3,-z+1/3
-y+2/3,-x+1/3,z+1/3
x+2/3,x-y+1/3,z+1/3
-x+y+2/3,y+1/3,z+1/3

loop_
_atom_site_label
_atom_site_type_symbol
_atom_site_fract_x
_atom_site_fract_y
_atom_site_fract_z
_atom_site_occupancy
Sn1 Sn   0.00000   0.00000  -0.24147   1.00000
Sn2 Sn   0.00000   0.00000   0.00000   1.00000
```

'z, x, -y'<br>'z, -x, y'<br>'-y, -z, -x'<br>'y, -z, x'<br>'-y, z, x'<br>'y, z, -x'<br>'-y, -x, z'<br>'y, x, z'<br>'-y, x, -z'<br>'y, -x, -z'<br>'-x, -z, y'<br>'x, -z, -y'<br>'x, z, y'<br>'-x, z, -y'<br>'-z, -y, x'<br>'-z, y, -x'<br>'z, -y, -x'<br>'z, y, x'<br>'-x, -y+1/2, -z+1/2'<br>'x, y+1/2, -z+1/2'<br>'x, -y+1/2, z+1/2'<br>'-x, y+1/2, z+1/2'<br>'-z, -x+1/2, -y+1/2'<br>'-z, x+1/2, y+1/2'<br>'z, x+1/2, -y+1/2'<br>'z, -x+1/2, y+1/2'<br>'-y, -z+1/2, -x+1/2'<br>'y, -z+1/2, x+1/2'<br>'-y, z+1/2, x+1/2'<br>'y, z+1/2, -x+1/2'<br>'-y, -x+1/2, z+1/2'<br>'y, x+1/2, z+1/2'<br>'-y, x+1/2, -z+1/2'<br>'y, -x+1/2, -z+1/2'<br>'-x, -z+1/2, y+1/2'<br>'x, -z+1/2, -y+1/2'<br>'x, z+1/2, y+1/2'<br>'-x, z+1/2, -y+1/2'<br>'-z, -y+1/2, x+1/2'<br>'-z, y+1/2, -x+1/2'<br>'z, -y+1/2, -x+1/2'<br>'z, y+1/2, x+1/2'<br>'-x+1/2, -y, -z+1/2'<br>'x+1/2, y, -z+1/2'<br>'x+1/2, -y, z+1/2'<br>'-x+1/2, y, z+1/2'<br>'-z+1/2, -x, -y+1/2'<br>'-z+1/2, x, y+1/2'<br>'z+1/2, x, -y+1/2'<br>'z+1/2, -x, y+1/2'<br>'-y+1/2, -z, -x+1/2'<br>'y+1/2, -z, x+1/2'<br>'-y+1/2, z, x+1/2'<br>'y+1/2, z, -x+1/2'<br>'-y+1/2, -x, z+1/2'<br>'y+1/2, x, z+1/2'<br>'-y+1/2, x, -z+1/2'<br>'y+1/2, -x, -z+1/2'<br>'-x+1/2, -z, y+1/2'<br>'x+1/2, -z, -y+1/2'<br>'x+1/2, z, y+1/2'<br>'-x+1/2, z, -y+1/2'<br>'-z+1/2, -y, x+1/2'<br>'-z+1/2, y, -x+1/2'<br>'z+1/2, -y, -x+1/2'<br>'z+1/2, y, x+1/2'<br>'-x+1/2, -y+1/2, -z'<br>'x+1/2, y+1/2, -z'	

```
 'x+1/2, -y+1/2, z'
 '-x+1/2, y+1/2, z'
 '-z+1/2, -x+1/2, -y'
 '-z+1/2, x+1/2, y'
 'z+1/2, x+1/2, -y'
 'z+1/2, -x+1/2, y'
 '-y+1/2, -z+1/2, -x'
 'y+1/2, -z+1/2, x'
 '-y+1/2, z+1/2, x'
 'y+1/2, z+1/2, -x'
 '-y+1/2, -x+1/2, z'
 'y+1/2, x+1/2, z'
 '-y+1/2, x+1/2, -z'
 'y+1/2, -x+1/2, -z'
 '-x+1/2, -z+1/2, y'
 'x+1/2, -z+1/2, -y'
 'x+1/2, z+1/2, y'
 '-x+1/2, z+1/2, -y'
 '-z+1/2, -y+1/2, x'
 '-z+1/2, y+1/2, -x'
 'z+1/2, -y+1/2, -x'
 'z+1/2, y+1/2, x'

_cell_length_a                    4.244(3)
_cell_length_b                    4.244(3)
_cell_length_c                    4.244(3)
_cell_angle_alpha                 90
_cell_angle_beta                  90
_cell_angle_gamma                 90
_cell_volume                      76.44(14)
_cell_formula_units_Z             4
_cell_measurement_reflns_used     25
_cell_measurement_temperature     293(2)
_cell_measurement_theta_max       14.4550
_cell_measurement_theta_min       4.5250
_shelx_estimated_absorpt_T_max    0.988
_shelx_estimated_absorpt_T_min    0.988
_exptl_absorpt_coefficient_mu     24.888
_exptl_absorpt_correction_T_max   ?
_exptl_absorpt_correction_T_min   ?
_exptl_absorpt_correction_type    none
_exptl_absorpt_process_details    ?
_exptl_absorpt_special_details    ?
_exptl_crystal_colour             metallic
_exptl_crystal_colour_lustre      metallic
_exptl_crystal_density_diffrn     10.313
_exptl_crystal_density_meas       ?
_exptl_crystal_density_method     ?
_exptl_crystal_description        irregular
_exptl_crystal_F_000              200
_exptl_crystal_preparation        ?
_exptl_crystal_size_max           0.0005
_exptl_crystal_size_mid           0.0005
_exptl_crystal_size_min           0.0005
_exptl_transmission_factor_max    ?
_exptl_transmission_factor_min    ?
_diffrn_reflns_av_R_equivalents   0.1356
_diffrn_reflns_av_unetI/netI      0.0827
_diffrn_reflns_Laue_measured_fraction_full  0.778
_diffrn_reflns_Laue_measured_fraction_max  0.625
_diffrn_reflns_limit_h_max        5
_diffrn_reflns_limit_h_min        -6
_diffrn_reflns_limit_k_max        5
_diffrn_reflns_limit_k_min        -5
_diffrn_reflns_limit_l_max        2
_diffrn_reflns_limit_l_min        -1
_diffrn_reflns_number             29
_diffrn_reflns_point_group_measured_fraction_full  0.778
```

```
_diffrn_reflns_point_group_measured_fraction_max  0.625
_diffrn_reflns_theta_full          11.574
_diffrn_reflns_theta_max           14.428
_diffrn_reflns_theta_min           4.519
_diffrn_ambient_temperature        293(2)
_diffrn_detector                  'Pixel detector'
_diffrn_detector_area_resol_mean   5.8140
_diffrn_detector_type             'Pilatus 1M CdTe'
_diffrn_measured_fraction_theta_full  0.778
_diffrn_measured_fraction_theta_max  0.625
_diffrn_measurement_details
;
List of Runs (angles in degrees, time in seconds):

  # Type    Start    End  Width  t~exp~   \w     \q     \k     \f
Frames
---------------------------------------------------------------------------
  1  \w    -32.00  32.00   0.50    1.00    --    0.00   0.00   0.00
128
;
_diffrn_measurement_device         '13IDD @ APS'
_diffrn_measurement_device_type    'LH Table'
_diffrn_measurement_method        'omega rotation'
_diffrn_orient_matrix_type
 'CrysAlisPro convention (1999,Acta A55,543-557)'
_diffrn_orient_matrix_UB_11       -0.0136159167
_diffrn_orient_matrix_UB_12       -0.0060303500
_diffrn_orient_matrix_UB_13       0.0776876667
_diffrn_orient_matrix_UB_21       -0.0288921993
_diffrn_orient_matrix_UB_22       0.0731562500
_diffrn_orient_matrix_UB_23       0.0012496493
_diffrn_orient_matrix_UB_31       -0.0721157857
_diffrn_orient_matrix_UB_32       -0.0276921000
_diffrn_orient_matrix_UB_33       -0.0150944143
_diffrn_radiation_monochromator    synchrotron
_diffrn_radiation_probe            x-ray
_diffrn_radiation_type            synchrotron
_diffrn_radiation_wavelength       0.3344
_diffrn_source                    synchrotron
_reflns_Friedel_coverage           0.000
_reflns_Friedel_fraction_full      .
_reflns_Friedel_fraction_max       .
_reflns_number_gt                 10
_reflns_number_total              10
_reflns_special_details
;
 Reflections were merged by SHELXL according to the
crystal
 class for the calculation of statistics and refinement.

  _reflns_Friedel_fraction is defined as the number of unique
 Friedel pairs measured divided by the number that would be
 possible theoretically, ignoring centric projections and
 systematic absences.
;
_reflns_threshold_expression       'I > 2\s(I)'
_computing_cell_refinement         'CrysAlisPro 1.171.40.69a
(Rigaku OD, 2020)'
_computing_data_collection         'CrysAlisPro 1.171.40.69a
(Rigaku OD, 2020)'
_computing_data_reduction          'CrysAlisPro 1.171.40.69a
(Rigaku OD, 2020)'
_computing_molecular_graphics      'Olex2 1.5 (Dolomanov
et al., 2009)'
_computing_publication_material    'Olex2 1.5 (Dolomanov et
al., 2009)'
_computing_structure_refinement    'SHELXL 2014/7
(Sheldrick, 2015)'
```

_computing_structure_solution ?<br>_refine_diff_density_max 1.134<br>_refine_diff_density_min -1.995<br>_refine_diff_density_rms 0.464<br>_refine_ls_extinction_coef 0.5(6)<br>_refine_ls_extinction_expression<br>'Fc^*^=kFc[1+0.001xFc^2^\l^3^/sin(2\q)]^-1/4^'<br>_refine_ls_extinction_method 'SHELXL-2014/7 (Sheldrick 2014'<br>_refine_ls_goodness_of_fit_ref 1.240<br>_refine_ls_hydrogen_treatment undef<br>_refine_ls_matrix_type full<br>_refine_ls_number_parameters 3<br>_refine_ls_number_reflns 10<br>_refine_ls_number_restraints 0<br>_refine_ls_R_factor_all 0.0475<br>_refine_ls_R_factor_gt 0.0475<br>_refine_ls_restrained_S_all 1.240<br>_refine_ls_shift/su_max 0.000<br>_refine_ls_shift/su_mean 0.000<br>_refine_ls_structure_factor_coef Fsqd<br>_refine_ls_weighting_details<br>'w=1/[\s^2^(Fo^2^)+22.1894P] where P=(Fo^2^+2Fc^2^)/3'<br>_refine_ls_weighting_scheme calc<br>_refine_ls_wR_factor_gt 0.0960<br>_refine_ls_wR_factor_ref 0.0960<br>_refine_special_details ?<br>_olex2_refinement_description<br>;<br>;<br>_atom_sites_solution_hydrogens .<br>_atom_sites_solution_primary ?<br>_atom_sites_solution_secondary ?<br>loop_<br>_atom_site_label<br>_atom_site_type_symbol<br>_atom_site_fract_x<br>_atom_site_fract_y<br>_atom_site_fract_z<br>_atom_site_U_iso_or_equiv<br>_atom_site_adp_type<br>_atom_site_occupancy<br>_atom_site_site_symmetry_order<br>_atom_site_calc_flag<br>_atom_site_refinement_flags_posn<br>_atom_site_refinement_flags_adp<br>_atom_site_refinement_flags_occupancy<br>_atom_site_disorder_assembly<br>_atom_site_disorder_group<br>Sn01 Sn 0.0000 0.0000 0.0000 0.024(6) Uani 1 48 d S T P . .<br><br>loop_<br>_atom_site_aniso_label<br>_atom_site_aniso_U_11<br>_atom_site_aniso_U_22<br>_atom_site_aniso_U_33<br>_atom_site_aniso_U_23<br>_atom_site_aniso_U_13<br>_atom_site_aniso_U_12<br>Sn01 0.024(6) 0.024(6) 0.024(6) 0.000 0.000 0.000<br><br>_geom_special_details<br>;<br>All esds (except the esd in the dihedral angle between two l.s. planes)<br>are estimated using the full covariance matrix. The cell esds are taken	

into account individually in the estimation of esds in distances, angles<br>and torsion angles; correlations between esds in cell parameters are only<br>used when they are defined by crystal symmetry. An approximate (isotropic)<br>treatment of cell esds is used for estimating esds involving l.s. planes.<br>;<br>loop_<br>_geom_bond_atom_site_label_1<br>_geom_bond_atom_site_label_2<br>_geom_bond_distance<br>_geom_bond_site_symmetry_2<br>_geom_bond_publ_flag<br>Sn01 Sn01 3.0010(18) 73 ?<br>Sn01 Sn01 3.0010(18) 49_554 ?<br>Sn01 Sn01 3.0010(18) 25_554 ?<br>Sn01 Sn01 3.0010(18) 25_544 ?<br>Sn01 Sn01 3.0010(18) 73_545 ?<br>Sn01 Sn01 3.0010(18) 73_445 ?<br>Sn01 Sn01 3.0010(18) 49_455 ?<br>Sn01 Sn01 3.0010(18) 49 ?<br>Sn01 Sn01 3.0010(18) 25_545 ?<br>Sn01 Sn01 3.0010(18) 25 ?<br>Sn01 Sn01 3.0010(18) 73_455 ?<br>Sn01 Sn01 3.0010(18) 49_454 ?<br><br>loop_<br>_geom_angle_atom_site_label_1<br>_geom_angle_atom_site_label_2<br>_geom_angle_atom_site_label_3<br>_geom_angle<br>_geom_angle_site_symmetry_1<br>_geom_angle_site_symmetry_3<br>_geom_angle_publ_flag<br>Sn01 Sn01 Sn01 120.0 73 25_544 ?<br>Sn01 Sn01 Sn01 120.0 25_545 73_455 ?<br>Sn01 Sn01 Sn01 60.0 49 73_545 ?<br>Sn01 Sn01 Sn01 180.0 73 73_445 ?<br>Sn01 Sn01 Sn01 90.0 25 25_545 ?<br>Sn01 Sn01 Sn01 60.0 25_544 73_445 ?<br>Sn01 Sn01 Sn01 120.0 25_544 73_455 ?<br>Sn01 Sn01 Sn01 60.0 73 49 ?<br>Sn01 Sn01 Sn01 120.0 73 25_545 ?<br>Sn01 Sn01 Sn01 120.0 25_544 49 ?<br>Sn01 Sn01 Sn01 120.0 49_554 25_545 ?<br>Sn01 Sn01 Sn01 120.0 73_445 49 ?<br>Sn01 Sn01 Sn01 120.0 49_455 73_545 ?<br>Sn01 Sn01 Sn01 60.0 73 25 ?<br>Sn01 Sn01 Sn01 60.0 49_454 73_455 ?<br>Sn01 Sn01 Sn01 180.0 25_544 25 ?<br>Sn01 Sn01 Sn01 60.0 25_554 49_554 ?<br>Sn01 Sn01 Sn01 120.0 73_445 25 ?<br>Sn01 Sn01 Sn01 60.0 73_445 25_545 ?<br>Sn01 Sn01 Sn01 60.0 49 25 ?<br>Sn01 Sn01 Sn01 180.0 25_554 25_545 ?<br>Sn01 Sn01 Sn01 120.0 73 49_454 ?<br>Sn01 Sn01 Sn01 60.0 25_544 73_545 ?<br>Sn01 Sn01 Sn01 60.0 25_544 49_454 ?<br>Sn01 Sn01 Sn01 120.0 49_454 73_545 ?<br>Sn01 Sn01 Sn01 60.0 73_445 49_454 ?<br>Sn01 Sn01 Sn01 60.0 25_545 73_545 ?<br>Sn01 Sn01 Sn01 180.0 49 49_454 ?<br>Sn01 Sn01 Sn01 120.0 49 73_455 ?<br>Sn01 Sn01 Sn01 120.0 25 49_454 ?<br>Sn01 Sn01 Sn01 60.0 49_455 73_455 ?<br>Sn01 Sn01 Sn01 60.0 73 25_554 ?	

Sn01 Sn01 Sn01 90.0 49_454 49_554 ?<br>Sn01 Sn01 Sn01 90.0 25_544 25_554 ?<br>Sn01 Sn01 Sn01 180.0 49_455 49_554 ?<br>Sn01 Sn01 Sn01 120.0 73_445 25_554 ?<br>Sn01 Sn01 Sn01 90.0 25_544 25_545 ?<br>Sn01 Sn01 Sn01 120.0 49 25_554 ?<br>Sn01 Sn01 Sn01 60.0 49 25_545 ?<br>Sn01 Sn01 Sn01 90.0 25 25_554 ?<br>Sn01 Sn01 Sn01 120.0 49_454 25_545 ?<br>Sn01 Sn01 Sn01 60.0 49_454 25_554 ?<br>Sn01 Sn01 Sn01 60.0 49_455 25_545 ?<br>Sn01 Sn01 Sn01 120.0 73 49_455 ?<br>Sn01 Sn01 Sn01 90.0 73 73_545 ?<br>Sn01 Sn01 Sn01 120.0 25_544 49_455 ?<br>Sn01 Sn01 Sn01 90.0 73_445 73_545 ?<br>Sn01 Sn01 Sn01 60.0 73_445 49_455 ?<br>Sn01 Sn01 Sn01 120.0 25 73_545 ?<br>Sn01 Sn01 Sn01 90.0 49 49_455 ?<br>Sn01 Sn01 Sn01 120.0 25_554 73_545 ?<br>Sn01 Sn01 Sn01 60.0 25 49_455 ?<br>Sn01 Sn01 Sn01 60.0 49_554 73_545 ?<br>Sn01 Sn01 Sn01 90.0 49_454 49_455 ?<br>Sn01 Sn01 Sn01 90.0 73 73_455 ?<br>Sn01 Sn01 Sn01 120.0 25_554 49_455 ?<br>Sn01 Sn01 Sn01 90.0 73_445 73_455 ?<br>Sn01 Sn01 Sn01 60.0 73 49_554 ?<br>Sn01 Sn01 Sn01 60.0 25 73_455 ?<br>Sn01 Sn01 Sn01 60.0 25_544 49_554 ?<br>Sn01 Sn01 Sn01 60.0 25_554 73_455 ?<br>Sn01 Sn01 Sn01 120.0 73_445 49_554 ?<br>Sn01 Sn01 Sn01 120.0 49_554 73_455 ?<br>Sn01 Sn01 Sn01 90.0 49 49_554 ?<br>Sn01 Sn01 Sn01 180.0 73_545 73_455 ?<br>Sn01 Sn01 Sn01 120.0 25 49_554 ?<br><br>_shelx_res_file<br>;<br>cf_snh4.res created by SHELXL-2014/7<br><br>TITL snh_p02_q3_pos130<br>CELL 0.3344 4.244 4.244 4.244 90 90 90<br>ZERR 4 0.002599 0.002599 0.002599 0 0 0<br>LATT 4<br>SYMM -X,-Y,+Z<br>SYMM -X,+Y,-Z<br>SYMM +X,-Y,-Z<br>SYMM +Z,+X,+Y<br>SYMM +Z,-X,-Y<br>SYMM -Z,-X,+Y<br>SYMM -Z,+X,-Y<br>SYMM +Y,+Z,+X<br>SYMM -Y,+Z,-X<br>SYMM +Y,-Z,-X<br>SYMM -Y,-Z,+X<br>SYMM +Y,+X,-Z<br>SYMM -Y,-X,-Z<br>SYMM +Y,-X,+Z<br>SYMM -Y,+X,+Z<br>SYMM +X,+Z,-Y<br>SYMM -X,+Z,+Y<br>SYMM -X,-Z,-Y<br>SYMM +X,-Z,+Y<br>SYMM +Z,+Y,-X<br>SYMM +Z,-Y,+X<br>SYMM -Z,+Y,+X<br>SYMM -Z,-Y,-X<br>SFAC Sn<br>DISP Sn -0.6925 2.405 4756.1985	

```
UNIT 4

L.S. 10 0 0
PLAN  5
SIZE 0.0005 0.0005 0.0005
CONF
MORE -1
fmap 2 53
ACTA
REM <olex2.extras>
REM <HklSrc "%.\\cF_SnH4.hkl">
REM </olex2.extras>

WGHT    0.000000   22.189402
EXTI    0.541500
FVAR       1.01538
SN01  1    0.000000    0.000000    0.000000    10.02083
0.02443    0.02443 =
        0.02443    0.00000    0.00000    0.00000
HKLF 4

REM  snh_p02_q3_pos130
REM R1 =  0.0475 for      10 Fo > 4sig(Fo)  and  0.0475 for
all      10 data
REM      3 parameters refined using      0 restraints

END

WGHT      0.0000     19.9852

REM Highest difference peak  1.134,  deepest hole -1.995,  1-
sigma level  0.464
Q1    1   0.2500  0.2500 -0.1231  10.25000  0.05    1.13
Q2    1   0.0000  0.3032  0.0000  10.12500  0.05    0.94
Q3    1   0.0000  0.0000  0.0773  10.12500  0.05    0.91
Q4    1   0.1739  0.0000  0.0000  10.12500  0.05    0.90
Q5    1   0.0000 -0.3415  0.1585  10.25000  0.05    0.72
;
_shelx_res_checksum               69794
_shelx_hkl_file
;
  -2   0   010079.50   48.90   1
   0  -2   012715.90   83.54   1
  -2  -2   0 7816.28   71.28   1
   1  -3   1 6288.93  151.82   1
  -1   3   1 5994.86  153.40   1
  -3   1  -1 7997.15  113.20   1
  -1   3  -1 7219.69  163.26   1
  -3   3   1 3471.32  189.81   1
   3  -3   1 3498.64  184.45   1
   3   3   1 2777.11   98.18   1
  -3   3  -1 4243.44  191.87   1
   0  -4   0 3709.52  173.03   1
   0   4   0 3934.57  176.98   1
   4  -2   2 2634.75  170.81   1
   4   0   2 3645.09  107.95   1
  -2   4   0 3888.53  210.43   1
   2  -4   0 3751.22  208.44   1
   2   4   2 2135.76  161.07   1
   4   2   2  751.01   68.82   1
   5  -1   1  970.27  147.15   1
  -1  -5  -1 1070.62  209.66   1
   1   5   1 2075.23  203.40   1
  -1  -5   1 1685.69  208.09   1
  -5   1  -1 2010.98  150.14   1
   5  -3   1  940.45  232.90   1
  -3  -5  -1  438.57  178.09   1
   3   5   1 1245.79  187.15   1
```

-5 3 -1 1368.40 233.43 1<br>-6 2 0 672.27 208.70 1<br>0 0 0 0.00 0.00 0<br>;<br>_shelx_hkl_checksum 70650<br>_olex2_submission_special_instructions 'No special instructions were received'<br>_oxdiff_exptl_absorpt_empirical_details<br>;<br>Empirical correction (ABSPACK) includes:<br>;<br>_oxdiff_exptl_absorpt_empirical_full_max 1.000<br>_oxdiff_exptl_absorpt_empirical_full_min 1.000	

Table S7. Experimental unit cell parameters of proposed distorted $Im\overline{3}m$ (Z = 2) phase and $P6_3/mmc$-Sn (Z = 2) obtained from the exteriments at PETRA-III in 2020 at different pressures (DAC M2).

Experiment and pressure	*a*, Å	*b*, Å	*c*, Å	*V*, Å^3	*a*, Å	*c*, Å	*V*, Å^3
P02 (h1), 180 GPa	2.895	2.895	2.895	24.26	2.633	4.581	27.51
P02 (h3), 185 GPa	2.885	2.935	2.845	24.09	2.634	4.595	27.61
P03, 190-196 GPa	2.885<br>2.885	2.865<br>2.925	2.895<br>2.835	23.93<br>23.92	2.716	4.093	26.15
P04, 201-208 GPa	2.863<br>2.870	2.863<br>2.870	2.863<br>2.870	23.47<br>23.64	2.634	4.435	26.65
P05, 208-210 GPa	2.855	2.905	2.825	23.43	2.621	4.415	26.32

Table S8. Calculated unit cell parameters of $Im\overline{3}m$-Sn (Z=2) and $Fd\overline{3}m$-Sn_8H_{30} (Z = 8 or 32).

Pressure, GPa	a, Å (Sn)	V, Å^3 (Sn)	a, Å (Z=8)	V, Å^3 (Z= 8)	V, Å^3 (Z= 32)	V, Å^3/Sn
160	3.0503	28.38	5.4737	164.0	656.0	20.50
180	3.0195	27.53	5.4115	158.47	633.88	19.81
200	2.9922	26.79	5.3556	153.61	614.44	19.20
210	2.9799	26.46	5.3294	151.37	605.48	18.92
220	2.9674	26.13	5.3044	149.25	597.0	18.65
230	2.955	25.82	-	-	-	-
240	2.944	25.52	-	-	-	-

Table S9. Experimental unit cell parameters of $C2/m$-SnH_{14} (Z = 2) at different pressures, the experiment was done at PETRA III in 2020. “h1” denotes the first laser heating, “h3” – the third laser heating.

Experiment and pressure	a, Å	b, Å	c, Å	β,º	V, Å^3	V, Å^3/Sn
DAC D2, 193 GPa	7.450	2.870	3.860	120.0	71.6	35.80
DAC M2,<br>P02 (h1), 180 GPa	7.756	2.871	3.797	118.34	74.42	37.21
DAC M2,<br>P02 (h3), 185 GPa	7.529	2.888	3.806	118.34	72.87	36.43
DAC M2,	7.542	2.876	3.815	118.74	72.57	36.28

P03, 190-196 GPa						
DAC M2,<br>P04, 201-208 GPa	7.495	2.863	3.813	117.21	72.79	36.39
DAC M2,<br>P05, 208-210 GPa	7.482	2.854	3.790	117.74	71.66	35.83

Table S10. Calculated unit cell parameters of $C2/m$-SnH_{14} at different pressures.

Pressure, GPa	a, Å	b, Å	c, Å	(180 – β), °	V, Å^3	V, Å^3/Sn
160	7.628	2.940	3.907	61.56	77.07	38.53
180	7.534	2.901	3.866	61.38	74.19	37.09
200	7.445	2.866	3.828	61.27	71.66	35.83
210	7.406	2.850	3.812	61.17	70.51	35.25
220	7.368	2.834	3.796	61.11	69.43	34.71
230	7.334	2.820	3.779	61.05	68.41	34.21
240	7.297	2.805	3.766	61.00	67.44	33.72

Table S11. Calculated unit cell parameters of cubic SnH_2 (prototype is $Fm\bar{3}m$-YH_2) and SnH_3 (prototype is $Fm\bar{3}m$-YH_3).

Pressure, GPa	***Fm*-3*m*-Sn_4H_{12} (SnH_3)**			***Fm*-3*m*-Sn_4H_8 (SnH_2)**		
	a, Å (Z=4)	V, Å^3 (Z=4)	V, Å^3/Sn	a, Å (Z=4)	V, Å^3 (Z=4)	V, Å^3/Sn
160	4.232	75.79	18.95	4.188	73.43	18.36
180	4.186	73.34	18.33	4.139	70.95	17.74
200	4.146	71.26	17.81	4.098	68.82	17.20
210	4.126	70.26	17.56	4.078	67.81	16.95
220	4.107	69.26	17.32	4.058	66.85	16.71

Table S12. Calculated unit cell parameters of $Fm\bar{3}m$-SnH_5, $Fm\bar{3}m$-SnH_6, $Fm\bar{3}m$-SnH_7 (prototype is YMg_7), $Fm\bar{3}m$-SnH_8 (prototype is UH_8), $F\bar{4}3m$-SnH_9 (prototype is PrH_9) and $Fm\bar{3}m$-SnH_{10} (prototype is LaH_{10}).

Pressure, GPa	V, Å^3 /Sn (SnH_5)	V, Å^3 /Sn (SnH_6)	V, Å^3 /Sn (SnH_7)	V, Å^3 /Sn (SnH_8)	V, Å^3 /Sn (SnH_9)	V, Å^3 /Sn (SnH_{10})
180	21.50	26.54	27.00	26.32	-	-
200	20.86	25.68	26.17	25.56	26.93	28.52
210	20.56	25.38	25.82	25.20	26.54	28.10
220	20.30	25.01	25.45	24.84	26.17	27.70
230	-	-	-	-	25.80	27.32
240	-	-	-	-	25.47	26.94

4. Raman spectra

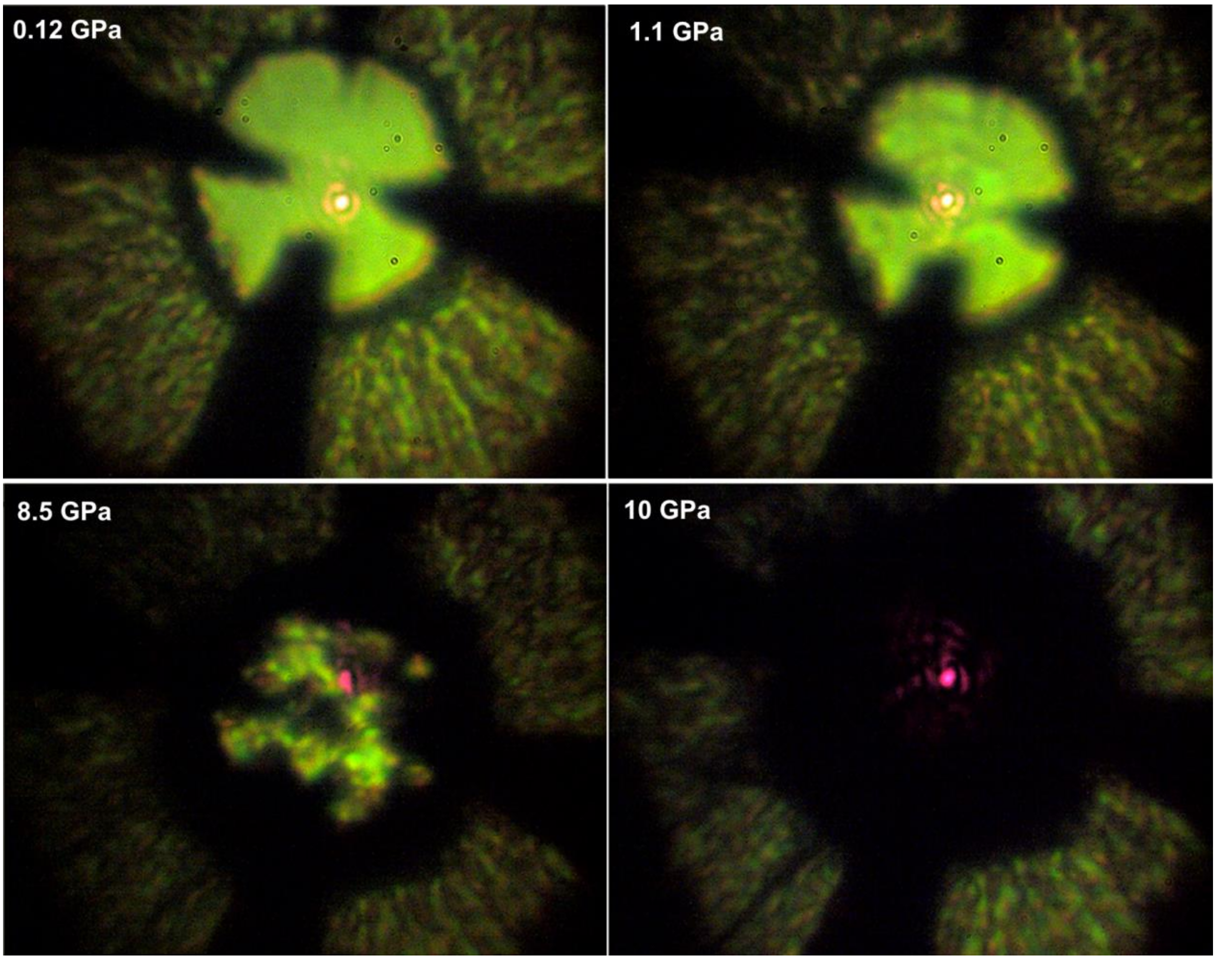

Figure S7. Metallization of molecular stannane compressed in a diamond anvil cell to 10 GPa. A cryogenic loading of the DAC was used.

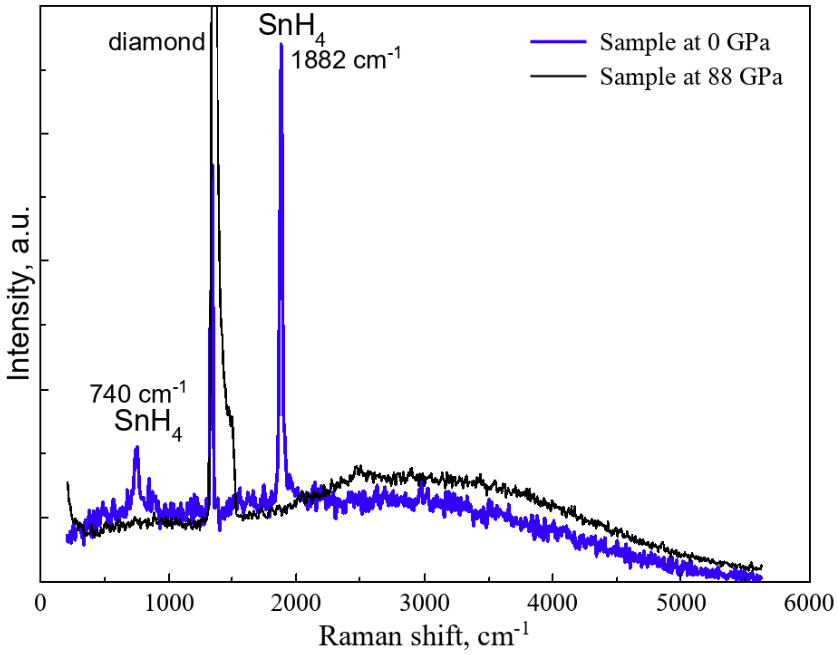

Figure S8. Raman spectrum of stannane (SnH_4) immediately after loading into the diamond cell (0 GPa, blue line) and after metallization (at 88 GPa, black line). The wavelength of the exciting laser is 633 nm.

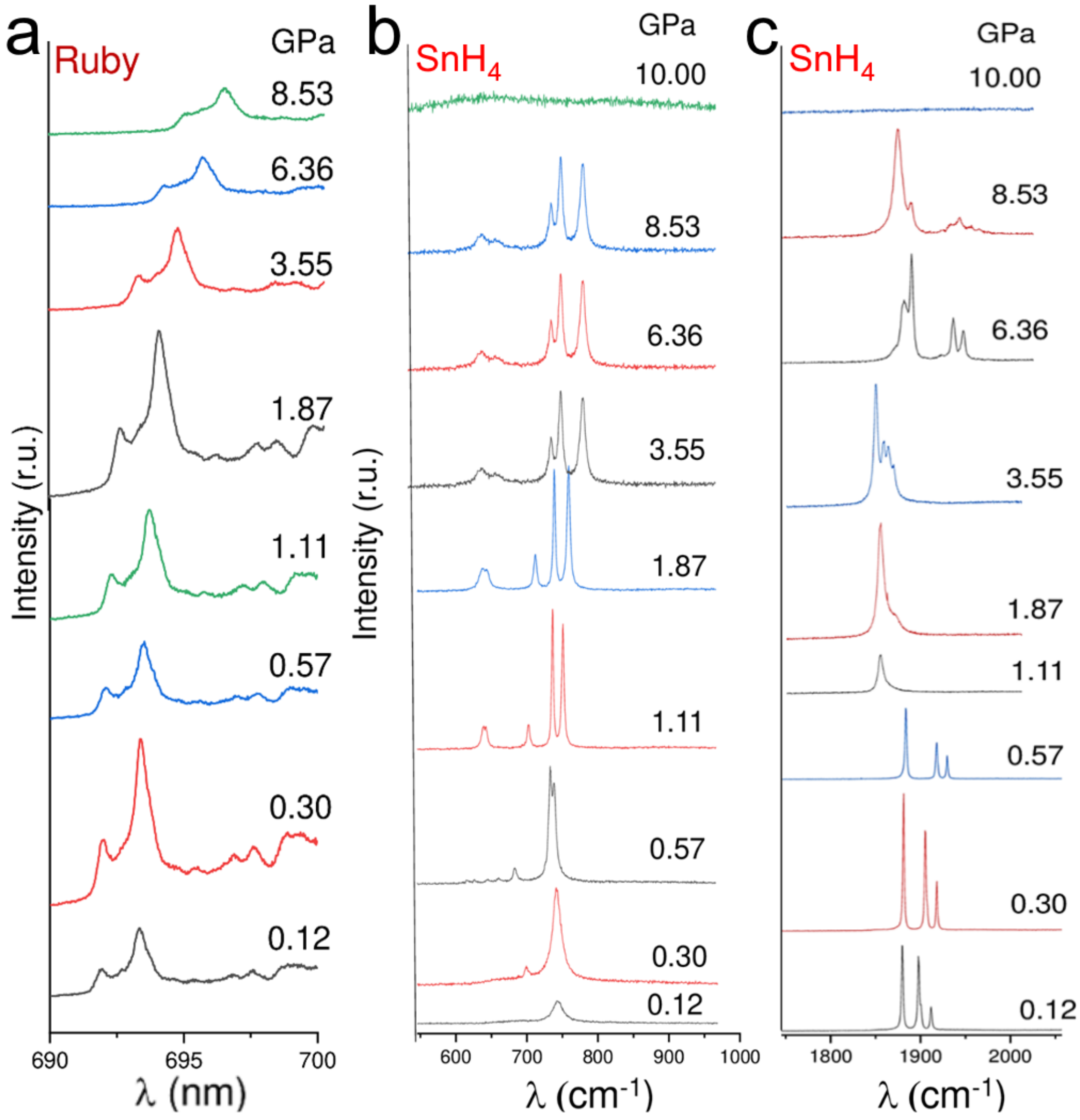

Figure S9. Raman spectra of stannane below 10 GPa measured at 157-164 K. (a) The ruby fluorescence peak and its changes with pressure. (b) Raman spectra of SnH_4 in 600-1000 cm^{-1} range at different pressures. There are signs of phase transitions in SnH_4 as the pressure increases. (c) Raman spectra of SnH_4 in 1700-2100 cm^{-1} range at different pressures. Above 10 GPa, the Raman peaks of stannane disappear. The wavelength of the exciting laser is 633 nm.

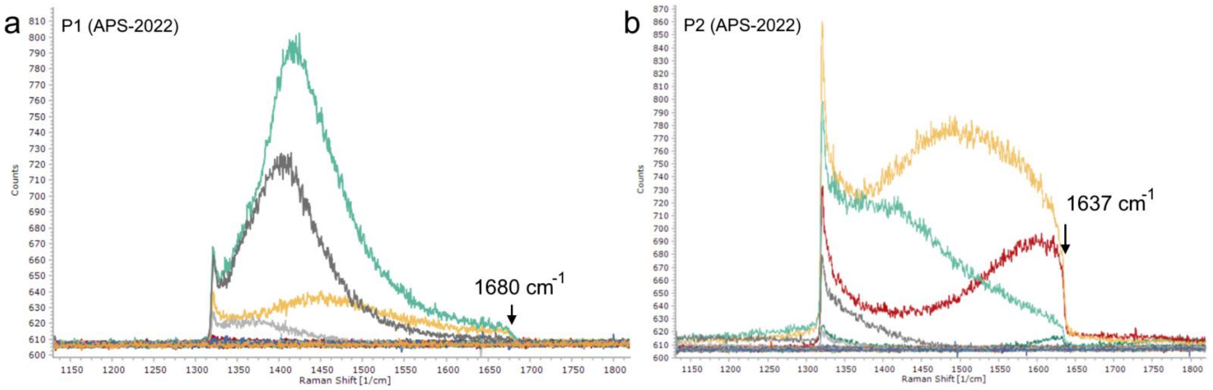

Figure S10. Raman spectra of the diamond anvils in the region of C-C vibrations, obtained at APS-2022. Different curves correspond to different points on the diamond culet.

5. Electron and phonon band structures of tin hydrides

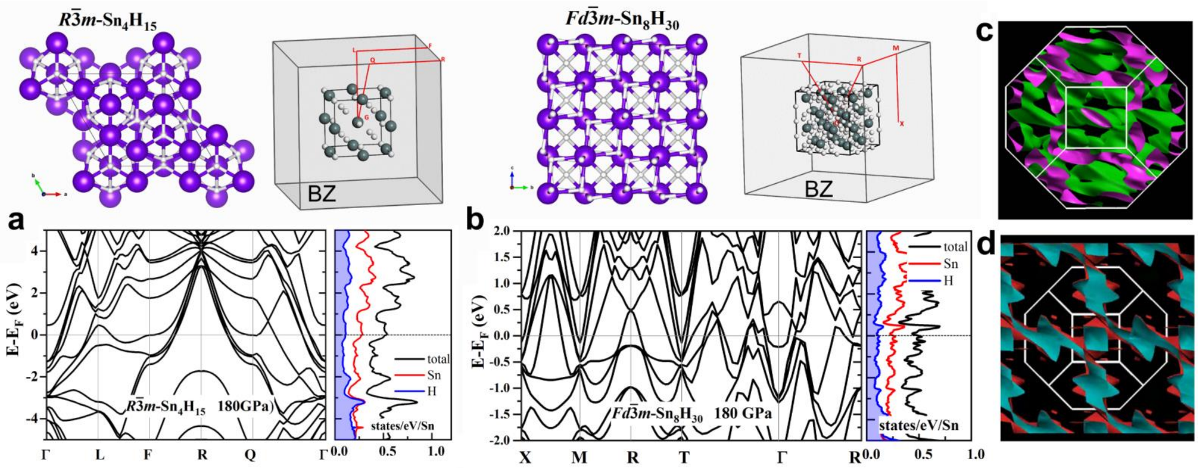

Figure S11. Crystal and electron band structures of (a) $R\bar{3}m$-$Sn_{12}H_{45}$ (Sn_4H_{15}) and (b) $Fd\bar{3}m$-Sn_8H_{30} calculated at 180 GPa (PAW PBE). (c, d) Sections of the Fermi surface of $Fd\bar{3}m$-Sn_8H_{30} showing its open character (XCrySDen). Bands correspond to E_{min} = 15.39 eV, E_{max} = 18.07 eV (c) and E_{min} = 16.36 eV, E_{max} = 18.11 eV (d) intervals.

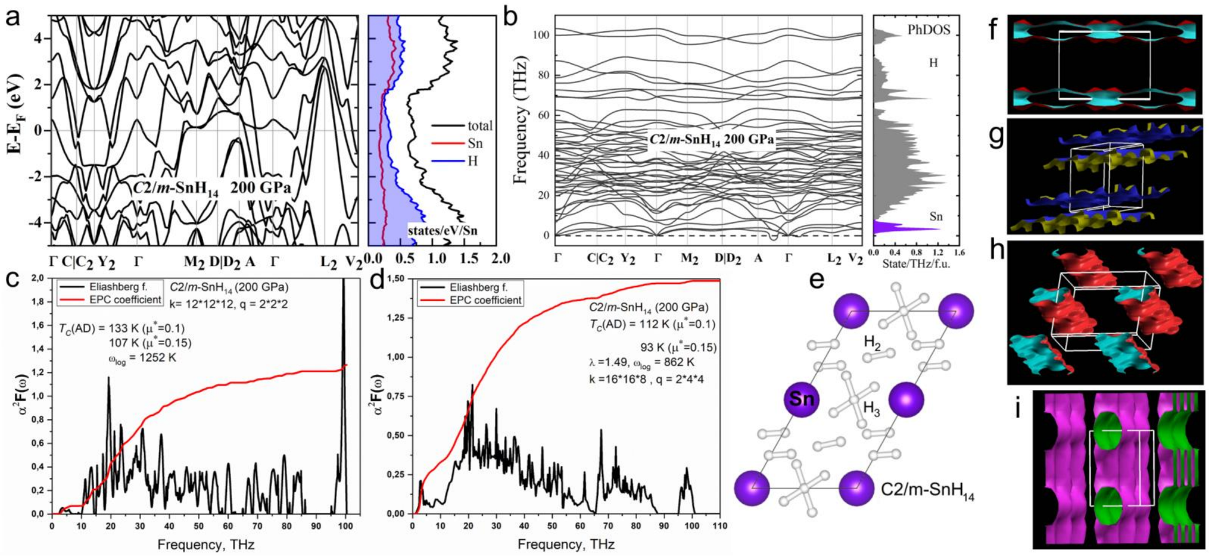

Figure S12. Electron (a), phonon (b) band structures and crystal structure (e), as well as Eliashberg functions (c, d) of $C2/m$-SnH_{14} at 200 GPa. Red lines in (c, d) correspond the dependence of the electron-phonon coupling coefficient on the frequency. (f-i) Sections of the Fermi surface of $C2/m$-SnH_{14} at 200 GPa showing its open character (XCrySDen).

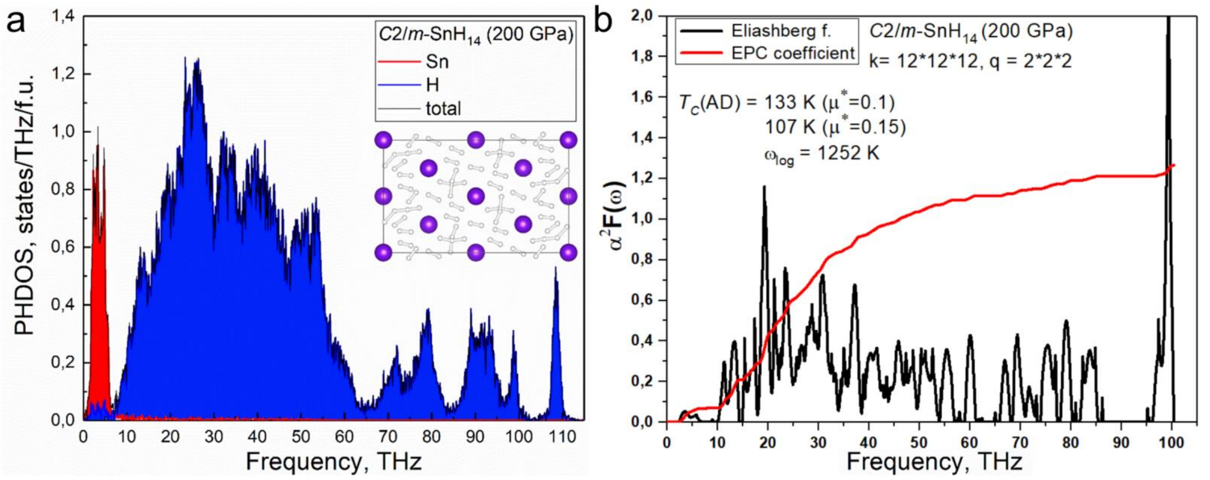

Figure S13. (a) Anharmonic density of phonon states at 200 GPa and 300 K obtained using molecular dynamics with MTP potentials, and (b) harmonic Eliashberg function with parameters of superconductivity of $C2/m$-SnH_{14}.

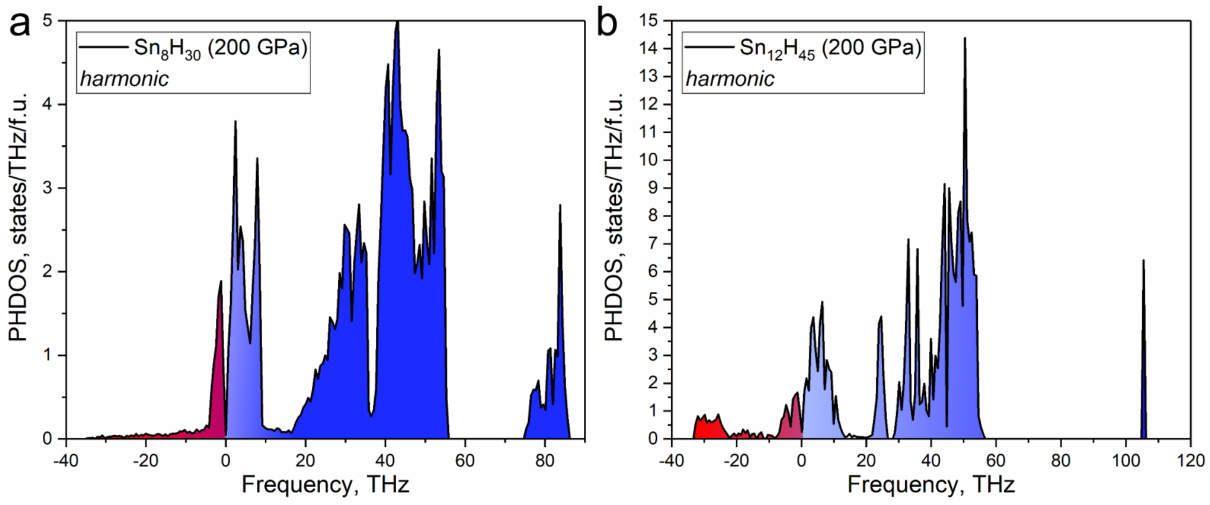

Figure S14. Harmonic density of phonon states of (a) $Fd\bar{3}m$-Sn_8H_{30} (f.u. = Sn_8H_{30}) and (b) $R\bar{3}m$-$Sn_{12}H_{45}$ (f.u. = $Sn_{12}H_{45}$) at 200 GPa and 0 K obtained using VASP [10-12] and Phonopy [13,14] codes. The imaginary part is marked in red.

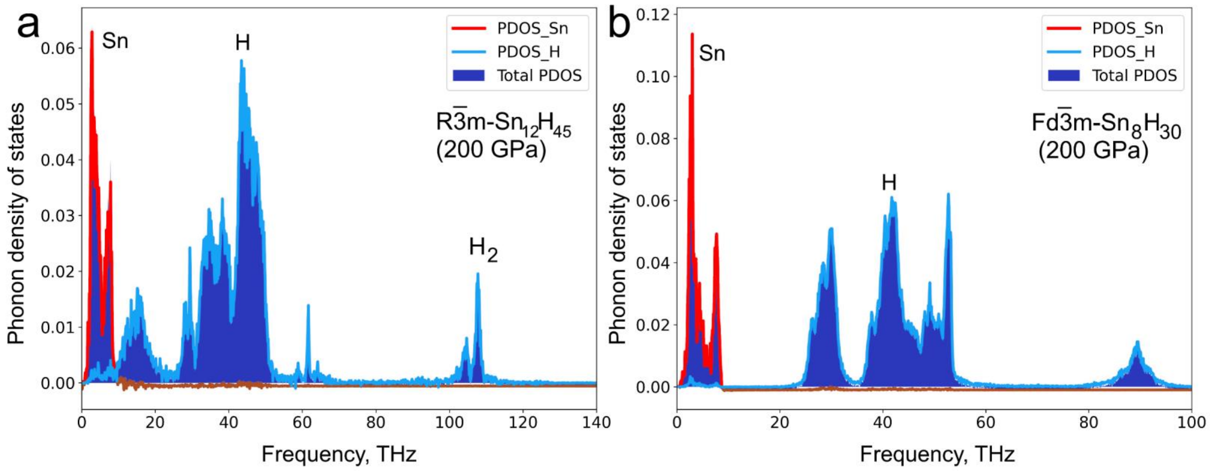

Figure S15. Anharmonic density of phonon states (integral of PDOS is normalized to 1, THz^{-1}) of (a) $R\bar{3}m$-$Sn_{12}H_{45}$ and (b) $Fd\bar{3}m$-Sn_8H_{30} at 200 GPa and 300 K obtained using molecular dynamics with MTP potentials.

6. Transport and superconducting properties

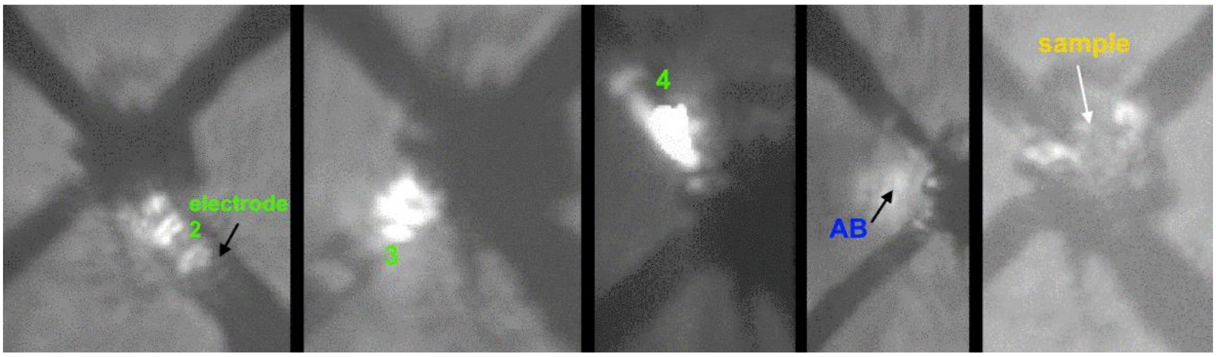

Figure S16. A series of optical photographs of a Sn hydride sample on a 50-μm diameter diamond anvil with four sputtered electrical contacts. Arrows and numbers show electrodes, ammonia borane (AB), and the sample.

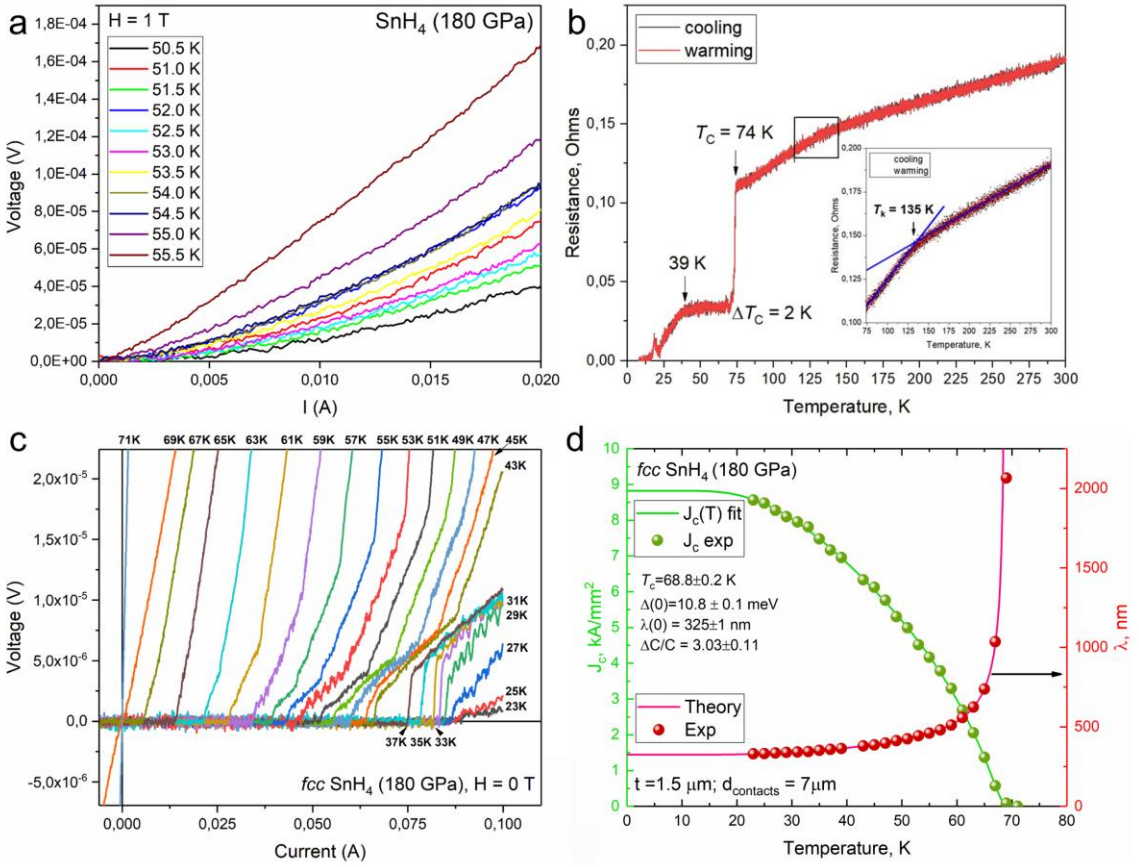

Figure S17. (a) Voltage-current (V-I) characteristic of the cubic SnH_4 sample at 180 GPa in a 1T magnetic field (H) in the temperature range of 50.5 - 55.5 K. The moment of transition to the superconducting state is weakly expressed probably due to heating of the sample (there is a quadratic ~T^2 component in V(I)). (b) Temperature dependence of the electrical resistance in a multiphase Sn hydride sample at 190 GPa (3rd laser heating). The inset shows a previously discovered anomaly (kink) at 135 K. Signs of a second, probable, superconducting transition are observed at ~39 K. (c) Voltage-current (V-I) characteristic of the cubic SnH_4 sample at 180 GPa in the absence of magnetic field in the temperature range of 23 - 71 K. (d) Temperature dependence of critical current density (green balls) and penetration depth (red balls) and fitting curves based on the results of Talantsev et al. [26,27]. For calculations, we used an interelectrode distance of 7 microns.

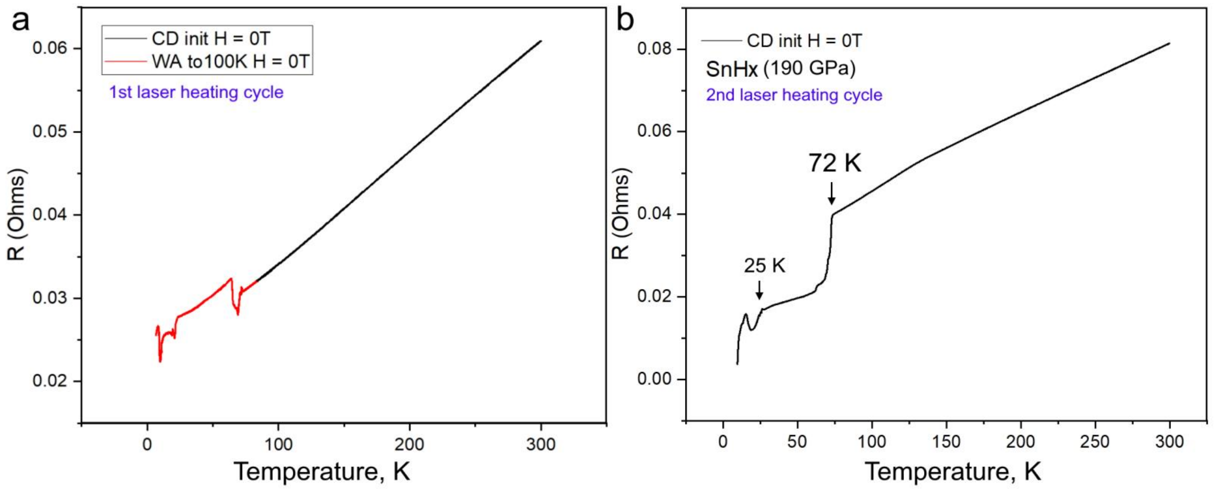

Figure S18. Temperature dependence of the electrical resistance for insufficiently heated Sn samples at 190 GPa. (a) The first cycle of laser heating. Only a local resistance anomaly is observed in the region of the expected superconducting transition (70 – 75 K). (b) The second cycle of laser heating. Due to an increase in the concentration of superconducting phase in the sample, a partial resistive transition at 72 K was observed. The third laser heating cycle results in the curve shown in Figure 17b.

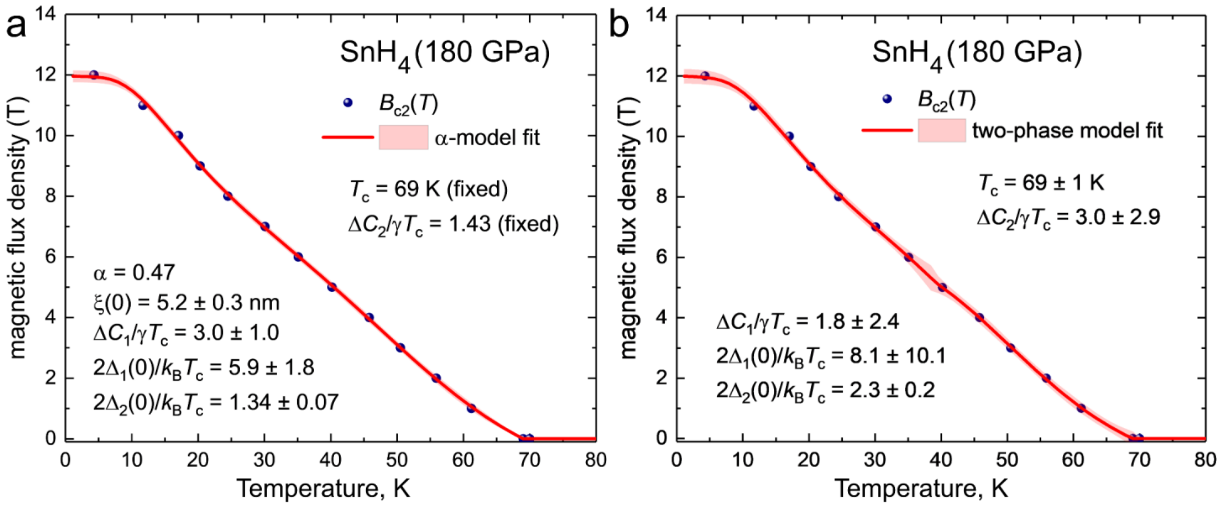

Figure S19. Possible explanation of the quasilinear dependence of the upper magnetic field on temperature B_{C2}(T) for cubic SnH_4 using two-gap models: (a) α-model, (b) two-phase model. The critical temperature T_C = 69 K is taken at the first point where *R(T)* < 0. The analysis was carried out by Dr. Evgueni Talantsev (M.N. Mikheev Institute of Metal Physics, Ural Branch of RAS).

Estimation of the upper critical magnetic field of $Fd\bar{3}m$-Sn_8H_{30} in the clean limit by a simplified model for hydrides, suggested in Ref. [42], gives:

$$B_{C2}^{cl}(0) = 2.45 \times 10^{-4} T_C^2 (1+\lambda)^2, \qquad \text{(S7)}$$

where T_C = 72 K, and λ is the electron-phonon interaction parameter which varies from 1.24 (harmonic approximation) to 2.98 (anharmonic approximation). Depending on the λ value, Eq. (4) gives $B_{C2}(0)$ = 6.4 – 20.3 T, which is in acceptable agreement with the experimental data (14-16 T). From eq. (S7), considering the anomalously linear dependence *R(T),* it follows that the value of the electron-phonon interaction parameter in Sn tetrahydride is quite large (λ ~ 2.5).

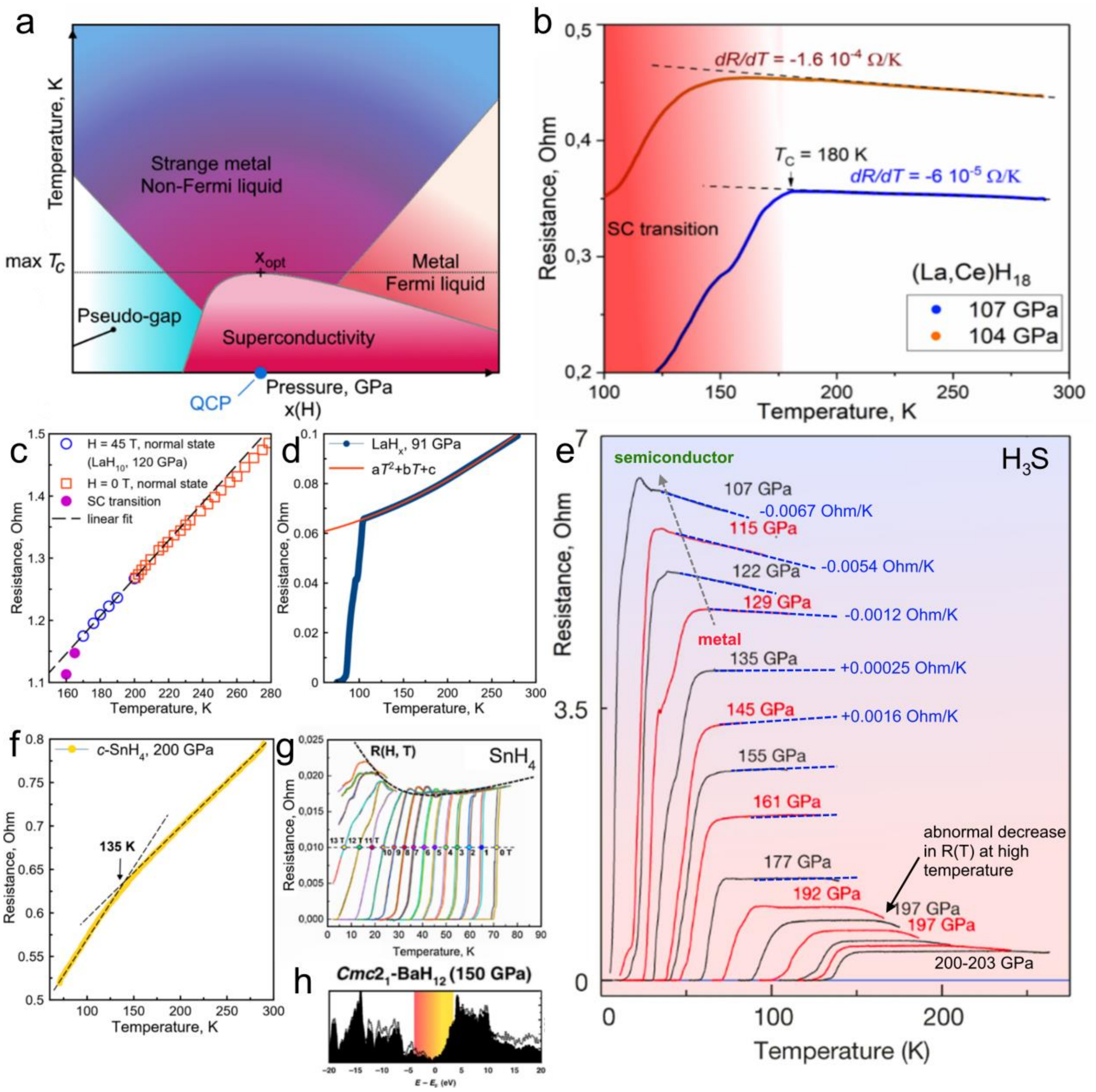

Figure S20. Examples of anomalous behavior of superhydrides in the normal resistivity state and their hypothetical phase diagram. (a) Qualitative *P-T* phase diagram of compressed hydrides, constructed by analogy with cuprates. Similar to doping level in cuprates, pressure (P) plays the same role in hydrides. (b) Anomalous linear decrease in electrical resistance ($d\rho/dT < 0$) with increasing temperature in (La, Ce)H_9 [43]. (c) Anomalous temperature dependence (close to T-linear) of electrical resistance in LaH_{10}, observed at 120 GPa after suppression of the superconducting state by a magnetic field of 45 T [44]. (d) The Fermi liquid behavior of *R(T)* in LaH_x at 91 GPa. The electrical resistance can be approximated by a quadratic function [43]. (e) Gradual transition of sulfur hydride (H_3S) from a metallic (non-Fermi liquid) phase to a semiconducting pseudo-gap phase (below 135 GPa) with the inversion of sign of the temperature coefficient of resistance and the disappearance of superconductivity below 107 GPa [45]. (f) Anomalous reproducible kink of *R(T)* in *fcc* SnH_4 which cannot be explained in terms of the Fermi liquid model. (g) Growth of electrical resistance of cubic SnH_4 at low temperatures after suppression of superconductivity by magnetic field. This is a probable manifestation of pseudo-gap phase. (h) Pronounced pseudo-gap in the electronic structure of a superconducting molecular $Cmc2_1$-BaH_{12} at 150 GPa [46].

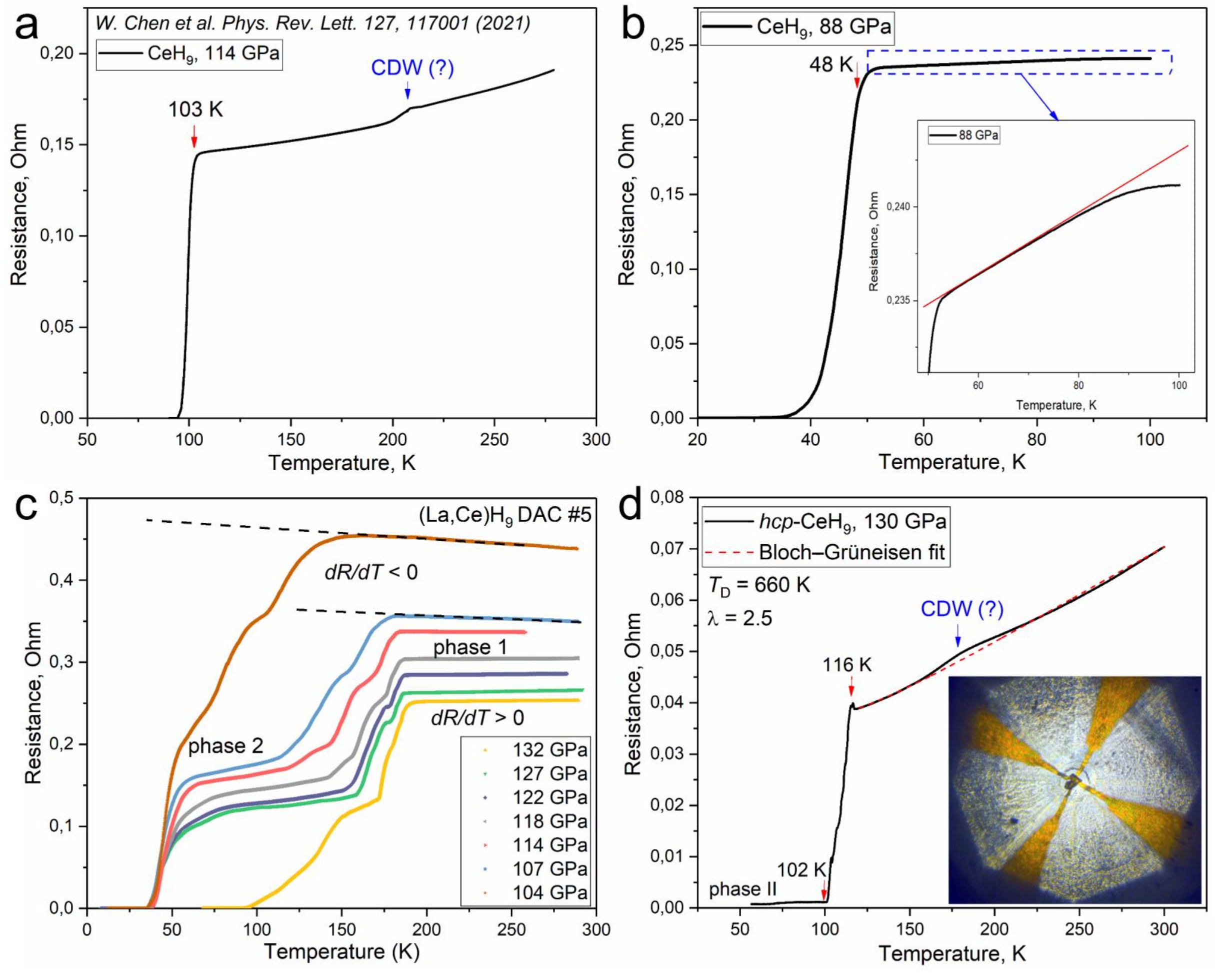

Figure S21. Examples of anomalous behavior of electrical resistance in samples of cerium and lanthanum-cerium hydrides. (a) Temperature dependence *R(T)* in CeH_9 at 114 GPa, (b) the same dependence at 88 GPa [47]. In both cases, *R(T)* cannot be interpolated using the Bloch–Grüneisen formula [48,49]. (c) Reversal of the sign of the temperature coefficient of electrical resistance during decompression of $(La,Ce)H_9$ [43]. A similar effect is observed in *bcc* H_3S with decreasing pressure [45]. (d) Reproducible anomaly of the *R(T)* dependence in $P6_3/mmc$-CeH_9. This experiment was carried out in Moscow (IC RAS) in 2022. CDW means the charge density wave, which is one of the possible reasons of such anomalies in CeH_9 at high pressure.

Table S13. Parameters of the normal resistivity state of the cubic SnH_4 found in the study of the magnetoresistance. The mobility of electrons and their relaxation time were calculated from the quadratic part of the ρ(*B*) dependence and, in order of magnitude, they correspond to the parameters of metals (e.g., Sr, Ba, Pb…) [50].

Temperature, K	Crit. magnetic field (B_{cr}), T	Cyclotron time (τ_c), s^{-1}	Electron mobility ($\rho=\mu^2B^2$), $m^2/s\times V$	Electron relaxation time, ($\tau = \mu\times m_e/e$), s^{-1}
75*	5	1.1e-12	0.033	1.9e-13
80	6	9.5e-13	0.035	2e-13
100*	7.5	7.6e-13	0.022	1.25e-13
145.5	12	4.7e-13	0.017	9.45e-14
216	14	4e-13	0.015	8.53e-14

*PPMS measurements

It should also be noted that the linear behavior of the magnetoresistance (MR) is also typical for polycrystalline metals with an open Fermi surface, for example, for Li, Cu, Ag, Au, etc. [51]. This behavior was explained in 1959 by Lifshits and Peschanskii [52], who took into account the shape of the Fermi surface and the anisotropy of MR. When averaged over all directions ($\Delta\theta \sim H_0/H$) and open sections of the Fermi surface (where $\rho \sim H^2$) for polycrystals, the MR acquires a linear dependence on the magnetic field [52]. The open topology of the Fermi surface is confirmed by DFT calculations (Supporting Figures S11-S12).

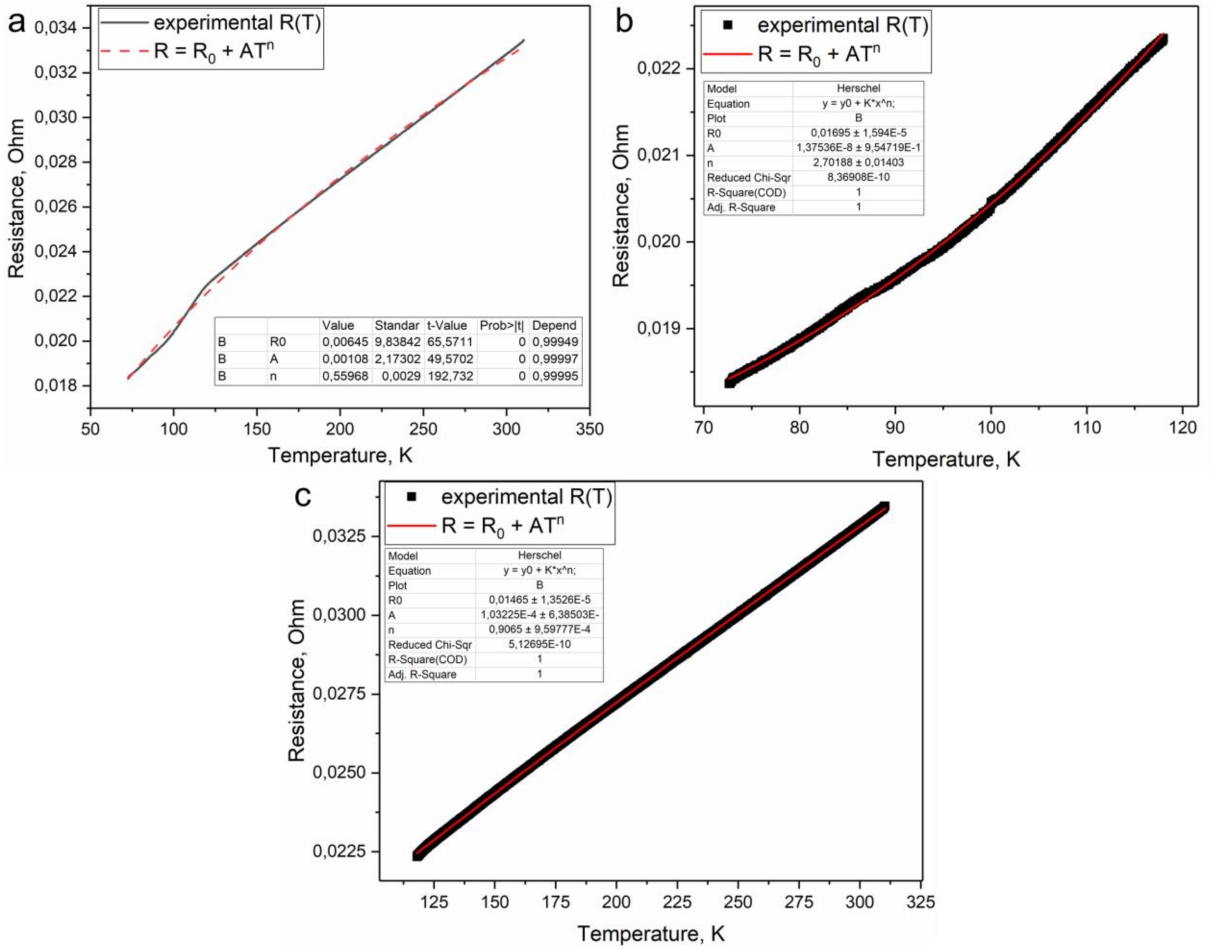

Figure S22. Interpolation of experimental data for the dependence of electrical resistance on the temperature of the SnH_4 sample at 180 GPa. We used a power law fit $R(T) = R_0 + A\times T^n$. (a) Interpolation over the entire temperature range, $n \approx 0.56$, the interpolation quality is poor due to the presence of an inflection point near 118 K. (b) Interpolation at $T < 118$ K (inflection point), $n \approx 2.7$. (c) Interpolation above the inflection point, $T > 118$ K, $n \approx 0.9$.

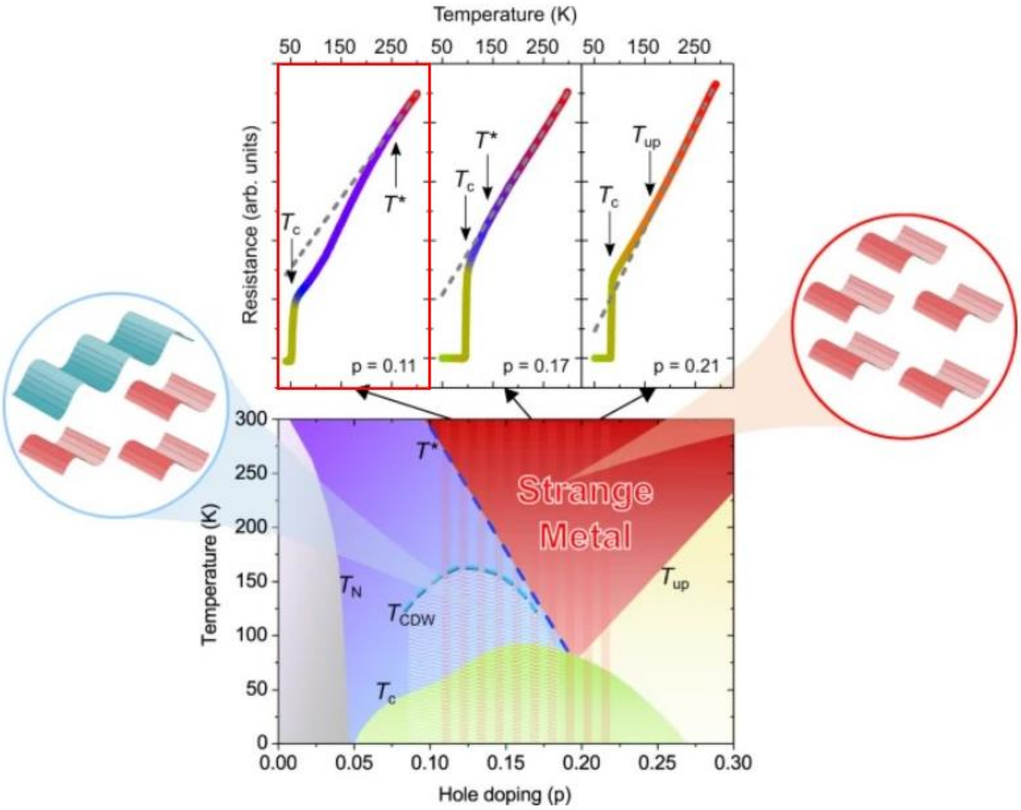

Figure S23. Figure 1 from Seibold, G. et al. *Commun Phys* 4, 7 (2021) with a characteristic feature of the dependence of electrical resistance R(T) on temperature (red frame, p = 0.11), which is explained by the boundary between the area of charge density waves and strange metal phase. Similar behavior is found in tin hydride SnH_4 at T^* = 120-135 K.

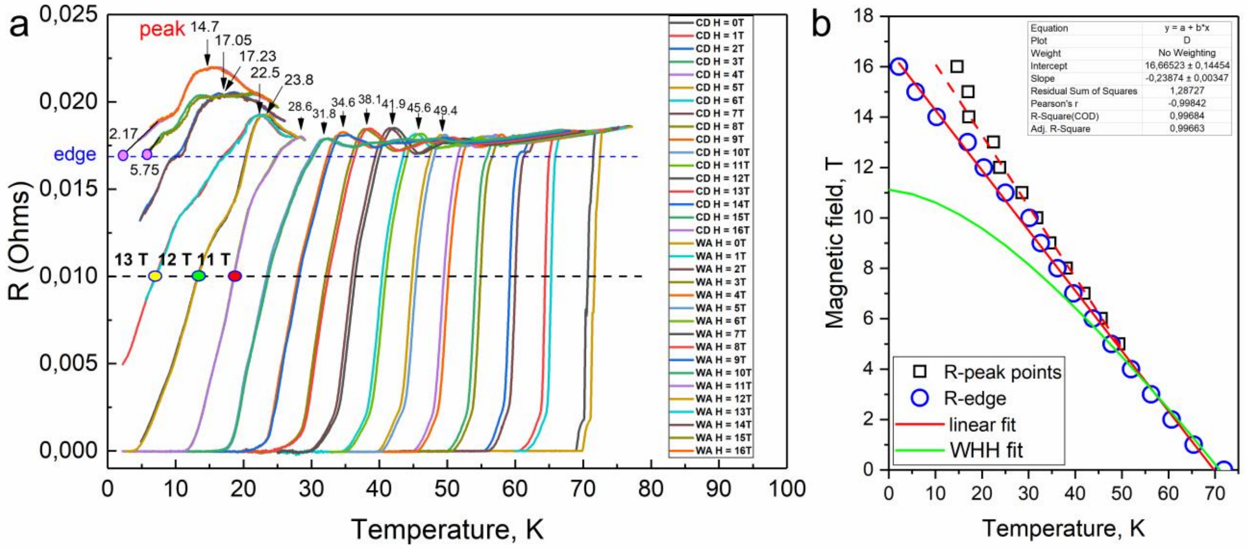

Figure S24. Analysis of the temperature dependence of the upper critical field of SnH_4 based on the use of the maximum R(T) values near T_C (peak value, "peak") and the use of the line $R_{edge} \leq R(T)$ in the non-superconducting state for all studied magnetic field values. (a) Dependence of the electrical resistance of SnH_4 on temperature and magnetic field. (b) Magnetic phase diagram SnH_4, linear and the WHH fits for critical temperatures calculated from the peak resistance value and from the "edge" resistance limit. The deviation from the WHH model is very pronounced.

Holes and electrons mobility in the Sn_8H_{30} structure at 180 GPa were estimated by the aMoBT code [53] using the LDA exchange-correlational functional with k-mesh 8×8×8. Dielectric constant at high frequencies was taken $\varepsilon_\infty = 6$, and at low frequencies $\varepsilon_0 = 56$ on the basis of DFT calculations of the dielectric function. Imaginary part was not considered. Single band approximation was used. Polar phonon frequency was set 40 THz on the basis of the phonon density of states at this pressure. Deformation potential was chosen as 5 eV. We used piezoelectric coefficient P = 0.15 and maximum possible in aMoBT carrier concentration of 2×10^{20} cm^{-3}. Results of calculations are given in Table S14.

Table S14. Estimates of the mobility of electrons and holes in Sn_8H_{30} at 180 GPa.

Temperature, K	Electron average effective mass, m^*/m_e	Electron average mobility, $cm^2/V\times s$	Hole average effective mass, m^*/m_e	Hole average mobility, $cm^2/V\times s$	$\mu_h/\ \mu_e$
100	2.03	32	0.80	766	23.8
300	2.03	6.0	0.80	395	66

7. Eliashberg functions

Calculations of the anharmonic correction to the critical temperature of superconductivity in this work are based on the constant DOS approximation [54], which is applicable if the density of electronic states N(E) in the vicinity of the Fermi energy has no pronounced features (e.g., van Hove singularities [55]) and can be approximately represented as a constant. In this case, the main contribution to the shape of Eliashberg function is made by the phonon density of states (denoted here for convenience as $F(\omega)$), and an auxiliary function of electron contribution $\alpha^2(\omega) = \alpha^2F(\omega)/F(\omega)$ is close to a constant. The exceptions are those frequency zones where both functions $\alpha^2F(\omega)$ and $F(\omega) \sim 0$, and where the calculation of their ratio is not sufficiently stable and accurate.

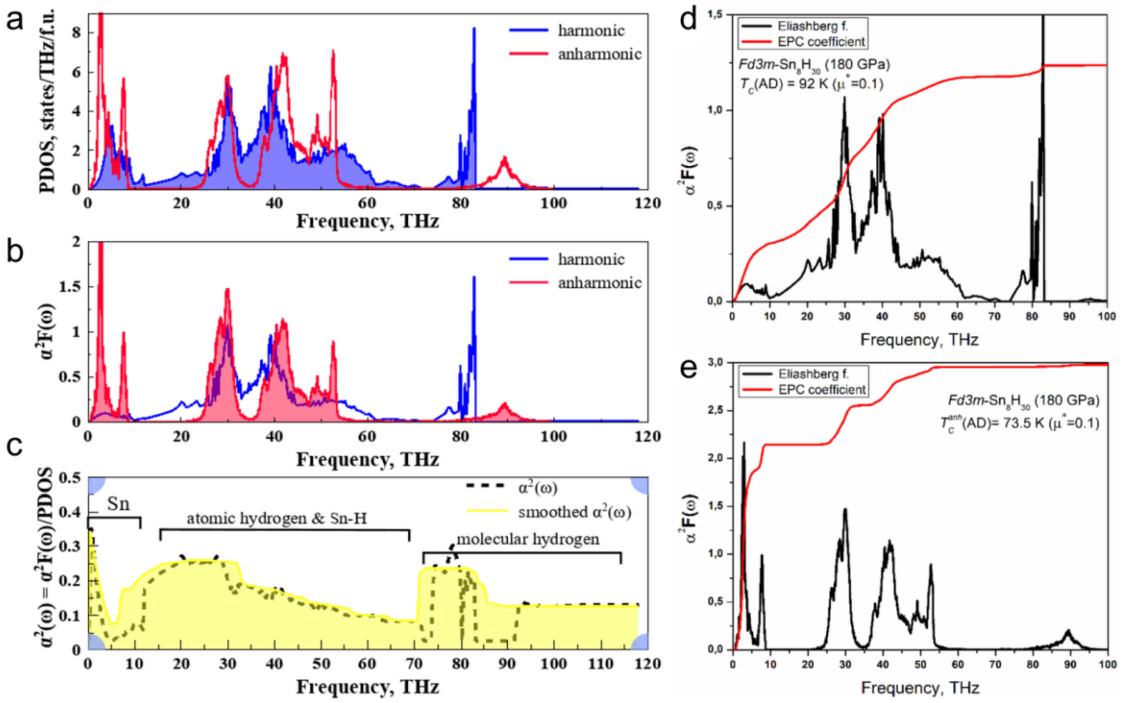

Figure S25. Harmonic and anharmonic spectral functions of $Fd\bar{3}m$-Sn_8H_{30} at 180 GPa, f.u. = Sn_8H_{30}. (a) Harmonic (blue) and anharmonic (red) density of phonon states at 100 K. (b) Harmonic (blue) and anharmonic (red) Eliashberg functions. (c) Smoothed (yellow) and original (black dotted line) function of contribution of the density of electronic states (formally, $\alpha^2(\omega)$). (d) Superconducting state parameters of $Fd\bar{3}m$-Sn_8H_{30} at 180 GPa in harmonic (d) and anharmonic (e) approximations. Attention is drawn to the presence of high-intensity soft modes in molecular dynamics calculations of Sn_8H_{30} which can lead to the emergence of the charge density waves (CDW).

In this work, the procedure for calculating the Eliashberg anharmonic functions of Sn hydrides consisted of several steps:

1. Calculation of the harmonic Eliashberg function and phonon density of states in Quantum ESPRESSO [17,18] using the tetrahedral method [56].

2. Calculation of the anharmonic phonon spectrum using the molecular dynamics [57] at a temperature near the superconducting transition (T_C). Here it is a temperature of 100 K.

3. Equalization of unit cell (N - is number of atoms in the formula unit) by calibration of integrals

$$\int F_{harm}(\omega)d\omega = \int F_{anh}(\omega)d\omega = 3N \qquad \text{(S8)}$$

4. Calculation of the contribution of the electron density of states to the Eliashberg function for the harmonic case: $\alpha^2(\omega) = \alpha^2F(\omega)/F(\omega)$.

5. Reduction of the function $\alpha^2(\omega) \geq 0$ to the same mesh on the energy scale that was used to calculate the anharmonic phonon spectrum by linear interpolation. All obtained negative values $\alpha^2(\omega) < 0$ were set to zero.

6. Smoothing the obtained $\alpha^2(\omega)$ to eliminate the influence of "empty" frequency zones where both $\alpha^2F(\omega)$ and $F(\omega) \sim 0$. In this work, we used the percentile filter (points of window is 140, percentile is 70) in the OriginLab 2017 [58] program.

7. Calculation of the Eliashberg anharmonic function as the product of the electronic contribution and the anharmonic density of phonon states $\alpha^2(\omega)F_{anh}(\omega)$.

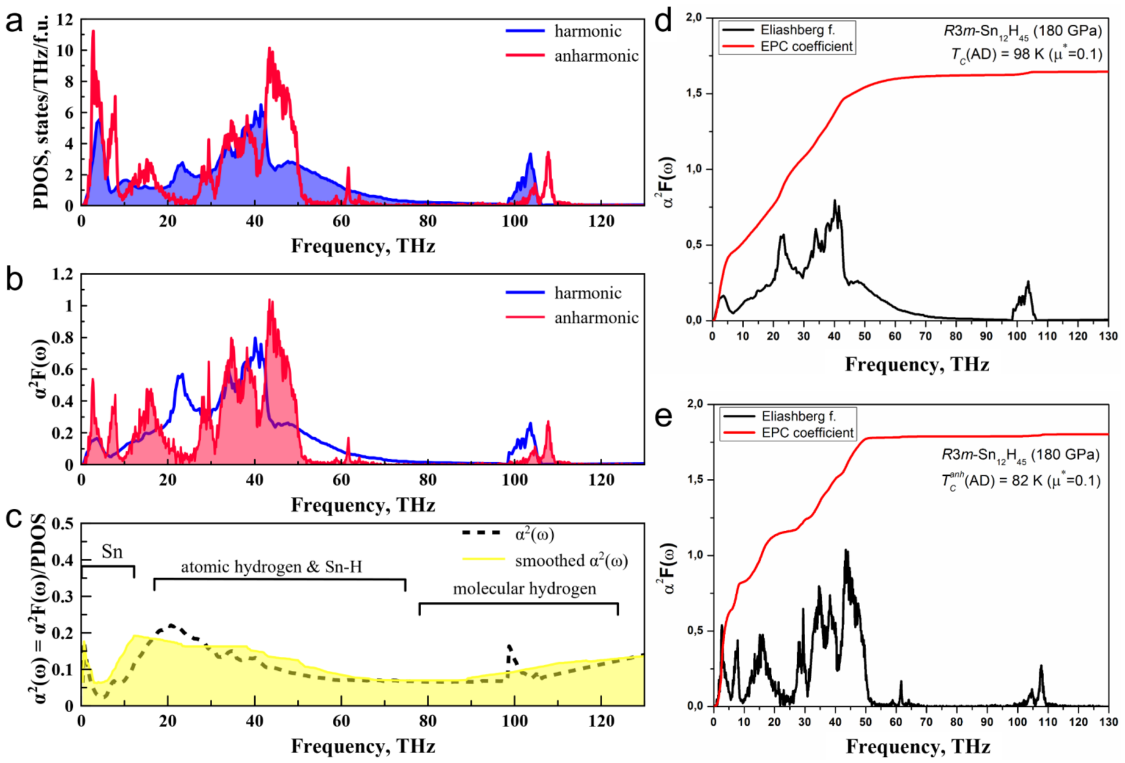

Figure S26. Harmonic and anharmonic spectral functions of $R\bar{3}m$-$Sn_{12}H_{45}$ at 180 GPa, f.u. = $Sn_{12}H_{45}$. (a) Harmonic (blue) and anharmonic (red) density of phonon states at 100 K. (b) Harmonic (blue) and anharmonic (red) Eliashberg functions. (c) Smoothed (yellow) and original (black dotted line) function of contribution of the density of electronic states (formally, $\alpha^2(\omega)$). (d) Superconducting state parameters of $R\bar{3}m$-$Sn_{12}H_{45}$ at 180 GPa in harmonic (d) and anharmonic (e) approximations.

Some details of the obtained results require discussion. First, the significant growth of the electron-phonon coupling (EPC) coefficient λ from 1.24 to $\approx$ 3 in the anharmonic approximation attracts attention. The opposite effect is usually observed, which is associated with acoustic phonons hardening [59]. However, in our case, within the harmonic approximation, Sn_8H_{30} is not dynamically stable at 180-200 GPa, and has a series of imaginary acoustic modes that we did not take into account in our calculations of the superconducting state (see Figure S14). Considering anharmonic corrections, these imaginary modes, roughly speaking, move to the real acoustic part of the phonon spectrum, and cause a sharp maximum in $F(\omega)$ around 0-7 THz (Figures S15, S25), as well as lead to an increase in the EPC coefficient.

The intermediate range of frequencies, that corresponds to the atomic hydrogen vibrations (10-60 THz), is subjected to the aforementioned phonons hardening. As a result, the 10-25 THz

frequency zone becomes almost empty, and the modes, that were previously in this region, cause a significant increase in the intensity of the $\alpha^2F(\omega)$ and $F(\omega)$ functions in the range 25-55 THz (Figures S25a, b).

In the range of molecular hydrogen vibrations, anharmonic interactions of H_2 molecules with the framework of the atomic H-sublattice and with Sn atoms lead to the splitting and smoothing of the phonon density of states in the range of 75-95 THz (Figure S25a, b). There is also a strong high-frequency shift of these modes, which is characteristic of the anharmonic approximation (for example, H_3S [60], high-frequency region).

Thus, despite the pronounced phonons hardening for hydrogen modes, the effect of soft modes of Sn atoms (compare with *hcp*-NdH_9 [61]) leads to an unexpected increase in the electron-phonon interaction strength by more than two times and a sharp decrease in the logarithmically averaged frequency (ω_{log}) of Sn_8H_{30} at 180 GPa. As a result, the critical temperature (T_C) is reduced by about 20 K compared to harmonic calculations, which is in qualitative agreement with the results obtained for other hydrides (e.g. AlH_3 [62], YH_6 [16,63], LaH_{10} [64,65], H_3S [45]).

Summing up, we can say that the properties of cubic SnH_4 are unusual: this compound exhibits much higher electron-phonon interaction strength (λ = 2.5 – 3) than one would expect from a hydride with such a low hydrogen content. SnH_4 can rather be described as a covalent superconductor, unlike lanthanum (LaH_{10}) and yttrium (YH_6) superhydrides.

Table S15. Superconducting properties of $Fd\bar{3}m$-Sn_8H_{30} at 180 GPa. AD – corresponds to calculations made by Allen-Dynes formula [20]. Calculations of elastic properties give the following estimates: T_θ = 380 K, $\omega_{log} \approx$ 315 K (see below).

**Parameter**	**λ**	**ω_{log}, K**	**ω_2, K**	**T_C(AD), K (μ*=0.1)**
Harmonic*	1.24	892	1664	92.3
Anharmonic	2.98	282	1016	73.5

*Coherence length in this case is about 5 nm, the Ginzburg-Landau parameter is κ = 43, and the expected lower critical field is H_{C1} = 9 mT.

Table S16. Superconducting properties of *C*2/*m*-SnH_{14} at 200 GPa. AD – corresponds to calculations made by Allen-Dynes formula [20]. Calculations of elastic properties give the following estimates: T_θ = 1404 K, $\omega_{log} \approx$ 1160 K (see below).

**Parameter**	**k-mesh**	**q-mesh**	**λ**	**ω_{log}, K**	**ω_2, K**	**T_C(AD), K (μ*=0.15-0.1)**
Harmonic	12×12×12	2×2×2	1.27	1252	1930	107-133
Anharmonic	16×16×8	2×4×4	1.49	862	1585	93-112

Table S17. Critical temperatures of superconductivity for various tin polyhydrides predicted in previous years. It can be concluded that, despite the incorrect structures, the theoretical estimates of T_C are not very far from the experimental T_C(SnH_4).

**Phase**	**Pressure, GPa**	**T_C, K**	**Consistency (±20 %) with the exp T_C**	**Reference**
Ama2-SnH_4	200	15-22	No	G. Gao et al. [39]
*P*6$_3$/*mmc*-SnH_4	200	52-62	No	G. Gao et al [39]
*I*4/*mmm*-SnH_4	220	80-91	Yes	M. Davari et al.[38]

*I*4*m*2-SnH_8	220	72-81	Yes	M. Davari et al.[38]

8. Elastic properties

The elastic constants of tin hydrides were evaluated using VASP code (with IBRION=6, ISIF=4) [10-12]. The kinetic energy cutoff for plane waves was 600 eV. Tetrahedron method with Blöchl corrections was used [66]. The purpose of the performed calculations is to get an estimate for the Debye temperature, and to confirm the conclusions about the "softness" of Sn_8H_{30} obtained earlier on the basis of experimental data.

Table S18. Elastic properties of tin hydrides in harmonic approximation: $Fd\bar{3}m$-Sn_8H_{30} and *C*2/*m*-SnH_{14}.

Compound	C_{11}-C_{12}, GPa	C_{11}+2C_{12}, GPa	C_{44}, GPa	B, GPa	G, GPa	E, GPa	Poisson's ratio
Sn_8H_{30} (180 GPa)	179.1	1257.3	20.8	467.9	33.3*	97.5*	0.46
SnH_{14} (200 GPa)	409.5	1067.1	190.6	408.9	196.4	507.6	0.29

* Rather low values for a pressure of 200 GPa, characterizing unusual "softness" of this hydride.

Table S19. Elastic tensor C_{ij} (in kbar) of $Fd\bar{3}m$-Sn_8H_{30} at 180 GPa in harmonic approximation. The Debye temperature is 380 K [67].

	C_{i1}	C_{i2}	C_{i3}	C_{i4}	C_{i5}	C_{i6}
C_{1j}	5385.0973	3593.9683	4998.1048	19.781	53.2054	-67.0337
C_{2j}	3593.9683	6194.2358	4768.1404	19.276	-218.8843	4.2065
C_{3j}	4998.1048	4768.1404	3706.2409	91.0908	169.6859	328.4853
C_{4j}	19.781	19.276	91.0908	207.8296	59.2744	134.4097
C_{5j}	53.2054	-218.8843	169.6859	59.2744	496.8327	-14.6745
C_{6j}	-67.0337	4.2065	328.4853	134.4097	-14.6745	222.0507

Table S20. Elastic tensor C_{ij} (in kbar) of *C2/m*-SnH_{14} at 200 GPa in harmonic approximation. The Debye temperature is 1404 K [67].

	C_{i1}	C_{i2}	C_{i3}	C_{i4}	C_{i5}	C_{i6}
C_{1j}	6286.7031	2192.0071	3813.1104	0.0	0.0	-71.3417
C_{2j}	2192.0071	8677.1942	1284.712	0.0	0.0	384.5683
C_{3j}	3813.1104	1284.712	7304.6125	0.0	0.0	117.3508
C_{4j}	0.0	0.0	0.0	1906.2537	-357.1259	0.0
C_{5j}	0.0	0.0	0.0	-357.1259	1359.3217	0.0
C_{6j}	-71.3417	384.5683	117.3508	0.0	0.0	2313.0626

References

[1] A. R. Oganov, C. W. Glass, *J. Chem. Phys.* **2006**, *124*, 244704.
[2] A. R. Oganov, R. O. Lyakhov,M. Valle, *Acc. Chem. Res.* **2011**, *44*, 227-237.
[3] A. O. Lyakhov, A. R. Oganov, H. T. Stokes,Q. Zhu, *Comput. Phys. Commun.* **2013**, *184*, 1172-1182.
[4] P. V. Bushlanov, V. A. Blatov,A. R. Oganov, *Comput. Phys. Commun.* **2019**, *236*, 1-7.
[5] P. Hohenberg, W. Kohn, *Phys. Rev.* **1964**, *136*, B864-B871.
[6] W. Kohn, L. J. Sham, *Phys. Rev.* **1965**, *140*, A1133-A1138.
[7] J. P. Perdew, K. Burke,M. Ernzerhof, *Phys. Rev. Lett.* **1996**, *77*, 3865-3868.
[8] G. Kresse, D. Joubert, *Phys. Rev. B* **1999**, *59*, 1758-1775.
[9] P. E. Blöchl, *Phys. Rev. B* **1994**, *50*, 17953-17979.
[10] G. Kresse, J. Furthmüller, *Phys. Rev. B* **1996**, *54*, 11169.
[11] G. Kresse, J. Hafner, *Phys. Rev. B* **1994**, *49*, 14251-14269.
[12] G. Kresse, J. Hafner, *Phys. Rev. B* **1993**, *47*, 558-561.
[13] A. Togo, I. Tanaka, *Scr. Mater.* **2015**, *108*, 1-5.
[14] A. Togo, F. Oba,I. Tanaka, *Phys. Rev. B* **2008**, *78*, 134106.
[15] D. V. Semenok, I. A. Troyan, A. G. Kvashnin, A. G. Ivanova, M. Hanfland, A. V. Sadakov, O. A. Sobolevskiy, K. S. Pervakov, A. G. Gavriliuk, I. S. Lyubutin, K. Glazyrin, N. Giordano, D. Karimov, A. Vasiliev, R. Akashi, V. M. Pudalov,A. R. Oganov, *Mater. Today* **2021**, *48*, 18-28.
[16] I. A. Troyan, D. V. Semenok, A. G. Kvashnin, A. V. Sadakov, O. A. Sobolevskiy, V. M. Pudalov, A. G. Ivanova, V. B. Prakapenka, E. Greenberg, A. G. Gavriliuk, I. S. Lyubutin, V. V. Struzhkin, A. Bergara, I. Errea, R. Bianco, M. Calandra, F. Mauri, L. Monacelli, R. Akashi,A. R. Oganov, *Adv. Mater.* **2021**, *33*, 2006832.
[17] P. Giannozzi, S. Baroni, N. Bonini, M. Calandra, R. Car, C. Cavazzoni, D. Ceresoli, G. L. Chiarotti, M. Cococcioni, I. Dabo, A. D. Corso, S. d. Gironcoli, S. Fabris, G. Fratesi, R. Gebauer, U. Gerstmann, C. Gougoussis, A. Kokalj, M. Lazzeri, L. Martin-Samos, N. Marzari, F. Mauri, R. Mazzarello, S. Paolini, A. Pasquarello, L. Paulatto, C. Sbraccia, S. Scandolo, G. Sclauzero, A. P. Seitsonen, A. Smogunov, P. Umari,R. M. Wentzcovitch, *J. Phys.: Condens. Matter* **2009**, *21*, 395502.
[18] P. Giannozzi, O. Andreussi, T. Brumme, O. Bunau, M. Buongiorno Nardelli, M. Calandra, R. Car, C. Cavazzoni, D. Ceresoli, M. Cococcioni, N. Colonna, I. Carnimeo, A. Dal Corso, S. de Gironcoli, P. Delugas, R. A. DiStasio, A. Ferretti, A. Floris, G. Fratesi, G. Fugallo, R. Gebauer, U. Gerstmann, F. Giustino, T. Gorni, J. Jia, M. Kawamura, H. Y. Ko, A. Kokalj, E. Küçükbenli, M. Lazzeri, M. Marsili, N. Marzari, F. Mauri, N. L. Nguyen, H. V. Nguyen, A. Otero-de-la-Roza, L. Paulatto, S. Poncé, D. Rocca, R. Sabatini, B. Santra, M. Schlipf, A. P. Seitsonen, A. Smogunov, I. Timrov, T. Thonhauser, P. Umari, N. Vast, X. Wu,S. Baroni, *J. Phys.: Condens. Matter* **2017**, *29*, 465901.
[19] S. Baroni, S. d. Gironcoli, A. D. Corso,P. Giannozzi, *Rev. Mod. Phys.* **2001**, *73*, 515-562.
[20] P. B. Allen, R. C. Dynes, *Phys. Rev. B* **1975**, *12*, 905-922.
[21] A. V. Shapeev, *Multiscale Model. Simul.* **2016**, *14*, 1153-1173.
[22] V. V. Ladygin, P. Y. Korotaev, A. V. Yanilkin,A. V. Shapeev, *Comput. Mater. Sci.* **2020**, *172*, 109333.
[23] E. V. Podryabinkin, A. V. Shapeev, *Comput. Mater. Sci.* **2017**, *140*, 171-180.
[24] J. M. Dickey, A. Paskin, *Phys. Rev.* **1969**, *188*, 1407-1418.
[25] J. Appel, *Phys. Rev B* **1976**, *13*, 3203-3208.
[26] E. Talantsev, W. P. Crump,J. L. Tallon, *Ann. Phys.* **2017**, *529*, 1700197.
[27] E. F. Talantsev, J. L. Tallon, *Nat. Commun.* **2015**, *6*, 7820.

[28] Author. *BCS-theory-critical-current-fit*, https://github.com/WayneCrump/BCS-theory-critical-current-fit **2023**.
[29] D. Laniel, F. Trybel, B. Winkler, F. Knoop, T. Fedotenko, S. Khandarkhaeva, A. Aslandukova, T. Meier, S. Chariton, K. Glazyrin, V. Milman, V. Prakapenka, I. A. Abrikosov, L. Dubrovinsky,N. Dubrovinskaia, *Nat. Commun.* **2022**, *13*, 6987.
[30] A. Aslandukov, M. Aslandukov, N. Dubrovinskaia,L. Dubrovinsky, *J appl. crystallogr.* **2022**, *55*, 1383-1391.
[31] D. Laniel, B. Winkler, E. Bykova, T. Fedotenko, S. Chariton, V. Milman, M. Bykov, V. Prakapenka, L. Dubrovinsky,N. Dubrovinskaia, *Phys. Rev. B* **2020**, *102*.
[32] C. Prescher, V. B. Prakapenka, *High Pressure Res.* **2015**, *35*, 223-230.
[33] V. Petříček, M. Dušek,L. Palatinus, *Z. Kristallogr.* **2014**, *229*, 345-352.
[34] A. Le-Bail, *Powder Diffraction* **2005**, *20*, 316-326.
[35] M. I. Eremets, *J. Raman Spectrosc.* **2003**, *34*, 515-518.
[36] H. Mitamura, R. Watanuki, E. Kampert, T. Förster, A. Matsuo, T. Onimaru, N. Onozaki, Y. Amou, K. Wakiya, K. T. Matsumoto, I. Yamamoto, K. Suzuki, S. Zherlitsyn, J. Wosnitza, M. Tokunaga, K. Kindo,T. Sakakibara, *Review of Scientific Instruments* **2020**, *91*, 125107.
[37] D. V. Semenok, I. A. Troyan, A. V. Sadakov, D. Zhou, M. Galasso, A. G. Kvashnin, A. G. Ivanova, I. A. Kruglov, A. A. Bykov, K. Y. Terent'ev, A. V. Cherepakhin, O. A. Sobolevskiy, K. S. Pervakov, A. Y. Seregin, T. Helm, T. Förster, A. D. Grockowiak, S. W. Tozer, Y. Nakamoto, K. Shimizu, V. M. Pudalov, I. S. Lyubutin,A. R. Oganov, *Adv. Mater.* **2022**, *34*, 2204038.
[38] M. M. D. Esfahani, Z. Wang, A. R. Oganov, H. Dong, Q. Zhu, S. Wang, M. S. Rakitin,X. Zhou, *Sci. Rep.* **2016**, *6*, 22873.
[39] G. Gao, A. R. Oganov, P. Li, Z. Li, H. Wang, T. Cui, Y. Ma, A. Bergara, A. O. Lyakhov, T. Iitaka,G. Zou, *Proc. Natl. Acad. Sci. U S A* **2010**, *107*, 1317-1320.
[40] C. W. Glass, A. R. Oganov,N. Hansen, *Comput. Phys. Commun.* **2006**, *175*, 713-720.
[41] M. Bykov, S. Chariton, H. Fei, T. Fedotenko, G. Aprilis, A. V. Ponomareva, F. Tasnádi, I. A. Abrikosov, B. Merle, P. Feldner, S. Vogel, W. Schnick, V. B. Prakapenka, E. Greenberg, M. Hanfland, A. Pakhomova, H.-P. Liermann, T. Katsura, N. Dubrovinskaia,L. Dubrovinsky, *Nat. Commun.* **2019**, *10*, 2994.
[42] D. Semenok. *Computational design of new superconducting materials and their targeted experimental synthesis* Doctoral program in matraials science and engineering thesis, Skoltech, (2022).
[43] W. Chen, X. Huang, D. V. Semenok, S. Chen, D. Zhou, K. Zhang, A. R. Oganov,T. Cui, *Nat. Commun.* **2023**, *14*, 2660.
[44] D. Sun, V. S. Minkov, S. Mozaffari, Y. Sun, Y. Ma, S. Chariton, V. B. Prakapenka, M. I. Eremets, L. Balicas,F. F. Balakirev, *Nat. Commun.* **2021**, *12*, 6863.
[45] A. P. Drozdov, M. I. Eremets, I. A. Troyan, V. Ksenofontov,S. I. Shylin, *Nature* **2015**, *525*, 73-76.
[46] W. Chen, D. V. Semenok, A. G. Kvashnin, X. Huang, I. A. Kruglov, M. Galasso, H. Song, D. Duan, A. F. Goncharov, V. B. Prakapenka, A. R. Oganov,T. Cui, *Nat. Commun.* **2021**, *12*, 273.
[47] W. Chen, D. V. Semenok, X. Huang, H. Shu, X. Li, D. Duan, T. Cui,A. R. Oganov, *Phys. Rev. Lett.* **2021**, *127*, 117001.
[48] F. Bloch, *Z. Physik* **1930**, *59*, 208-214.
[49] E. Grüneisen, *Ann. Phys.* **1933**, *408*, 530-540.
[50] J. I. Mustafa, M. Bernardi, J. B. Neaton,S. G. Louie, *Phys. Rev. B* **2016**, *94*, 155105.
[51] P. Kapitza, E. Rutherford, *Proc. R. Soc. Lond. A* **1929**, *123*, 292-341.
[52] I. M. Lifshitz, V. G. Peschanskii, *Sov. Phys. JETP* **1959**, *8*, 875.
[53] A. Faghaninia, J. W. Ager,C. S. Lo, *Phys. Rev. B* **2015**, *91*, 235123.

[54] W. Sano, T. Koretsune, T. Tadano, R. Akashi,R. Arita, *Phys. Rev. B* **2016**, *93*, 094525.
[55] L. Van Hove, *Phys. Rev.* **1953**, *89*, 1189-1193.
[56] M. Kawamura, Y. Gohda,S. Tsuneyuki, *Phys. Rev. B* **2014**, *89*, 094515.
[57] I. Errea, M. Calandra,F. Mauri, *Phys. Rev. B* **2014**, *89*, 064302.
[58] P. M. Edwards, *J. Chem. Inf. Model.* **2002**, *42*, 1270-1271.
[59] I. Errea, F. Belli, L. Monacelli, A. Sanna, T. Koretsune, T. Tadano, R. Bianco, M. Calandra, R. Arita, F. Mauri,J. A. Flores-Livas, *Nature* **2020**, *578*, 66-69.
[60] I. Errea, M. Calandra, C. J. Pickard, J. R. Nelson, R. J. Needs, Y. Li, H. Liu, Y. Zhang, Y. Ma,F. Mauri, *Nature* **2016**, *532*, 81-84.
[61] D. Zhou, D. V. Semenok, H. Xie, A. I. Kartsev, A. G. Kvashnin, X. Huang, D. Duan, A. R. Oganov,T. Cui, *J. Am. Chem. Soc.* **2020**, *142*, 2803–2811.
[62] P. Hou, F. Belli, R. Bianco,I. Errea, *Phys. Rev. B* **2021**, *103*, 134305.
[63] P. Kong, V. S. Minkov, M. A. Kuzovnikov, A. P. Drozdov, S. P. Besedin, S. Mozaffari, L. Balicas, F. F. Balakirev, V. B. Prakapenka, S. Chariton, D. A. Knyazev, E. Greenberg,M. I. Eremets, *Nat. Commun.* **2021**, *12*, 5075.
[64] A. P. Drozdov, P. P. Kong, V. S. Minkov, S. P. Besedin, M. A. Kuzovnikov, S. Mozaffari, L. Balicas, F. F. Balakirev, D. E. Graf, V. B. Prakapenka, E. Greenberg, D. A. Knyazev, M. Tkacz,M. I. Eremets, *Nature* **2019**, *569*, 528-531.
[65] M. Somayazulu, M. Ahart, A. K. Mishra, Z. M. Geballe, M. Baldini, Y. Meng, V. V. Struzhkin,R. J. Hemley, *Phys. Rev. Lett.* **2019**, *122*, 027001.
[66] P. E. Blöchl, O. Jepsen,O. K. Andersen, *Phys. Rev. B* **1994**, *49*, 16223-16233.
[67] P. Ravindran, L. Fast, P. A. Korzhavyi, B. Johansson, J. Wills,O. Eriksson, *J. Appl. Phys.* **1998**, *84*, 4891-4904.
